# Three-Component Azidation of Styrene-Type Double Bonds: Light-Switchable Behavior of a Copper Photoredox Catalyst**

**DOI:** 10.1002/anie.201502980

**Published:** 2015-06-26

**Authors:** Gabriele Fumagalli, Pauline T G Rabet, Scott Boyd, Michael F Greaney

**Affiliations:** School of Chemistry, The University of Manchester Oxford Road, Manchester, M13 9PL (UK); Department of Oncology, AstraZeneca Alderley Park, Macclesfield, SK10 4TG (UK)

**Keywords:** azides, copper, heterocycles, photochemistry, radicals

## Abstract

[Cu(dap)_2_]Cl effectively catalyzes azide addition from the Zhdankin reagent to styrene-type double bonds, and subsequent addition of a third component to the benzylic position. In the presence of light, a photoredox cycle is implicated with polar components such as methanol or bromide adding to a benzylic cation. In the absence of light, by contrast, double azidation takes place to give diazides. Therefore, regioselective double functionalization can be achieved in good to excellent yields, with a switch between light and dark controlling the degree of azidation.

New azidation methods continue to stimulate reaction invention, and are driven by the exceptional versatility of the azide group in chemistry and biology.^1^, ^2^ Most methods for the installation of this fundamental building block use either highly toxic and/or explosive reagents, which are usually nucleophilic sources of azide. As an alternative, an electrophilic source of azide was reported in 1994 by Zhdankin and co-workers, in the form of the hypervalent iodine reagent 1-azido-1 *l*^*3*^-benzo[*d*][1,2]iodaoxol-3(1*H*)-one (**1**).^3^ In contrast to other iodine(I) and iodine(III) azides, **1** is a crystalline solid and thermally stable up to 130 °C, features which have been exploited in a range of pioneering azidation methods in recent literature.^4^

We were interested in employing **1** in a new way, using photoredox catalysis (PRC) to activate the reagent for alkene azidation. Application of the Zhdankin reagent to alkene functionalization is limited to two literature reports (Scheme 1):4c,g the group of Jiao reported an oxy azidation of indoles with **1** using copper catalysis, and the group of Studer described a more general alkene azidation using **1** in the presence of TEMPONa. This latter procedure does, however, require 3 equivalents of TEMPONa, a reactive reagent which must be freshly prepared using sodium metal, and it is also specific for introducing the tetramethylpiperidine oxide group. We reasoned that the power of PRC to enable new reactions under mild reaction conditions,^5^ particularly in the area of alkene functionalization,^6^ could substantially enhance the scope of this transformation. In addition, PRC for azidyl radical addition^7^ has yet to be investigated, and would represent a versatile and mild approach to valuable organic azides. PRC applications of azides to date have involved nitrene generation from vinyl or aryl azides.^8^ A single report from Masson, Magnier, and co-workers^9^ describes the trifluoromethyl azidation of double bonds using an azide anion introduced from TMSN_3_.

**scheme 1:**
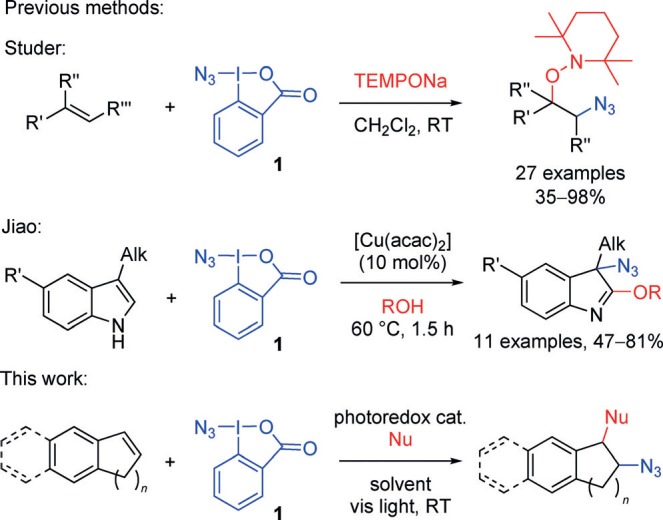
Application of the Zhdankin reagent 1 in alkene functionalization. acac=acetylacetonate, TEMPO=2,2,6,6-tetramethylpiperidin-1-oxyl.

Our plan was to investigate one-electron reduction of **1** using a suitable photoredox catalyst (Scheme 1), thus generating azidyl radicals, which we planned to capture in a three-component coupling^10^ based on our previous experience with alkoxy and amino arylations of styrenes.^11^

We began our investigation using 5 equivalents of styrene (**2 a**) and **1** as the limiting reagent, and 1 mol % of a photoredox catalyst in methanol as the solvent of choice (Table 1). Interestingly, the commonly used [Ir(ppy)_3_] and [Ru(bpy)_3_Cl_2_] photoredox catalysts failed to deliver any product in appreciable yields (entries 1 and 2). We were delighted to find, however, that the copper salt [Cu(dap)_2_]Cl [dap=2,9-bis(*p*-anisyl)-1,10-phenanthroline], successfully gave the azidomethoxylated **3 a** in 60 % yield (entry 3). First-row transition-metal complexes have seen limited application in PRC, as the lifetimes of their photoexcited state are generally too short to participate in the requisite electron-transfer cycles. Copper complexes of 2,9-substituted phenanthrolines are exceptional in this regard, with [Cu(dap)_2_]^+^ and related compounds being first prepared by the groups of McMillin and Sauvage, the latter being part of their seminal work on metal-template synthesis of interlocked architectures.^12^ The entwined ligand architecture enables an unusually long-lived photoexcited state, which was originally exploited in the dimerization of simple benzyl bromides under visible-light irradiation.12c This pioneering work has only recently been revisited in synthesis,^13^ with the groups of Reiser, Collins, Ollivier, and Dolbier exploiting copper/phenanthroline photocatalysts in atom-transfer radical addition, oxidative cyclization, and allylic arylation chemistry.^14^ Given the vast difference in cost between copper and typical noble-metal PRC complexes, we were keen to exploit our finding on [Cu(dap)_2_]Cl catalysis for azidomethoxylation. Further screening established an optimal stoichiometry of 5:1 styrene/**1** (entry 4). Reducing the catalyst loading led to a reduction in yield, whereas an increase in the catalyst loading did not affect the yield (entries 5 and 6). Control experiments showed that both the catalyst and degassed solvent were necessary for successful reaction (entries 7 and 8), whereas reaction in the dark unexpectedly generated the doubly azidated product **4 a** in excellent yield (entry 9) with only traces of **3 a**. Intrigued by the possibility of light-switchable catalysis, we first examined the scope of the photocatalyzed methoxy azidation reaction.

**Table 1:**
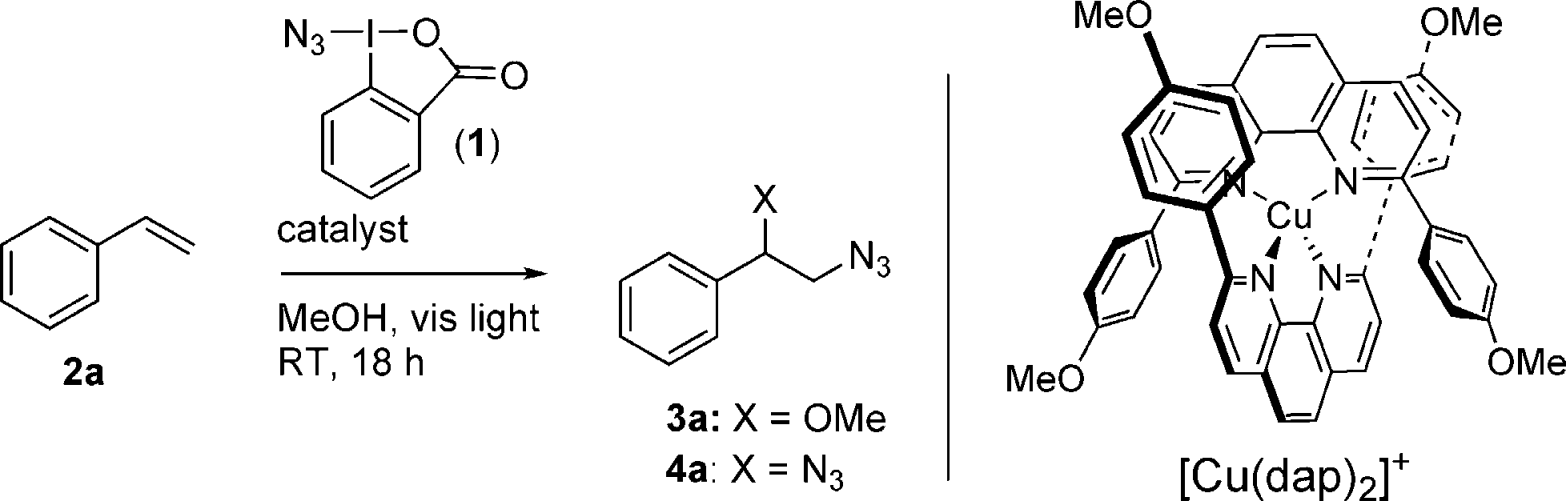
Reaction optimization.

Entry	Catalyst	2 a(equiv)	3 aYield [%]^[a]^
1	1 mol % [Ir(ppy)_3_]	5	decomposition
2	1 mol % [Ru(bpy)_3_]Cl_2_	5	no reaction
3	1 mol % [Cu(dap)_2_]Cl	5	60
4	1 mol % [Cu(dap)_2_]Cl	2	36
5	0.5 mol % [Cu(dap)_2_]Cl	5	30
6	2 mol % [Cu(dap)_2_]Cl	5	59
7	–	5	no reaction
8^[b]^	1 mol % [Cu(dap)_2_]Cl	5	no reaction
9^[d]^	1 mol % [Cu(dap)_2_]Cl	5	8^[c]^ (**4 a:** 94 %)

[a] Reactions were performed using 0.5 mmol of **1** in 5 mL of methanol and yields refer to isolated **3 a**.

[b] Reaction was performed with non-degassed methanol.

[c] Yield calculated from ^1^H NMR analysis of the crude reaction mixture.

[d] Reaction was performed in the dark. bpy=2,2′-bipyridine, ppy=2-phenylpyridine.

Upon investigating the photocatalyzed reaction (Scheme 2), it was observed that substitution on the arene ring is generally well tolerated (**3 a–e** and **3 n–p**), as are bulky *ortho*-substituents (e.g., the mesityl derivative **2 e** yielded the corresponding compound **3 e** in excellent yield). Compounds with stronger electron-donating substituents, such as methoxy groups, were good substrates, with *trans*-anetole yielding 88 % of the corresponding product **3 n** as a mixture of diastereomers, and β*-*vinylnaphthalene giving **3 f** in good yield. Mildly electron-poor styrenes were productive, with the *para-*fluoro and *para-*chloro substrates surviving the reaction conditions and giving good yields of the corresponding methoxyazidated products **3 o** and **3 p**. Substitutions on the styrene double bond at the α- and β-positions were tolerated, thus delivering the final compounds in good yields (**3 g–m**). Fused non-aromatic ring systems such as indene and 1,2-dihydronaphthalene underwent a smooth reaction, thus yielding **3 g** and **3 h** in good yields (the former as a single diastereomer), and 1-phenylcyclohexene afforded the secondary azide **3 k** in 60 % yield. Pleasingly, heteroaryl styrene analogues could be employed in the reaction, with 2-vinylbenzothiophene and 2-vinylbenzofuran providing the corresponding products **3 q** (80 %) and **3 r** (62 %) in and yield respectively. Unsuccessful substrates identified during screening were the strongly electron-poor styrenes (e.g. *p*-NO_2_ or pyridyl), phenylacetylene, and α,β-unsaturated ketones and esters.

**scheme 2:**
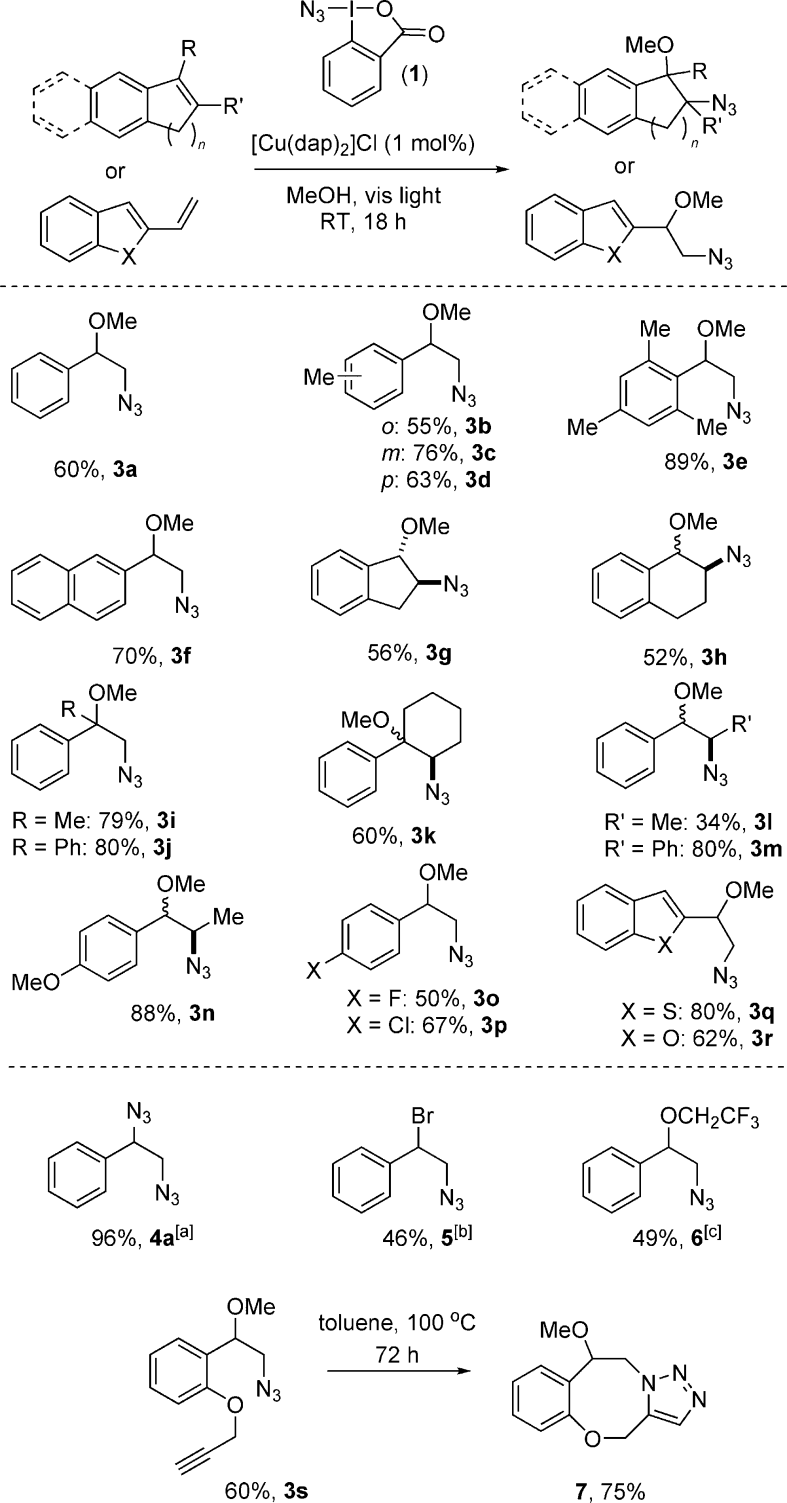
Styrene reaction scope. Reactions were performed using 0.5 mmol of 1, 2.5 mmol of styrene, and 0.005 mmol of [Cu(dap)_2_]Cl. For 3 a–s 5 mL of methanol was used as solvent. Yields are those of the isolated product. [a] Used 10 equivalents of NaN_3_ in MeCN. [b] Used 10 equivalents of NaBr in MeCN. [c] The fluorinated alcohol was used as the solvent.

With the styrene scope established, we then explored the possibility of using alternative nucleophilic components to methanol (Scheme 2). In contrast to our previous PRC arylation system,^11^ Ritter-type reactivity of nitrile components was not observed under these reaction conditions. As a result, we could use MeCN as a solvent for the reaction and successfully use simple inorganic salts as alternatives to oxygen nucleophiles. Use of NaN_3_ in combination with **1** resulted in double azidation in excellent yield (**4 a**). The synthesis of the α*-*bromo azide **5** was also possible in moderate yield, thus providing a useful substrate for further synthetic manipulation. Surprisingly, simple alcohols other than methanol were not successful in the reaction. We could, however, employ CF_3_CH_2_OH to give the perfluorinated alkyl ether **6** in 49 % yield. The functional-group compatibility of the process was further illustrated by an alkyne substrate, which undergoes successful methoxy azidation with the alkyne intact to deliver **3 s**. We could then exploit the tethered alkyne in an intramolecular azide cycloaddition, whereby simple heating in toluene gave the 1,4-oxazocine **7** in 75 % yield, a compound class of current interest in kinase medicinal chemistry.^15^

We then turned to the double azidation reaction which occurs in the dark. No reaction was observed upon re-exposure of the bis(azide) **4 a** (see Table 1) to the reaction conditions in the light, thus indicating that the methoxy azide **3 a** does not form through simple methanolysis of **4 a**. Additional control experiments with simple copper salts did produce **4 a** in varying amounts, but it was accompanied by significant quantities of side products. A survey of the styrene scope established that the reaction was effective for a range of styrenes (products **4 b**–**h**), 2-vinyl naphthalene (**4 i**), and indene (**4 j**), thus affording the vicinal diazides in good yield (Scheme 3). The neutral reaction conditions employed, at room temperature, are notably mild relative to literature methods for diazidation, which often features the azide anion and stoichiometric amounts of strong oxidants.^16^ These compounds are precursors to important 1,2-diamines, as demonstrated by the flow hydrogenation of **4 a** over Pd/C to give 1,2-(diamine)ethylbenzene in quantitative yield (see the Supporting Information).

**scheme 3:**
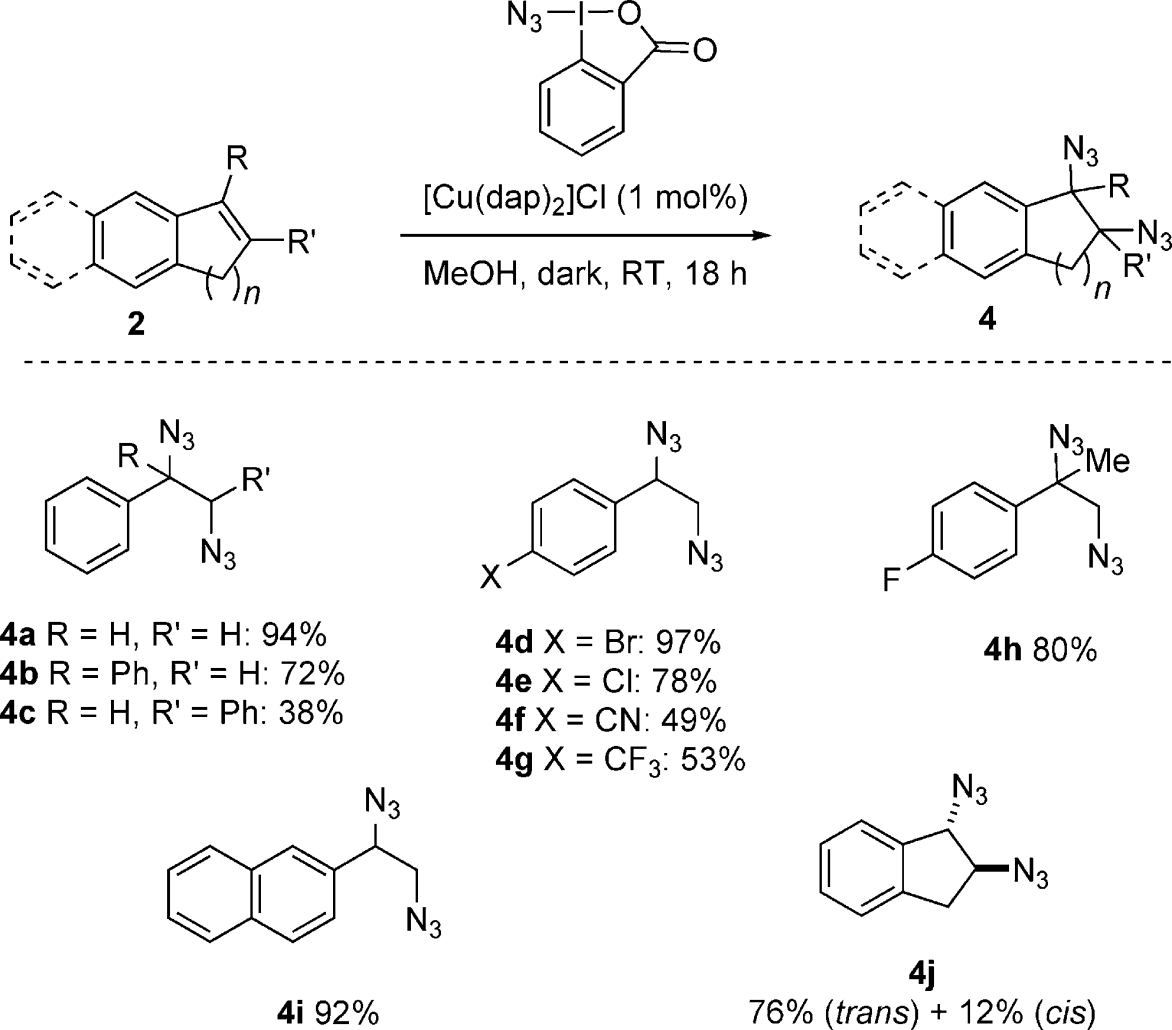
Copper-catalyzed double azidation in the dark. Reactions were performed using 0.5 mmol of 1, 2.5 mmol of styrene, and 0.005 mmol of [Cu(dap)_2_]Cl in methanol.

While full mechanistic details remain to be established, it seems likely that both pathways begin with an azide radical generation from the copper catalyst and **1**, and subsequent styrene addition. The Zhdankin reagent is known as a source of azide radicals by electron transfer from copper or iron salts,4a,c and no reaction was observed in the presence of either TEMPO or oxygen. The fate of the ensuing benzyl radical then diverges according to dark or light conditions. In the dark the lifetime is sufficient to add to another molecule of **1** to give the bis(azide) products **4**. In related work, Magnus and co-workers have described double radical azidation of triisoproylsilyl enol ethers using the combination of PhIO/TMSN_3_ (generating an ArIN_3_X species in situ) plus a catalytic amount of TEMPO.^17^ In the presence of light, however, oxidation to the benzylic cation15a,b takes place, and is trapped with methanol or other polar components to give the differentially substituted products.

To conclude, we have described a novel, light-switchable, azidation process for activated alkenes. The process is notable for using a sustainable and cheap copper-based photoredox catalyst, as opposed to noble-metal-based complexes, to enable electron transfer under very mild reaction conditions. The reaction is light-switchable, thus effecting double C–N bond formation in the dark, and C–N/C–O or C–Br formation in the light. Future studies will examine this mechanistic dichotomy in more detail, along with its exploitation in the rapid synthesis of nitrogen-containing small-molecule building blocks.

## Supporting Information

As a service to our authors and readers, this journal provides supporting information supplied by the authors. Such materials are peer reviewed and may be re-organized for online delivery, but are not copy-edited or typeset. Technical support issues arising from supporting information (other than missing files) should be addressed to the authors.

miscellaneous_information
