# Supplementary material for: Three-Component Azidation of Styrene-Type Double Bonds: Light-Switchable Behavior of a Copper Photoredox Catalyst
Source: Angew Chem Int Ed Engl. 2015 Jun 26;54(39):11481–4. doi: 10.1002/anie.201502980 (PMC4678422; doi:10.1002/anie.201502980)
Supplement: Supplementary file 1 [file anie0054-11481-sd1.pdf]

Angewandte Chemie
Eine Zeitschrift der Gesellschaft Deutscher Chemiker

Supporting Information

Three-Component Azidation of Styrene-Type Double Bonds: Light-Switchable Behavior of a Copper Photoredox Catalyst**

*Gabriele Fumagalli, Pauline T. G. Rabet, Scott Boyd, and Michael F. Greaney**

anie_201502980_sm_miscellaneous_information.pdf

Supporting Information

Table of Contents

1 General information 2
2 Optimisation screenings 3
2.1 Catalyst screening 3
2.2 Additive Screening 4
2.3 Stoichiometry assessment 5
2.4 Control reactions 6
2.5 Time-Control Experiments 8
2.6 Nucleophiles addition 9
3 Typical procedures for the three component azido-functionalisation of styrenes 10
4 Characterisation data for methoxyazidated compounds 3a – 3s and oxazocine 5 11
5 Characterisation data for azido-functionalised compounds 4a, 5 and 6 18
6 Characterisation data for diazido-functionalised compounds 4a –4j and dimaine 8 19
7 ^{1}H NMR, ^{13}C NMR and IR spectra for methoxyazidated compounds 3a – 3s and oxazocine 5 23
8 ^{1}H NMR, ^{13}C NMR and IR spectra for azido-functionalised compounds 4a, 5 and 6 69
9 ^{1}H NMR, ^{13}C NMR and IR spectra for azido-functionalised compounds 4a - 4j 74
10 References 86

1 General information

^{1}H-NMR, ^{13}C{^{1}H}-NMR and ^{19}F-NMR were recorded at 500/400 MHz, 125/100 MHz, 470/376 MHz on a Bruker Avance 500 or 400 spectrometers. All spectra are referenced to $CDCl_3$ residual $CHCl_3$ peak (^{1}H-NMR δ = 7.26 ppm; ^{13}C{^{1}H}-NMR δ = 77.0 ppm). All chemical shifts are quoted in parts per million (ppm), measured from the centre of the signal except in the case of multiplets of more than one proton, which are quoted as a range. Coupling constants are quoted to the nearest 0.1 Hz. Splitting patterns are abbreviated as follows: singlet (s), doublet (d), triplet (t), quartet (q), quintet (quin.), heptet (hept.), multiplet (m), broad singlet (brs) and combinations thereof.

Infrared spectra were recorded on a spectrometer as neat using a Perkin-Elmer FT-IR Spectrum RX1 or BX spectrometers.

LCMS analysis was performed on a Agilent 1200 series fitted with a 3.0 x 20 mm, C18, 3.0 μm column, with a single quadrupole Agilent 6100 mass spectrometrer, with ESI ionisation. Elution was carried out using reverse phase gradient of MeOH-water with 0.1% formic acid.

Low resolution mass spectrometry was performed on an Agilent 6100 mass spectrometer (ESI ionisation) and Hewlett Packard 5971 MSD (GC/MS with EI). High resolution mass spectrometry was performed on a Waters QTOF with ESI/APCI ionisation and a Thermo Finnigan MAT95XP (EI).

Melting points were determined using a Kofler hot-stage apparatus or Stuart Scientific SMP10 apparatus and are uncorrected. Thin layer chromatography (TLC) was performed using pre-coated Merck 60F254 silica plates. Visualization was performed using either UV light or treatment with acidic potassium permanganate. Flash chromatography was performed using Merck Kieselgel (mesh size 220-240) silica.

All reagents and solvents were used as obtained from commercial source, unless otherwise stated. Solvents were degassed by bubbling nitrogen gas through them for twenty minutes prior to use.

1-azido-1λ^3-benzo[*d*][1,2]iodaoxol-3(*1H*)-one,[1] 2-vinylbenzofuran,[2] Cu(dap)$_2$Cl,[3] 2-vinylthiophene[4] and 2-(prop-2-yn-1-yloxy)styrene[5] were synthesised according to literature procedures.

***Safety**: Azides are potentially explosive compounds and require appropriate safety protocols to be observed at all times.*[6]

N.B. Whilst 1-Azido-1λ^3-benzo[d][1,2]iodaoxol-3(1H)-one, ***1****, is more stable than other iodine azide reagents, it is reported to decompose with explosion at 138-139°C.*[7] *In the course of reaction optimisation for this work, we observed an explosive decomposition of reagent* ***1*** *(100 mg, (s)) in the presence of excess KSCN (s) in a vial at room temperature.*

2 Optimisation screenings

2.1 Catalyst screening

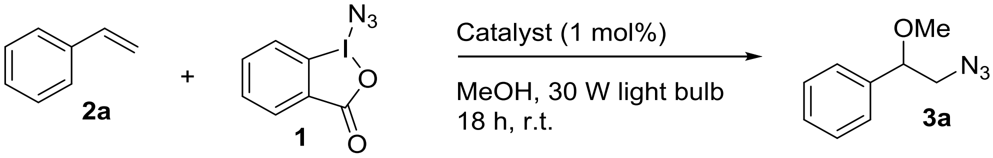

**Entry**	**Catalyst**	**Yield**[a]
**1**	$Ir(ppy)_3$	dcmp – trace of product
**2**	$Ir(dtbbpy)(ppy)_2PF_6$	-
**3**	$Ru(bpy)_3Cl_2$	-
**4**	Eosin Y	-
**5**	Methylene Blue	-
**6**	Fluorescein	-
**7**	Rhodamine 6G	-
**8**	Rose Bengal	-
**9**	Acridinium **2**	traces
**10**	$Cu(dap)_2Cl$	60%

Table 1: [a] Isolated yields.

A vial containing the photoredox catalyst (5 μmol, 0.01 eq), 1-azido-1λ^3-benzo[*d*][1,2]iodaoxol-3(*1H*)-one (145 mg, 0.5 mmol, 1 eq) and equipped with a stirrer bar, sealed with a septum, was purged under a flux of nitrogen. Then degassed methanol (5 mL) and styrene (290 μL, 2.5 mmol, 5 eq) were added and the tube was sealed and put 1 cm away from a 30 W fluorescent bulb and stirred for 18 hours. The reaction mixture was loaded on silica and purified by flash column chromatography eluting with a mixture hexane:ethyl acetate = 100:0 to 95:5.

2.2 Additive Screening

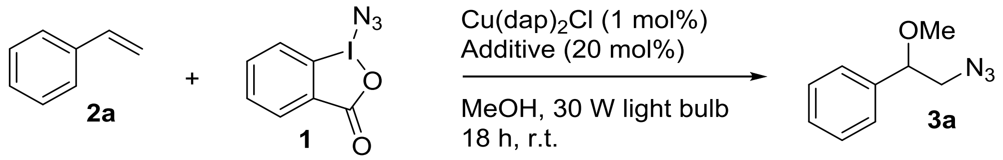

Entry	Additive	Yield[a]
**1**	$Zn(OAc)_2$	44%
**2**	KOAc	< 5%
**3**	$Ni(OAc)_2$	< 5%
**4**	$Co(OAc)_2$	< 5%
**5**	K_2CO_3	< 5%
**6**	$ZnCO_3$	< 5%
**7**	$Ti(O^iPr)_4$	< 5%
**8**	$Ag(O_2CCF_3)$	< 5%
**9**	$Mn(OAc)_3$	50%
**10**	$FeSO_4$	58%
**11**	$Zn(ClO_4)_2$	50%

Table 2: [a] Isolated yields.

A vial containing $Cu(dap)_2Cl$ (4.4 mg, 5 μmol, 0.01 eq), 1-azido-1λ^3-benzo[*d*][1,2]iodaoxol-3(*1H*)-one (145 mg, 0.5 mmol, 1 eq), the additive and equipped with a stirrer bar, sealed with a septum, was purged under a flux of nitrogen. Then degassed methanol (5 mL) and styrene (290 μL, 2.5 mmol, 5 eq) were added and the tube was sealed and put 1 cm away from a 30 W fluorescent bulb and stirred for 18 hours. The reaction mixture was loaded on silica and purified by flash column chromatography eluting with a mixture hexane:ethyl acetate = 100:0 to 95:5.

2.3 Stoichiometry assessment

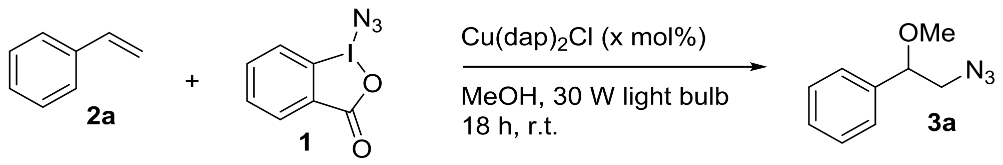

Entry	Eq 1a	Eq 2a	X	Yield[a]
**1**	5	1	1 mol%	60%
**2**	2	1	1 mol%	36%
**3**	10	1	1 mol%	58%
**4**	5	1	0.5 mol%	30%
**5**	5	1	2 mol%	59%

Table 3: Conditions: 1eq. = 0.5 mmol. [a] Isolated yields.

A vial containing Cu(dap)$_2$Cl, 1-azido-1λ^3-benzo[*d*][1,2]iodaoxol-3(*1H*)-one (145 mg, 0.5 mmol, 1 eq) and equipped with a stirrer bar, sealed with a septum, was purged under a flux of nitrogen. Then degassed methanol (5 mL) and styrene (290 µL, 2.5 mmol, 5 eq) were added and the tube was sealed and put 1 cm away from a 30 W fluorescent bulb and stirred for 18 hours. The reaction mixture was loaded on silica and purified by flash column chromatography eluting with a mixture hexane:ethyl acetate = 100:0 to 95:5.

2.4 Control reactions

A) Methoxyazidation control reactions

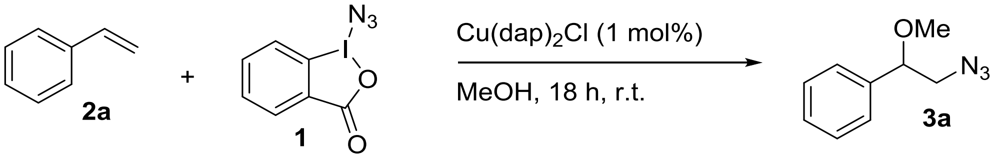

Entry	Cu(dap)$_2$Cl	Light bulb	Yield[a]
**1**	Y	Y	61%[b]
**2[c,e]**	Y	N	8%
**3**	N	Y	-
**4[d]**	Y	Y	-
**5[f]**	N	Y	-

Table 4: [a] Isolated yield. [b] Yield is the average of 5 reactions. [c] ^{1}H NMR yield. [d] Non-degassed methanol was used to perform the reaction. [e] Diazidated product was isolated in 94% yield. [f] Cu(acac)$_2$ was used instead of the photoredox catalyst.

A vial containing Cu(dap)$_2$Cl (4.4 mg, 5 μmol, 0.01 eq), 1-azido-1λ^3-benzo[*d*][1,2]iodaoxol-3(*1H*)-one (145 mg, 0.5 mmol, 1 eq) and equipped with a stirrer bar, sealed with a septum, was purged under a flux of nitrogen. Then degassed methanol (5 mL) and styrene (290 μL, 2.5 mmol, 5 eq) were added and the tube was sealed and put 1 cm away from a 30 W fluorescent bulb and stirred for 18 hours. The reaction mixture was loaded on silica and purified by flash column chromatography eluting with a mixture hexane:ethyl acetate = 100:0 to 95:5.

B) Diazidation control reaction

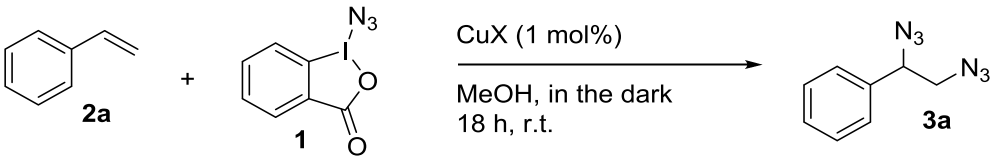

Entry	CuX	Yield[a]
**1**	Cu(dap)$_2$Cl	94%
**2[b]**	Cu(acac)$_2$	< 80%
**3[b]**	CuCl	< 80%
**4[b,c]**	CuCl	< 80%

Table 5: [a] Isolated yield. [b] The reaction yields a complex mixture containing the product other unknown compounds. Based on the mass of the sample retrieved after column chromatography, we can quote a yield range. [c] Reaction performed under visible light irradiation with a 30 W light bulb.

A vial containing Cu(dap)$_2$Cl (4.4 mg, 5 μmol, 0.01 eq), 1-azido-1λ^3-benzo[*d*][1,2]iodaoxol-3(*1H*)-one (145 mg, 0.5 mmol, 1 eq) and equipped with a stirrer bar, sealed with a septum, was purged

under a flux of nitrogen. Then degassed methanol (5 mL) and styrene (290 μL, 2.5 mmol, 5 eq) were added in the dark and the tube was sealed and stirred for 18 hours. The reaction mixture was loaded on silica and purified by flash column chromatography eluting with a mixture hexane:ethyl acetate = 100:0 to 98:2.

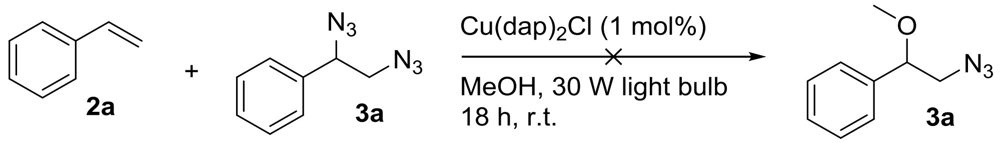

A vial containing $Cu(dap)_2Cl$ (4.4 mg, 5 μmol, 0.01 eq), 1,2-diazidoethylbenzene (X mg, X mmol, 1 eq) and equipped with a stirrer bar, sealed with a septum, was purged under a flux of nitrogen. Then degassed methanol (5 mL) and styrene (290 μL, 2.5 mmol, 5 eq) were added and the tube was sealed and put 1 cm away from a 30 W fluorescent bulb and stirred for 18 hours. The reaction mixture was loaded on silica and purified by flash column chromatography eluting with a mixture hexane:ethyl acetate = 100:0 to 98:2.

C) TEMPO control reaction

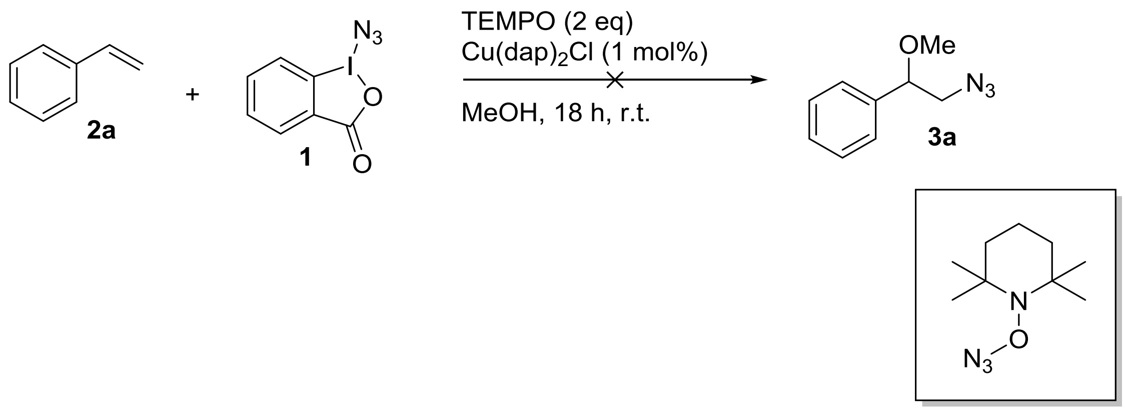

Identified by GC-MS

A vial containing $Cu(dap)_2Cl$ (4.4 mg, 5 μmol, 0.01 eq), 1-azido-1λ^3-benzo[*d*][1,2]iodaoxol-3(*1H*)-one (145 mg, 0.5 mmol, 1 eq) and equipped with a stirrer bar, sealed with a septum, was purged under a flux of nitrogen. Then a solution of TEMPO (156 mg, 1 mmol, 2 eq) in degassed methanol (5 mL) and styrene (290 μL, 2.5 mmol, 5 eq) were added and the tube was sealed and put 1 cm away from a 30 W fluorescent bulb and stirred for 18 hours.

2.5 Time-Control Experiments

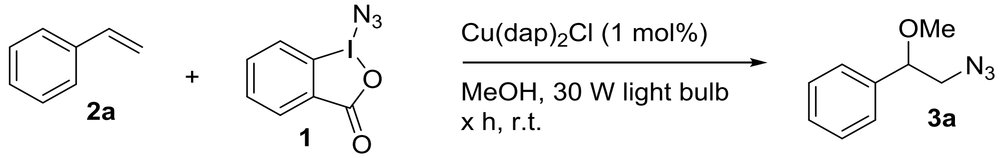

Entry	Eq 1a	Eq 2a	Time	Yield[a]
**1**	5	1	0.5 h	27%
**2**	5	1	1 h	45%
**3**	5	1	2 h	46%
**4**	5	1	3 h	50%
**5**	5	1	5 h	54%
**6**	5	1	18 h	60%

Table 5: [a] Isolated yield

A vial containing Cu(dap)$_2$Cl (4.4 mg, 5 μmol, 0.01 eq), 1-azido-1λ^3-benzo[*d*][1,2]iodaoxol-3(*1H*)-one (145 mg, 0.5 mmol, 1 eq) and equipped with a stirrer bar, sealed with a septum, was purged under a flux of nitrogen. Then degassed methanol (5 mL) and styrene (290 μL, 2.5 mmol, 5 eq) were added and the tube was sealed and put 1 cm away from a 30 W fluorescent bulb and stirred for a certain number of hours. The reaction mixture was loaded on silica and purified by flash column chromatography eluting with a mixture hexane:ethyl acetate = 100:0 to 95:5.

2.6 *Nucleophiles addition*

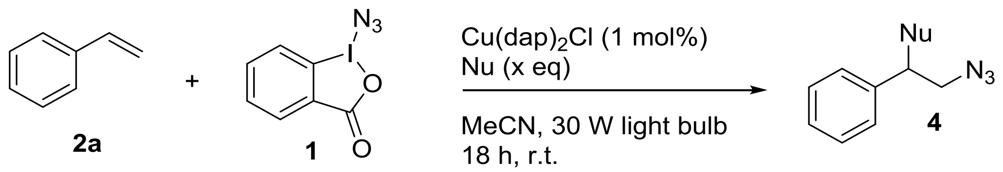

Entry	Nu	Eq Nu	Yield[a]
**1**	NaN_3	5	70%
**2**	NaN_3	10	96%
**3**	NaBr	10	46%
**4**	LiBr	10	< 5%
**5**	KBr	10	< 5%
**6**	TBAB	10	nr

Table 5: [a] Isolated yield

A vial containing $Cu(dap)_2Cl$ (4.4 mg, 5 μmol, 0.01 eq), 1-azido-1λ^3-benzo[*d*][1,2]iodaoxol-3(*1H*)-one (145 mg, 0.5 mmol, 1 eq), a suitable nucleophile and equipped with a stirrer bar, sealed with a septum, was purged under a flux of nitrogen. Then degassed acetonitrile (5 mL) and styrene (290 μL, 2.5 mmol, 5 eq) were added and the tube was sealed and put 1 cm away from a 30 W fluorescent bulb and stirred for a certain number of hours. The reaction mixture was loaded on silica and purified by flash column chromatography eluting with a mixture hexane:ethyl acetate = 100:0 to 95:5.

3 Typical procedures for the three component azido-functionalisation of styrenes

General procedure A

A vial containing $Cu(dap)_2Cl$ (4.4 mg, 5 μmol, 0.01 eq), 1-azido-1λ^3-benzo[*d*][1,2]iodaoxol-3(*1H*)-one (145 mg, 0.5 mmol, 1 eq) and equipped with a stirrer bar, sealed with a septum, was purged under a flux of nitrogen. Then degassed methanol (5 mL) and the styrene (2.5 mmol, 5 eq) were added and the tube was sealed and put 1 cm away from a 30 W fluorescent bulb and stirred for 18 hours. The reaction mixture was loaded on silica and purified by flash column chromatography eluting with a mixture hexane:ethyl acetate = 100:0 to 95:5.

General procedure B (for the trapping of different solvents)

A vial containing $Cu(dap)_2Cl$ (4.4 mg, 5 μmol, 0.01 eq), 1-azido-1λ^3-benzo[*d*][1,2]iodaoxol-3(*1H*)-one (145 mg, 0.5 mmol, 1 eq) and equipped with a stirrer bar, sealed with a septum, was purged under a flux of nitrogen. Then degassed solvent (5 mL) and the styrene (2.5 mmol, 5 eq) were added and the tube was sealed and put 1 cm away from a 30 W fluorescent bulb and stirred for 18 hours. The reaction mixture was loaded on silica and purified by flash column chromatography eluting with a mixture hexane:ethyl acetate = 100:0 to 95:5.

General procedure C (for the trapping of different nucleophiles)

A vial containing $Cu(dap)_2Cl$ (4.4 mg, 5 μmol, 0.01 eq), 1-azido-1λ^3-benzo[*d*][1,2]iodaoxol-3(*1H*)-one (145 mg, 0.5 mmol, 1 eq), the nucleophile (5 mmol, 10 eq) and equipped with a stirrer bar, sealed with a septum, was purged under a flux of nitrogen. Then degassed acetonitrile (5 mL) and the styrene (2.5 mmol, 5 eq) were added and the tube was sealed and put 1 cm away from a 30 W fluorescent bulb and stirred for 18 hours. The reaction mixture was loaded on silica and purified by flash column chromatography eluting with a mixture hexane:ethyl acetate = 100:0 to 95:5.

General procedure D (for the diazidation)

A vial containing $Cu(dap)_2Cl$ (4.4 mg, 5 μmol, 0.01 eq), 1-azido-1λ^3-benzo[*d*][1,2]iodaoxol-3(*1H*)-one (145 mg, 0.5 mmol, 1 eq) and equipped with a stirrer bar, sealed with a septum, was covered in aluminium foil and purged under a flux of nitrogen. Then degassed methanol (5 mL) and the styrene (2.5 mmol, 5 eq) were added and the tube was sealed the reaction mixture stirred for 18 hours in the dark. The reaction mixture was loaded on silica and purified by flash column chromatography eluting with a mixture hexane:ethyl acetate = 100:0 to 98:2.

4 Characterisation data for methoxyazidated compounds 3a – s and oxazocine 7

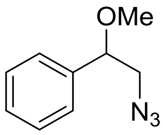

(2-azido-1-methoxyethyl)benzene (**3a**)

Prepared according to general procedure A reacting styrene (290 µL). The title compound was obtained as a colourless oil (53 mg, 60%). Data are in accordance with previous literature reports.[8] ^{1}H NMR (500 MHz, Chloroform-*d*) δ 7.44 – 7.36 (m, 2H), 7.37 – 7.30 (m, 3H), 4.36 (dd, *J* = 8.5, 3.6 Hz, 1H), 3.49 (dd, *J* = 13.0, 8.5 Hz, 1H), 3.32 (s, 3H), 3.21 (dd, *J* = 13.0, 3.6 Hz, 1H). ^{13}C NMR (125 MHz, Chloroform-*d*) δ 138.5, 128.8, 128.5, 126.7, 83.2, 57.0, 56.6. IR (neat film): 2927, 2096, 1453, 1107, 700. MS (EI): 177 (M, 0.1), 149 (M - N_2, 0.9), 121 (M - CH_2N_3, 100). HRMS (EI): Calculated for $C_9H_{11}NO$ (M - N_2): 149.0835; Found: 149.0832.

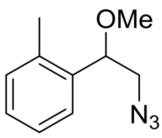

1-(2-azido-1-methoxyethyl)-2-methylbenzene (**3b**)

Prepared according to general procedure A reacting 2-methylstyrene (330 µL). The title compound was obtained as a colourless oil (53 mg, 55%). ^{1}H NMR (500 MHz, Chloroform-*d*) δ 7.40 (dd, *J* = 7.4, 1.7 Hz, 1H), 7.30 – 7.23 (m, 1H), 7.22 (td, *J* = 7.2, 1.7 Hz, 1H), 7.18 – 7.13 (m, 1H), 4.64 (dd, *J* = 8.8, 3.2 Hz, 1H), 3.44 (dd, *J* = 13.2, 8.8 Hz, 1H), 3.30 (s, 3H), 3.15 (dd, *J* = 13.2, 3.2 Hz, 1H), 2.33 (s, 3H). ^{13}C NMR (125 MHz, Chloroform-*d*) δ 136.4, 135.5, 130.7, 128.0, 126.5, 126.0, 79.9, 56.9, 55.6, 19.0. IR (neat film): 2925, 2099, 1460, 1104, 757. MS (EI): 163 (M - N_2, 2), 135 (M - CH_2N_3, 100). HRMS (EI): Calculated for $C_9H_{11}O$ (M – CH_2N_3): 135.0804; Found: 135.0807.

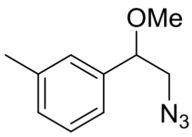

1-(2-azido-1-methoxyethyl)-3-methylbenzene (**3c**)

Prepared according to general procedure A reacting 3-methylstyrene (340 µL). The title compound was obtained as a colourless oil (73 mg, 76%). ^{1}H NMR (500 MHz, Chloroform-*d*) δ 7.31 – 7.24 (m, 1H), 7.19 – 7.06 (m, 3H), 4.32 (dd, *J* = 8.8, 3.5 Hz, 1H), 3.48 (dd, *J* = 13.0, 8.8 Hz, 1H), 3.31 (s, 3H), 3.19 (dd, *J* = 13.0, 3.5 Hz, 1H), 2.37 (s, 3H). ^{13}C NMR (125 MHz, Chloroform-*d*) δ 138.5, 138.4, 129.2, 128.6, 127.3, 123.8, 83.2, 56.9, 56.6, 21.5. IR (neat film): 2924, 2097, 1453, 1111, 704. MS (EI): 163 (M - N_2, 4), 135 (M - CH_2N_3, 100). HRMS (EI): Calculated for $C_9H_{11}O$ (M – CH_2N_3): 135.0804; Found: 135.0807.

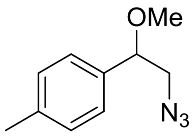

1-(2-azido-1-methoxyethyl)-4-methylbenzene (**3d**)

Prepared according to general procedure A reacting 4-methylstyrene (330 μL). The title compound was obtained as a colourless oil (60 mg, 63%). ^{1}H NMR (400 MHz, Chloroform-*d*) δ 7.20 (s, 4H), 4.33 (dd, *J* = 8.6, 3.6 Hz, 1H), 3.48 (dd, *J* = 13.0, 8.6 Hz, 1H), 3.30 (s, 3H), 3.19 (dd, *J* = 13.0, 3.6 Hz, 1H), 2.36 (s, 3H). ^{13}C NMR (100 MHz, Chloroform-*d*) δ 138.2, 135.5, 129.4, 126.6, 83.0, 56.8, 56.6, 21.2. IR (neat film): 2925, 2094, 1448, 1253, 1107, 813. MS (EI): 163 (M - N_2, 3), 135 (M - CH_2N_3, 100). HRMS (APCI): Calculated for $C_{10}H_{14}NO$ (M + H^+ - N_2): 164.1075; Found: 164.1074.

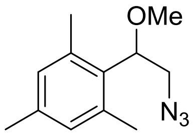

1-(2-azido-1-methoxyethyl)-2,4,6-trimethylbenzene (**3e**)

Prepared according to general procedure A reacting 2,4,6-trimethylstyrene (403 μL). The title compound was obtained as a colourless oil (97 mg, 89%). ^{1}H NMR (400 MHz, Chloroform-*d*) δ 6.84 (s, 2H), 4.85 (dd, *J* = 9.6, 3.8 Hz, 1H), 3.76 (dd, *J* = 13.1, 9.6 Hz, 1H), 3.26 (s, 3H), 3.15 (dd, *J* = 13.1, 3.8 Hz, 1H), 2.37 (s, 6H), 2.26 (s, 3H). ^{13}C NMR (100 MHz, Chloroform-*d*) δ 137.5, 136.9, 130.4, 130.3 (broad signal), 80.2, 56.4, 53.7, 20.8, 20.5 (broad signal). IR (neat film): 2926, 2095, 1449, 1253, 1110, 850. MS (EI): 191 (M - N_2, 20), 163 (M - CH_2N_3, 100). HRMS (APCI): Calculated for $C_{12}H_{18}NO$ (M + H^+ - N_2): 192.1359; Found: 192.1394.

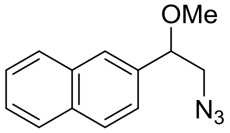

2-(2-azido-1-methoxyethyl)naphthalene (**3f**)

Prepared according to general procedure A reacting 2-vinylnaphthalene (385 mg). The title compound was obtained as a yellow oil (110 mg, 70 %). ^{1}H NMR (400 MHz, Chloroform-*d*) δ 7.90 – 7.82 (m, 3H), 7.79 (s, 1H), 7.56 – 7.47 (m, 2H), 7.44 (dd, *J* = 8.4, 1.9 Hz, 1H), 4.54 (dd, *J* = 8.5, 3.6 Hz, 1H), 3.59 (dd, *J* = 13.0, 8.5 Hz, 1H), 3.36 (s, 3H), 3.30 (dd, *J* = 13.0, 3.6 Hz, 1H). ^{13}C NMR (100 MHz, Chloroform-*d*) δ 136.0, 133.4, 133.2, 128.7, 127.9, 127.8, 126.4, 126.3, 126.2, 124.1, 83.3, 57.1, 56.6. IR (neat film): 3056, 2926, 2097, 1442, 1255, 1106, 810, 745. MS (ESI): 250 (M + Na^+). HRMS (APCI): Calculated for $C_{13}H_{14}NO$ (M + H^+ - N_2): 200.1075; Found: 200.1064.

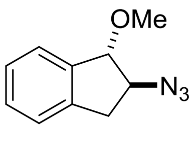

2-azido-1-methoxy-2,3-dihydro-1*H*-indene (**3g**)

Prepared according to general procedure A reacting indene (292 μL). The title compound was obtained as a colourless oil (54 mg, 56%), contaminated with small amounts of the diazide **4a**. *Trans*-diastereomer: ^{1}H NMR (400 MHz, Chloroform-*d*) δ 7.38 (dd, *J* = 7.1, 1.6 Hz, 1H), 7.33 – 7.15

(m, 3H), 4.71 (d, J = 4.7 Hz, 1H), 4.17 (ddd, J = 7.4, 6.0, 4.7 Hz, 1H), 3.58 (s, 3H), 3.37 (dd, J = 16.1, 7.4 Hz, 1H), 2.90 (dd, J = 16.1, 6.0 Hz, 1H). ^{13}C NMR (100 MHz, Chloroform-*d*) δ 139.9, 139.3, 129.1, 127.3, 125.1, 125.0, 88.5, 66.9, 57.7, 35.9. IR (neat film, significant peaks): 2926, 2097, 1461, 1259, 1093, 747. MS (ESI): 212 (M + Na$^+$). HRMS (APCI): Calculated for $C_{10}H_{12}NO$ (M + H$^+$ - N_2): 162.0919; Found: 162.0921.

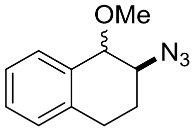

2-azido-1-methoxy-1,2,3,4-tetrahydronaphthalene (**3h**)

Prepared according to general procedure A reacting 1,2-dihydronaphthalene (326 μL). The two diastereomers of the title compound were obtained as colourless oils (53 mg, *cis:trans* = 1.4:1, 52%). IR (neat film, significant peaks): 2933, 2091, 1454, 1253, 1081, 746. MS (EI): 175 (M - N_2, 50), 160 (M - HN_3, 100), 132 (M – $C_2H_4N_3$, 90). HRMS (APCI): Calculated for $C_{11}H_{14}NO$ (M + H$^+$ - N_2): 176.1081; Found: 176.1075.
Cis-diastereomer (major): ^{1}H NMR (400 MHz, Chloroform-*d*) δ 7.38 (dd, J = 5.3, 3.8 Hz, 1H), 7.29 – 7.18 (m, 2H), 7.13 – 7.10 (m, 1H), 4.29 (d, J_{eq-ax} = 6.4 Hz, 1H), 3.96 (ddd, J = 8.5, 6.4, 3.4 Hz, 1H), 3.56 (s, 3H), 3.06 (ddd, J = 17.3, 6.3, 4.7 Hz, 1H), 2.95 – 2.78 (m, 1H), 2.27 – 2.18 (m, 1H), 1.92 (dddd, J = 13.5, 8.6, 7.3, 6.2 Hz, 1H). ^{13}C NMR (100 MHz, Chloroform-*d*) δ 136.0, 134.2, 129.3, 128.8, 128.0, 126.3, 80.4, 60.1, 57.6, 26.1, 24.9.
Trans-diastereomer (minor): ^{1}H NMR (400 MHz, Chloroform-*d*) δ 7.33 (dd, J = 7.5, 1.6 Hz, 1H), 7.29 – 7.20 (m, 2H), 7.18 – 7.14 (m, 1H), 4.33 (d, J_{eq-eq} = 3.1 Hz, 1H), 3.75 (dt, J = 10.5, 3.1 Hz, 1H), 3.54 (s, 3H), 2.91 – 2.84 (m, 2H), 2.34 (dddd, J = 12.9, 10.5, 8.9, 6.5 Hz, 1H), 2.01 – 1.96 (m, 1H). ^{13}C NMR (100 MHz, Chloroform-*d*) δ 135.6, 134.1, 129.0, 128.6, 128.4, 125.8, 79.4, 58.8, 57.4, 26.9, 22.7.

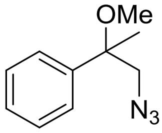

(1-azido-2-methoxypropan-2-yl)benzene (**3i**)

Prepared according to general procedure A reacting α-methylstyrene (325 μL). The title compound was obtained as a colourless oil (71 mg, 79%). Data are in accordance with previous literature reports.[9] ^{1}H NMR (400 MHz, Chloroform-*d*) δ 7.46 – 7.35 (m, 4H), 7.36 – 7.28 (m, 1H), 3.45 (d, J = 12.5 Hz, 1H), 3.21 (d, J = 12.5 Hz, 1H), 3.14 (s, 3H), 1.65 (s, 3H). ^{13}C NMR (125 MHz, Chloroform-*d*) δ 141.8, 128.5, 127.8, 126.4, 79.5, 61.7, 50.6, 20.1. IR (neat film): 2932, 2097, 1446, 1073, 700. MS (EI): 135 (M - CH_2N_3, 100). HRMS (EI): Calculated for $C_9H_{11}O$ (M – CH_2N_3): 135.0804; Found: 135.0806.

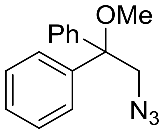

2-azido-1,1-diphenyl-1-methoxyethane (**3j**)

Prepared according to general procedure A reacting α-phenylstyrene (441 μL). The title compound were obtained as a colourless oil (102 mg, 80%). ^{1}H NMR (400 MHz, Chloroform-*d*) δ 7.40 – 7.23 (m, 10H), 4.06 (s, 2H), 3.16 (s, 3H). ^{13}C NMR (100 MHz, Chloroform-*d*) δ 142.2, 128.2, 127.6, 127.2, 82.8, 55.7, 51.2. IR (neat film): 2938, 2093, 1447, 1076, 715. MS (EI): 197 (M - CH_2N_3). HRMS (APCI): Calculated for $C_{15}H_{15}O$ (M – N_3): 211.1123; Found: 211.1109.

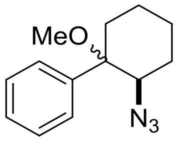

(2-azido-1-methoxycyclohexyl)benzene (**3k**)

Prepared according to general procedure A reacting 1-phenylcyclohexene (398 μL). The title compound was obtained as a colourless oil (70 mg, 60%).
Cis-diastereomer (29 mg, 25%): ^{1}H NMR (400 MHz, Chloroform-*d*) δ 7.49 – 7.28 (m, 5H), 3.74 – 3.68 (m, 1H), 2.95 (s, 3H), 2.28 – 2.06 (m, 2H), 2.07 – 1.97 (m, 1H), 1.86 – 1.72 (m, 1H), 1.72 – 1.34 (m, 4H). ^{13}C NMR (100 MHz, Chloroform-*d*) δ 142.4, 128.4, 127.8, 127.0, 78.7, 67.1, 49.4, 26.4, 24.4, 20.4, 19.6. IR (neat film, significant peaks): 2934, 2095, 1445, 1251, 1073, 698. MS (ESI): 254 (M + Na^+). HRMS (APCI): Calculated for $C_{13}H_{18}NO$ (M + H^+ - N_2): 204.1388; Found: 204.1381.
Trans-diastereomer (41 mg, 35%): ^{1}H NMR (400 MHz, Chloroform-*d*) δ 7.45 – 7.27 (m, 5H), 3.17 (s, 3H), 3.06 (dd, *J* = 11.7, 4.0 Hz, 1H), 2.21 – 2.01 (m, 3H), 2.00 – 1.80 (m, 3H), 1.70 – 1.36 (m, 2H). ^{13}C NMR (100 MHz, Chloroform-*d*) δ 141.7, 128.2, 127.4, 126.8, 80.4, 68.2, 49.9, 30.8, 27.1, 24.7, 20.6. IR (neat film, significant peaks): 2934, 2094, 1445, 1251, 1074, 699. MS (ESI): 254 (M + Na^+). HRMS (ESI): Calculated for $C_{13}H_{17}N_3ONa$ (M + Na^+): 254.1269; Found: 254.1270.

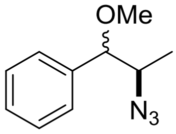

(2-azido-1-methoxypropyl)benzene (**3l**)

Prepared according to general procedure A reacting *β*-methylstyrene (324 μL). The title compound was obtained as a colourless oil (32 mg, *syn:anti* = 2.2:1, 34%). IR(neat): 2924, 2100, 1453, 1259, 1094, 702. MS (ESI): 214 (M + Na^+). HRMS (APCI): Calculated for $C_{10}H_{14}NO$ (M + H^+ - N_2): 164.1075; Found: 164.1084.
Syn-diastereomer (major): ^{1}H NMR (400 MHz, Chloroform-*d*) δ 7.44 – 7.27 (m, 5H), 4.18 (d, *J* = 4.9 Hz, 1H), 3.60 – 3.53 (m, 1H), 3.30 (s, 3H), 1.22 (d, *J* = 6.7 Hz, 3H). ^{13}C NMR (100 MHz, Chloroform-*d*) δ 138.0, 128.4, 128.4, 127.4, 86.3, 61.4, 57.2, 14.3. IR (neat film): 2924, 2104, 1453, 1259, 1095, 702.
Anti-diastereomer (minor): ^{1}H NMR (400 MHz, Chloroform-*d*) δ 7.44 – 7.28 (m, 5H), 4.03 (d, *J* = 7.5 Hz, 1H), 3.67 – 3.61 (m, 1H), 3.26 (s, 3H), 0.98 (d, *J* = 6.7 Hz, 3H). ^{13}C NMR (100 MHz, Chloroform-*d*) δ 138.3, 128.6, 128.2, 127.5, 87.9, 61.9, 57.0, 16.4.

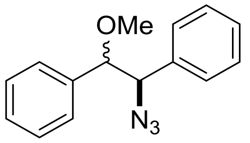

2-azido-1,2-diphenyl-1-methoxyethane (**3m**)

Prepared according to general procedure A reacting *cis*-stilbene (446 μL). The title compound was obtained as a colourless oil (101 mg, *syn:anti* = 1:1.5, 80%), contaminated with small amounts of the diazide **4a**. IR (neat film): 2928, 2099, 1453, 1254, 1101, 697. MS (EI): 225 (M - N_2, 52), 210 (M - HN_3, 85), 121 (M - $C_7H_6N_3$, 100). HRMS (APCI): Calculated for $C_{15}H_{15}O$ (M – N_3): 211.1123; Found: 211.1121.

Anti-diastereomer (major): ^{1}H NMR (400 MHz, Chloroform-*d*) δ 7.33 – 7.28 (m, 3H), 7.23 – 7.14 (m, 5H), 7.07 – 6.95 (m, 2H), 4.59 (d, *J* = 8.1 Hz, 1H), 4.31 (d, *J* = 8.1 Hz, 1H), 3.31 (s, 3H). ^{13}C NMR (100 MHz, Chloroform-*d*) δ 137.6, 136.3, 128.2, 128.2, 128.2, 128.0, 127.8, 127.6, 87.8, 70.9, 57.1.

Syn-diastereomer (minor): ^{1}H NMR (400 MHz, Chloroform-*d*) δ 7.33 – 7.28 (m, 3H), 7.23 – 7.14 (m, 5H), 7.07 – 6.95 (m, 2H), 4.71 (d, *J* = 6.3 Hz, 1H), 4.36 (d, *J* = 6.3 Hz, 1H), 3.19 (s, 3H). ^{13}C NMR (100 MHz, Chloroform-*d*) δ 137.3, 136.6, 128.4, 128.3, 128.2, 128.1, 128.0, 127.9, 86.4, 70.0, 57.2.

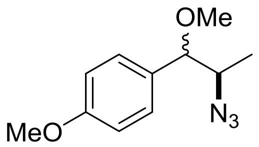

(2-azido-1-methoxypropyl)-4-methoxybenzene (**3n**)

Prepared according to general procedure A reacting anethole (375 μL). The title compound was obtained as a colourless oil (97 mg, *syn:anti* = 1:2.8, 88%). MS (APCI): 194 (M – N_2 + H^+). HRMS (APCI): Calculated for $C_{11}H_{16}NO_2$ (M – N_2 + H^+): 194.1141; Found: 194.1154.

Syn-diastereomer (minor): ^{1}H NMR (500 MHz, Chloroform-*d*) δ 7.23 (d, *J* = 8.6 Hz, 2H), 6.92 (d, *J* = 8.6 Hz, 2H), 4.10 (d, *J* = 5.1 Hz, 1H), 3.82 (s, 3H), 3.56 (dd, *J* = 6.7, 5.1 Hz, 1H), 3.26 (s, 3H), 1.21 (d, *J* = 6.7 Hz, 3H). ^{13}C NMR (125 MHz, Chloroform-*d*) δ 159.5, 129.9, 128.6, 113.8, 86.0, 61.5, 57.0, 55.3, 14.6. IR (neat film): 2910, 2095, 1511, 1096, 801

Anti-diastereomer (major): ^{1}H NMR (500 MHz, Chloroform-*d*) δ 7.20 (d, *J* = 8.7 Hz, 2H), 6.90 (d, *J* = 8.7 Hz, 2H), 3.97 (d, *J* = 7.6 Hz, 1H), 3.82 (s, 3H), 3.61 (dd, *J* = 7.6, 6.7 Hz, 1H), 3.23 (s, 3H), 0.96 (d, *J* = 6.7 Hz, 3H). ^{13}C NMR (125 MHz, Chloroform-*d*) δ 159.7, 130.3, 128.6, 113.9, 87.4, 62.0, 56.7, 55.3, 16.3. IR (neat film): 2934, 2085, 1511, 1086, 828.

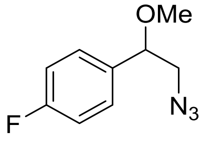

1-(2-azido-1-methoxyethyl)-4-fluorobenzene (**3o**)

Prepared according to general procedure A reacting 4-fluorostyrene (300 μL). The title compound was obtained as a colourless oil (49 mg, 50%), contaminated with small amounts of the diazide **4a**. ^{1}H NMR (500 MHz, Chloroform-*d*) δ 7.31 – 7.27 (m, 2H), 7.05 (t, *J* = 8.5 Hz, 2H), 4.34 (dd, *J* = 8.3, 3.8 Hz, 1H), 3.45 (dd, *J* = 12.9, 8.3 Hz, 1H), 3.30 (s, 3H), 3.19 (dd, *J* = 12.9, 3.8 Hz, 1H). ^{13}C NMR (125 MHz, Chloroform-*d*) δ 162.9 (d, *J* = 248.5 Hz), 134.3 (d, *J* = 3.2 Hz), 128.4 (d, *J* = 8.2 Hz), 115.7 (d, *J* = 21.5 Hz), 82.5, 56.9, 56.5. ^{19}F NMR (470 MHz, Chloroform-*d*) δ -114. IR (neat film): 2926, 2099, 1507, 1460, 1226, 1062, 834. IR (neat film): 2929, 2095, 1509, 1222, 1108, 833. MS (EI): 167 (M -

N_2, 2), 152 (M - HN_3, 2), 139 (M - CH_2N_3, 100). HRMS (APCI): Calculated for $C_9H_{11}NOF$ (M + H^+ - N_2): 168.0825; Found: 168.0839.

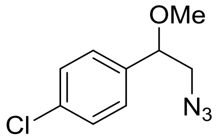

1-(2-azido-1-methoxyethyl)-4-chlorobenzene (**3p**)

Prepared according to general procedure A reacting 4-chlorostyrene (300 μL) The title compound was obtained as a colourless oil (71 mg, 67%). ^{1}H NMR (400 MHz, Chloroform-*d*) δ 7.36 (d, *J* = 8.2 Hz, 2H), 7.26 (d, *J* = 8.2 Hz, 2H), 4.34 (dd, *J* = 8.3, 3.7 Hz, 1H), 3.45 (dd, *J* = 12.9, 8.3 Hz, 1H), 3.30 (s, 3H), 3.19 (dd, *J* = 12.9, 3.7 Hz, 1H). ^{13}C NMR (100 MHz, Chloroform-*d*) δ 137.1, 134.2, 129.0, 128.0, 82.5, 57.0, 56.4. IR (neat film): 2929, 2098, 1438, 1114, 1097, 822. MS (EI): 185 ($^{37}ClM^+$-N_2, 3), 183 (^{35}ClM - N_2, 10), 166 (^{37}ClM - HN_3, 4), 164 (^{35}ClM - HN_3, 12), 157 (^{37}ClM - CH_2N_3, 34), 155 (^{35}ClM - CH_2N_3, 100). HRMS (APCI): Calculated for $C_9H_{11}NOCl$ (M + H^+ - N_2): 184.0529; Found: 184.0531.

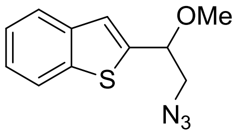

2-(2-azido-1-methoxyethyl)benzothiophene (**3q**)

Prepared according to general procedure A reacting 2-vinylbenzothiophene (400 mg). The title compound was obtained as a yellow oil (93 mg, 80%), contaminated with small amounts of the diazide **4a**. ^{1}H NMR (400 MHz, Chloroform-*d*) δ 7.88 – 7.81 (m, 1H), 7.80 – 7.72 (m, 1H), 7.39 – 7.31 (m, 2H), 7.28 (s, 1H), 4.69 (dd, *J* = 8.1, 3.9 Hz, 1H), 3.67 (dd, *J* = 12.8, 8.1 Hz, 1H), 3.42 (d, *J* = 1.0 Hz, 3H), 3.38 (dd, *J* = 13.0, 3.9 Hz, 1H). ^{13}C NMR (100 MHz, Chloroform-*d*) δ 142.8, 139.7, 139.2, 124.7, 124.5, 123.6, 122.9, 122.6, 79.4, 57.3, 56.3. IR (neat film): 2928, 2095, 1435, 1257, 1101, 772. MS (ESI): 256 (M + Na^+). HRMS (APCI): Calculated for $C_{10}H_9OS$ (M – CH_2N_3): 177.0374; Found: 177.0368.

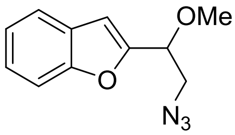

2-(2-azido-1-methoxyethyl)benzofuran (**3r**)

Prepared according to general procedure A reacting 2-vinylbenzofuran (360 mg). The title compound was obtained as a colourless oil (67 mg, 62%). ^{1}H NMR (400 MHz, Chloroform-*d*) δ 7.60 – 7.56 (m, 1H), 7.51 – 7.47 (m, 1H), 7.31 (td, *J* = 7.6, 1.5 Hz, 1H), 7.28 – 7.21 (m, 1H), 6.77 (s, 1H), 4.53 (dd, *J* = 8.0, 4.2 Hz, 1H), 3.76 (dd, *J* = 13.0, 8.0 Hz, 1H), 3.50 (dd, *J* = 13.0, 4.2 Hz, 1H), 3.44 (s, 3H). ^{13}C NMR (100 MHz, Chloroform-*d*) δ 155.1, 153.7, 127.7, 124.7, 123.0, 121.2, 111.5, 105.8, 76.8, 57.4, 53.6. IR (neat film): 2931, 2098, 1453, 1250, 1104, 809, 742. MS (EI): 217 (M, 1), 189 (M - N_2, 50), 161 (M - CH_2N_3, 100). HRMS (EI): Calculated for $C_{11}H_{11}N_3O_2$ (M^+): 217.0846; Found: 217.0838.

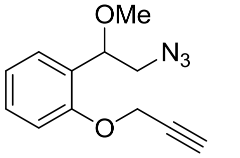

1-(2-azido-1-methoxyethyl)-2-(prop-2-yn-1-yloxy)benzene (**3s**)

Prepared according to general procedure A reacting 2-(prop-2-yn-1-yloxy)styrene (578 mg) in methanol (5 mL). The title compound was obtained as a colourless oil (50 mg, 42%). ^{1}H NMR (400 MHz, Chloroform-*d*) δ 7.43 (dd, *J* = 7.5, 1.8 Hz, 1H), 7.30 (ddd, *J* = 8.3, 7.5, 1.8 Hz, 1H), 7.06 (td, *J* = 7.5, 1.0 Hz, 1H), 7.00 (dd, *J* = 8.3, 1.0 Hz, 1H), 4.85 (dd, *J* = 7.6, 3.6 Hz, 1H), 4.74 (d, *J* = 2.4 Hz, 2H), 3.36 (s, 3H), 3.39 – 3.29 (m, 2H), 2.50 (t, *J* = 2.4 Hz, 1H). ^{13}C NMR (100 MHz, Chloroform-*d*) δ 154.8, 128.9, 127.2, 126.9, 121.8, 111.9, 78.3, 77.2, 75.7, 57.3, 55.9, 55.3. IR (neat film): 3290, 2929, 2099, 1601, 1487, 1220, 1103, 1023, 755. MS (EI): 231 (M, 10), 203 (M – N_2, 15), 175 (M – CH_2N_2, 100). HRMS (APCI): Calculated for $C_{12}H_{14}N_3O_2$ (M + H^+): 232.1086; Found: 232.1075.

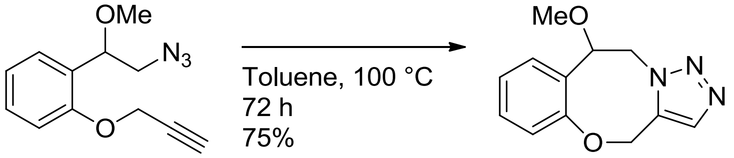

10-methoxy-10,11-dihydro-4H-benzo[*g*][1,2,3]triazolo[5,1-*c*][1,4]oxazocine (**7**)

Compound **3t** (20 mg, 0.09 mmol) was dissolved in toluene (5 mL) and was heated up to 100 °C, stirring under nitrogen, for 72 hours. The solvent was removed at reduced pressure and the crude was purified using preparative thin layer chromatography. The title compound was obtained as a yellow oil (15 mg, 75%). ^{1}H NMR (500 MHz, Chloroform-*d*) δ 7.33 (s, 1H), 7.30 (ddd, *J* = 8.0, 7.3, 1.8 Hz, 1H), 7.15 (td, *J* = 7.9, 1.5 Hz, 2H), 7.08 (td, *J* = 7.4, 1.3 Hz, 1H), 5.33 (dd, *J* = 14.3, 0.9 Hz, 1H), 5.26 (ddd, *J* = 13.9, 7.0, 0.5 Hz, 1H), 5.22 (d, *J* = 14.3 Hz, 1H), 5.04 (dd, *J* = 13.9, 8.3 Hz, 1H), 4.70 (dd, *J* = 8.3, 7.0 Hz, 1H), 3.37 (s, 3H). ^{13}C NMR (125 MHz, Chloroform-*d*) δ 155.8, 132.9, 132.5, 132.2, 130.8, 129.9, 125.3, 122.9, 81.2, 66.1, 56.9, 51.7. MS (ESI): 232 (M + H^+). HRMS (APCI): Calculated for $C_{12}H_{14}N_3O_2$ (M + H^+): 232.1086; Found: 232.1077.

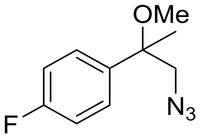

1-(1-azido-2-methoxypropan-2-yl)-4-fluorobenzene (example not in table)

Prepared according to general procedure A reacting α-methyl-4-fluorostyrene (337 μL). The title compound was obtained as a colourless oil (83 mg, 80%). ^{1}H NMR (400 MHz, Chloroform-*d*) δ 7.42 – 7.31 (m, 2H), 7.06 (t, *J* = 8.7 Hz, 2H), 3.40 (d, *J* = 12.5 Hz, 1H), 3.20 (d, *J* = 12.5 Hz, 1H), 3.12 (s, 3H), 1.63 (s, 3H). ^{13}C NMR (100 MHz, Chloroform-*d*) δ 162.3 (d, *J* = 246.5 Hz), 137.6 (d, *J* = 3.2 Hz), 128.2 (d, *J* = 8.1 Hz), 115.4 (d, *J* = 21.3 Hz), 79.2, 61.7, 50.5, 20.2. ^{19}F NMR (376 MHz, Chloroform-*d*) δ -115. IR (neat film): 2926, 2099, 1505, 1227, 1062, 834. MS (EI): 153 (M - CH_2N_3). HRMS (ESI): Calculated for $C_9H_{10}OF$ (M^+ -CH_2N_3): 153.0710; Found: 153.0706.

5 Characterisation data for azido-functionalised compounds 4a, 5 and 6

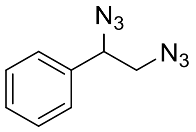

(1,2-diazidoethyl)benzene (**4a**)

Prepared according to general procedure C reacting styrene (90 μL) with sodium azide (325 mg) in acetonitrile (5 mL). The title compound was obtained as a colourless oil (90 mg, 96%). Data are in accordance with previous literature reports.[10] ^{1}H NMR (500 MHz, Chloroform-*d*) δ 7.45 – 7.36 (m, 3H), 7.36 – 7.31 (m, 2H), 4.67 (dd, *J* = 8.3, 4.9 Hz, 1H), 3.51 (dd, *J* = 12.7, 8.3 Hz, 1H), 3.45 (dd, *J* = 12.7, 4.9 Hz, 1H). ^{13}C NMR (125 MHz, Chloroform-*d*) δ 136.3, 129.1, 129.1, 126.9, 65.5, 56.0. IR (neat film): 2936, 2089, 1454, 1253, 758, 698.

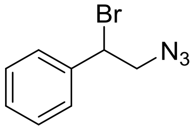

2-azido-1-bromo-1-phenylethane (**5**)

Prepared according to general procedure C reacting styrene (290 μL) with sodium bromide (515 mg) in acetonitrile (5 mL). The title compound was obtained as a colourless oil (52 mg, 46%). Data are in accordance with previous literature reports.[11] ^{1}H NMR (400 MHz, Chloroform-*d*) δ 7.48 – 7.32 (m, 5H), 5.01 (t, *J* = 7.3 Hz, 1H), 3.93 – 3.83 (m, 2H). ^{13}C NMR (100 MHz, Chloroform-*d*) δ 138.5, 129.3, 129.1, 127.7, 57.6, 51.2. IR (neat film): 3031, 2925, 2100, 1454, 1259, 695. MS (EI): 199 (^{81}BrM - N_2, 19), 197 (^{79}BrM – N_2, 20), 171 (^{81}BrM - CH_2N_3, 98), 169 (^{79}BrM – CH_2N_3, 100). HRMS (APCI): Calculated for $C_8H_9N^{79}Br$ (M + H^+ - N_2): 197.9918; Found: 197.9926.

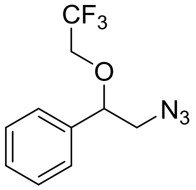

(2-azido-1-(2,2,2-trifluoroethoxy)ethyl)benzene (**6**)

Prepared according to general procedure B reacting styrene (290 μL) in trifluoroethanol (5 mL). The title compound was obtained as a colourless oil (60 mg, 49%). ^{1}H NMR (400 MHz, Chloroform-*d*) δ 7.43 – 7.37 (m, 3H), 7.37 – 7.30 (m, 2H), 4.64 (dd, *J* = 8.4, 3.6 Hz, 1H), 3.80 (dq, *J* = 12.0, 8.8 Hz, 1H), 3.72 (dq, *J* = 12.0, 8.4 Hz, 1H), 3.59 (dd, *J* = 13.2, 8.4 Hz, 1H), 3.23 (dd, *J* = 13.2, 3.6 Hz, 1H). ^{13}C NMR (100 MHz, Chloroform-*d*) δ 136.8, 129.2, 129.1, 126.7, 123.8 (q, *J* = 279 Hz), 83.1, 66.1 (q, *J* = 34 Hz), 56.4. ^{19}F NMR (376 MHz, Chloroform-*d*) δ -74(t, *J* = 8.6 Hz). IR (neat film): 2936, 2100, 1454, 1276, 1156, 1123, 960, 700. MS (EI): 217 (M – N_2, 2), 189 (M – CH_2N_2, 100). HRMS (EI): Calculated for $C_{10}H_{10}NOF_3$ (M – N_2): 217.0709; Found: 217.0709.

6 Characterisation data for diazido-functionalised compounds 4a – j and diamine 8

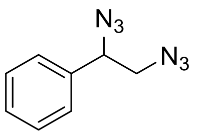

(1,2-diazidoethyl)benzene (**4a**)

Prepared according to general procedure D reacting styrene (290 μL) in methanol (5 mL). The title compound was obtained as a colourless oil (45 mg, 95%). Data are in accordance with previous literature reports.[10] ^{1}H NMR (400 MHz, Chloroform-*d*) δ 7.45 – 7.36 (m, 3H), 7.36 – 7.31 (m, 2H), 4.67 (dd, *J* = 8.3, 4.9 Hz, 1H), 3.51 (dd, *J* = 12.7, 8.3 Hz, 1H), 3.45 (dd, *J* = 12.7, 4.9 Hz, 1H). ^{13}C NMR (100 MHz, Chloroform-*d*) δ 136.3, 129.1, 129.1, 126.9, 65.5, 56.0. IR (neat film): 2936, 2089, 1454, 1253, 758, 698.

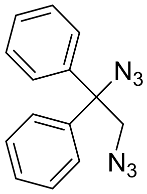

(1,2-diazidoethyl)-1,1-dibenzene (**4b**)

Prepared according to general procedure D reacting 1,1-diphenylethene (441 μL) in methanol (5 mL). The title compound was obtained as a colourless oil (47 mg, 72%). ^{1}H NMR (400 MHz, Chloroform-*d*) δ 7.45 – 7.27 (m, 10H), 4.03 (s, 2H). ^{13}C NMR (100 MHz, Chloroform-*d*) δ 140.1, 128.7, 128.4, 127.4, 72.5, 59.4. IR (neat film): 2929, 2091, 1446, 1248, 756, 660. MS (APCI): 209 (M + H^+ - N_4). HRMS (APCI): Calculated for $C_{14}H_{13}N_2$ (M + H^+ - N_4): 209.1079; Found: 209.1087.

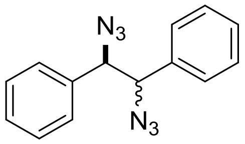

1,2-diazido-1,2-diphenylethane (**4c**)

Prepared according to general procedure D reacting *cis*-stilbene (446 μL) in methanol (5 mL). The title compound was obtained as a colourless oil (25 mg, 38% of a 1:1.2 *syn:anti* mixture). Data are in accordance with previous literature reports.[12] IR (neat film): 2093, 1453, 1243, 762, 698.

Syn Product: ^{1}H NMR (400 MHz, Chloroform-*d*) δ 7.39 (dd, *J* = 5.2, 1.9 Hz, 4H), 7.31 – 7.20 (m, 4H), 7.10 – 7.03 (m, 2H), 4.64 (s, 2H). ^{13}C NMR (100 MHz, Chloroform-*d*) δ 135.7, 128.7, 128.6, 127.7, 70.7.

Anti Product: ^{1}H NMR (400 MHz, Chloroform-*d*) δ 7.39 (dd, *J* = 5.2, 1.9 Hz, 4H), 7.31 – 7.20 (m, 4H), 7.10 – 7.03 (m, 2H), 4.69 (s, 2H). ^{13}C NMR (100 MHz, Chloroform-*d*) δ 135.8, 129.0, 128.7, 128.0, 69.6.

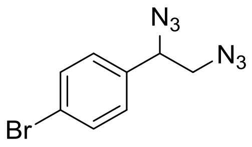

1-(1,2-diazidoethyl)-4-bromobenzene (**4d**)

Prepared according to general procedure D reacting 4-bromostyrene (327 μL) in methanol (5 mL). The title compound was obtained as a colourless oil (65 mg, 97%). Data are in accordance with previous literature reports.[13] ^{1}H NMR (400 MHz, Chloroform-*d*) δ 7.55 (d, *J* = 8.4 Hz, 2H), 7.22 (d, *J* = 8.4 Hz, 2H), 4.64 (dd, *J* = 8.0, 5.0 Hz, 1H), 3.48 (dd, *J* = 12.8, 8.0 Hz, 1H), 3.42 (dd, *J* = 12.8, 5.0 Hz, 1H). ^{13}C NMR (100 MHz, Chloroform-*d*) δ 135.4, 132.3, 128.6, 123.1, 64.9, 55.8. IR (neat film): 2917, 2092, 1489, 1250, 1010, 819. MS (EI): 211 (^{81}BrM – N_4, 19), 209 (^{79}BrM – N_4, 20), 184 (^{81}BrM – N_2 - CH_2N_3, 98), 183 (^{79}BrM – N_2 - CH_2N_3, 100), 157 (^{81}BrM – $C_2H_3N_6$, 49), 155 (^{79}BrM –$C_2H_3N_6$, 50).

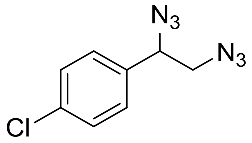

1-(1,2-diazidoethyl)-4-chlorobenzene (**4e**)

Prepared according to general procedure D reacting 4-chlorostyrene (300 μL) in methanol (5 mL). The title compound was obtained as a colourless oil (38 mg, 68%). Data are in accordance with previous literature reports.[13] ^{1}H NMR (400 MHz, Chloroform-*d*) δ 7.39 (d, *J* = 8.5 Hz, 2H), 7.28 (d, *J* = 8.5 Hz, 2H), 4.66 (dd, *J* = 8.0, 5.0 Hz, 1H), 3.48 (dd, *J* = 12.8, 8.0 Hz, 1H), 3.42 (dd, *J* = 12.8, 5.0 Hz, 1H). ^{13}C NMR (100 MHz, Chloroform-*d*) δ 135.0, 134.9, 129.4, 128.3, 64.8, 55.9. IR (neat film): 2926, 2090, 1492, 1331, 1250, 1091, 824. MS (EI): 139 (^{37}ClM – N_2 – CH_2N_3, 35), 137 (^{35}ClM – N_2 – CH_2N_3, 100).

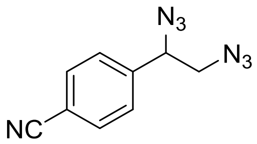

1-(1,2-diazidoethyl)-4-cyanobenzene (**4f**)

Prepared according to general procedure D reacting 4-cyanostyrene (323 μL) in methanol (5 mL). The title compound was obtained as a colourless oil (26 mg, 49%). ^{1}H NMR (400 MHz, Chloroform-*d*) δ 7.72 (d, *J* = 8.2 Hz, 1H), 7.47 (d, *J* = 8.2 Hz, 2H), 4.74 (dd, *J* = 7.3, 5.4 Hz, 1H), 3.45 – 3.55 (m, 2H). ^{13}C NMR (125 MHz, Chloroform-*d*) δ 141.6, 132.9, 127.8, 118.2, 113.0, 64.8, 55.8. IR (neat film): 2927, 2229, 2093, 1253, 834.

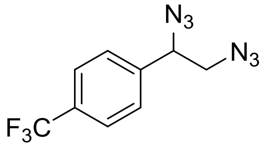

1-(1,2-diazidoethyl)-4-trifluorobenzene (**4g**)

Prepared according to general procedure D reacting 4-trifluoromethylstyrene (369 μL) in methanol (5 mL). The title compound was obtained as a colourless oil (34 mg, 53%). ^{1}H NMR (400 MHz, Chloroform-*d*) δ 7.61 (d, *J* = 8.1 Hz, 2H), 7.40 (d, *J* = 8.1 Hz, 2H), 4.67 (dd, *J* = 7.9, 5.0 Hz, 1H), 3.49 – 3.33 (m, 2H). ^{13}C NMR (100 MHz, Chloroform-*d*) δ 140.4, 131.2 (q, *J* = 32.7 Hz), 130.72, 126.1 (q, *J* = 3.8 Hz), 123.8 (q, *J* = 272.5 Hz), 64.9, 55.9. ^{19}F NMR (376 MHz, Chloroform-*d*) δ -62. IR (neat film): 2095, 1323, 1164, 1113, 1067, 839. MS (EI): 200 (M – N_4, 10), 172 (M – CH_2N_4, 100), 145 (M – $C_2H_3N_6$, 75). HRMS (APCI): Calculated for $C_9H_8N_2F_3$ (M + H^+ - N_4): 201.0640; Found: 201.0634.

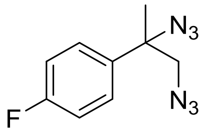

1-(1,2-diazidopropan-2-yl)-4-fluorobenzene (**4h**)

Prepared according to general procedure D reacting 4-fluoro-α-methylstyrene (337 μL) in methanol (5 mL). The title compound was obtained as a colourless oil (44 mg, 80%). ^{1}H NMR (400 MHz, Chloroform-*d*) δ 7.42 (dd, *J* = 8.9, 5.2 Hz, 2H), 7.09 (dd, *J* = 8.9, 8.3 Hz, 2H), 3.46 (d, *J* = 12.5 Hz, 1H), 3.39 (d, *J* = 12.5 Hz, 1H), 1.76 (s, 3H). ^{13}C NMR (125 MHz, Chloroform-*d*) δ 162.4 (d, *J* = 247.8 Hz), 136.4 (d, *J* = 3.4 Hz), 127.7 (d, *J* = 8.2 Hz), 115.8 (d, *J* = 21.5 Hz), 66.1, 61.0, 22.4. ^{19}F NMR (376 MHz, Chloroform-*d*) δ -113.7. IR (neat film): 2935, 2097, 1509, 1232, 1164, 833. MS (EI): 136 (M – CH_2N_3 – N_2).

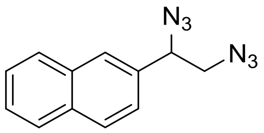

2-(1,2-diazidoethyl)-naphthalene (**4i**)

Prepared according to general procedure D reacting 2-vinylnaphthalene (385 mg) in methanol (5 mL). The title compound was obtained as a colourless oil (57 mg, 92%). Data are in accordance with previous literature reports.[13] ^{1}H NMR (400 MHz, Chloroform-*d*) δ 7.94 – 7.80 (m, 4H), 7.57 – 7.50 (m, 2H), 7.43 (dd, *J* = 8.5, 1.9 Hz, 1H), 4.85 (dd, *J* = 8.4, 4.9 Hz, 1H), 3.60 (dd, *J* = 12.8, 8.4 Hz, 1H), 3.52 (dd, *J* = 12.8, 4.9 Hz, 1H). ^{13}C NMR (100 MHz, Chloroform-*d*) δ 133.6, 133.4, 133.1, 129.2, 128.1, 127.8, 126.8, 126.8, 126.6, 124.0, 65.8, 55.9. IR (neat film): 3056, 2927, 2092, 1252, 817, 746. MS (EI): 154 (M – CH_2N_3).

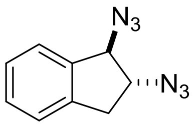

1,2-diazido-2,3-dihydro-1*H*-indene (**4j**)

Prepared according to general procedure D reacting indene (292 μL) in methanol (5 mL). The title compound was obtained as a colourless oil (38 mg, 76% of *trans*-isomer; 6 mg, 12% of *cis*-isomer). Data are in accordance with previous literature reports.[14]

Trans-diastereomer: ^{1}H NMR (400 MHz, Chloroform-*d*) δ 7.41 – 7.36 (m, 1H), 7.36 – 7.29 (m, 2H), 7.26 (q, *J* = 3.1, 2.6 Hz, 1H), 4.77 (d, *J* = 5.6 Hz, 1H), 4.23 – 4.10 (m, 1H), 3.35 (dd, *J* = 16.0, 7.3 Hz, 1H), 2.94 (dd, *J* = 16.0, 6.6 Hz, 1H). ^{13}C NMR (100 MHz, Chloroform-*d*) δ 139.1, 137.7, 129.5, 127.8,

125.2, 124.6, 70.3, 67.7, 36.2. IR (neat film): 2920, 2091, 1317, 1253, 747. MS (EI): 129 (M – HN_3 – N_2)

Cis-diastereomer: ^{1}H NMR (400 MHz, Chloroform-*d*) δ 7.40 – 7.25 (m, 4H), 4.84 (d, *J* = 5.6 Hz, 1H), 4.30 (q, *J* = 6.4 Hz, 1H), 3.22 – 3.10 (m, 2H). ^{13}C NMR (100 MHz, Chloroform-*d*) δ 139.7, 137.5, 129.8, 127.7, 125.4, 125.0, 66.9, 64.1, 35.6. MS (EI): 129 (M – HN_3 – N_2)

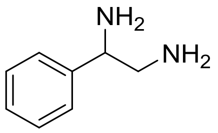

(1,2-diaminoethyl)benzene (**8**)

(1,2-diazidoethyl)benzene (90 mg, 0.48 mmol) was dissolved in ethanol (mL) and subjected to hydrogenation using the H-cube (Pd/C cartridge, 1 mL/min flow rate). Complete conversion was achieved and the title compound was obtained as a yellow solid (66 mg, 0.48 mmol, 100%). Data are in accordance with previous literature reports.[15] ^{1}H NMR (400 MHz, Chloroform-*d*) δ 7.40 – 7.30 (m, 5H), 3.96 (dd, *J* = 7.4, 5.2 Hz, 1H), 2.96 (dd, *J* = 12.8, 5.2 Hz, 1H), 2.85 (dd, *J* = 12.8, 7.4 Hz, 1H). ^{13}C NMR (100 MHz, Chloroform-*d*) δ 143.7, 128.6, 127.4, 126.5, 57.7, 49.3. IR (neat film): 3287, 3029, 1637, 1549, 1478, 1317, 1288, 696. MS (ESI): 137 (M + H^+).

7 ^{1}H NMR, ^{13}C NMR and IR spectra for methoxyazidated compounds 3a – t and oxazocine 7

Data for (2-azido-1-methoxyethyl)benzene (**3a**)

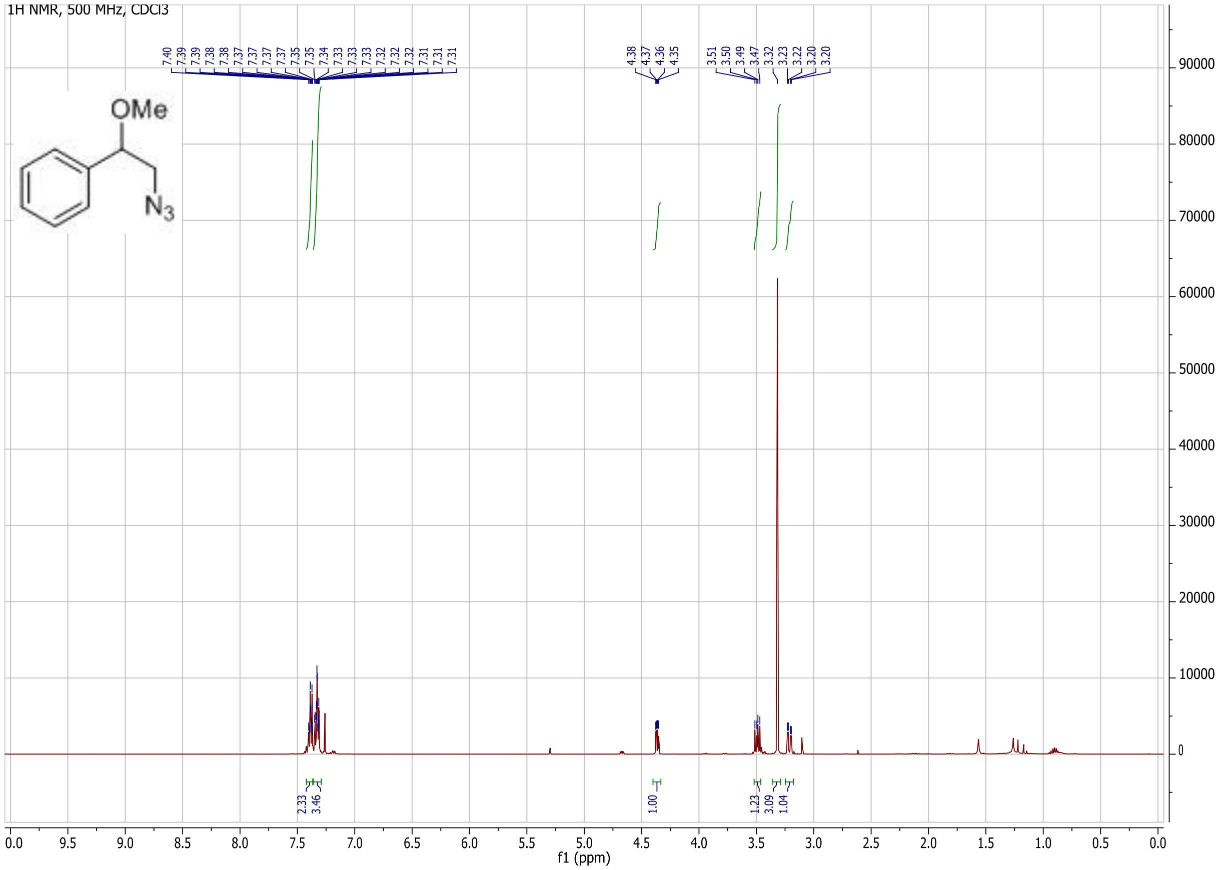

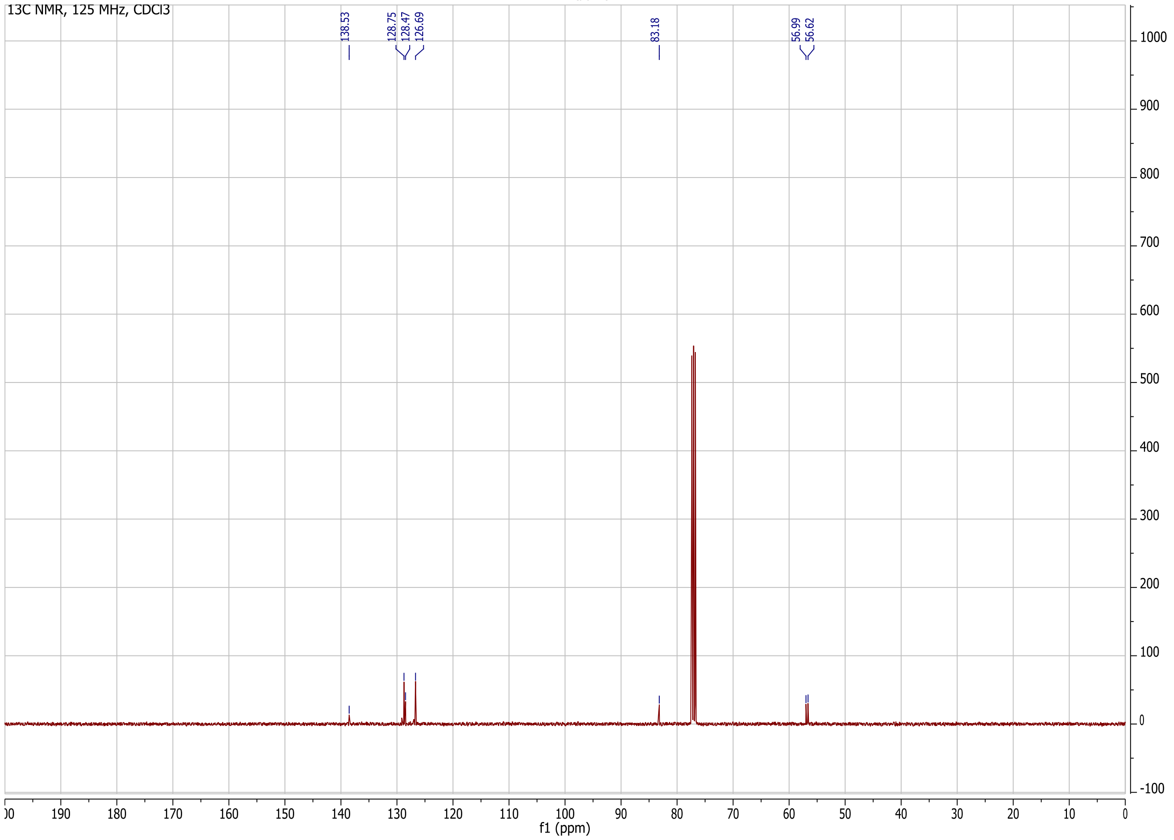

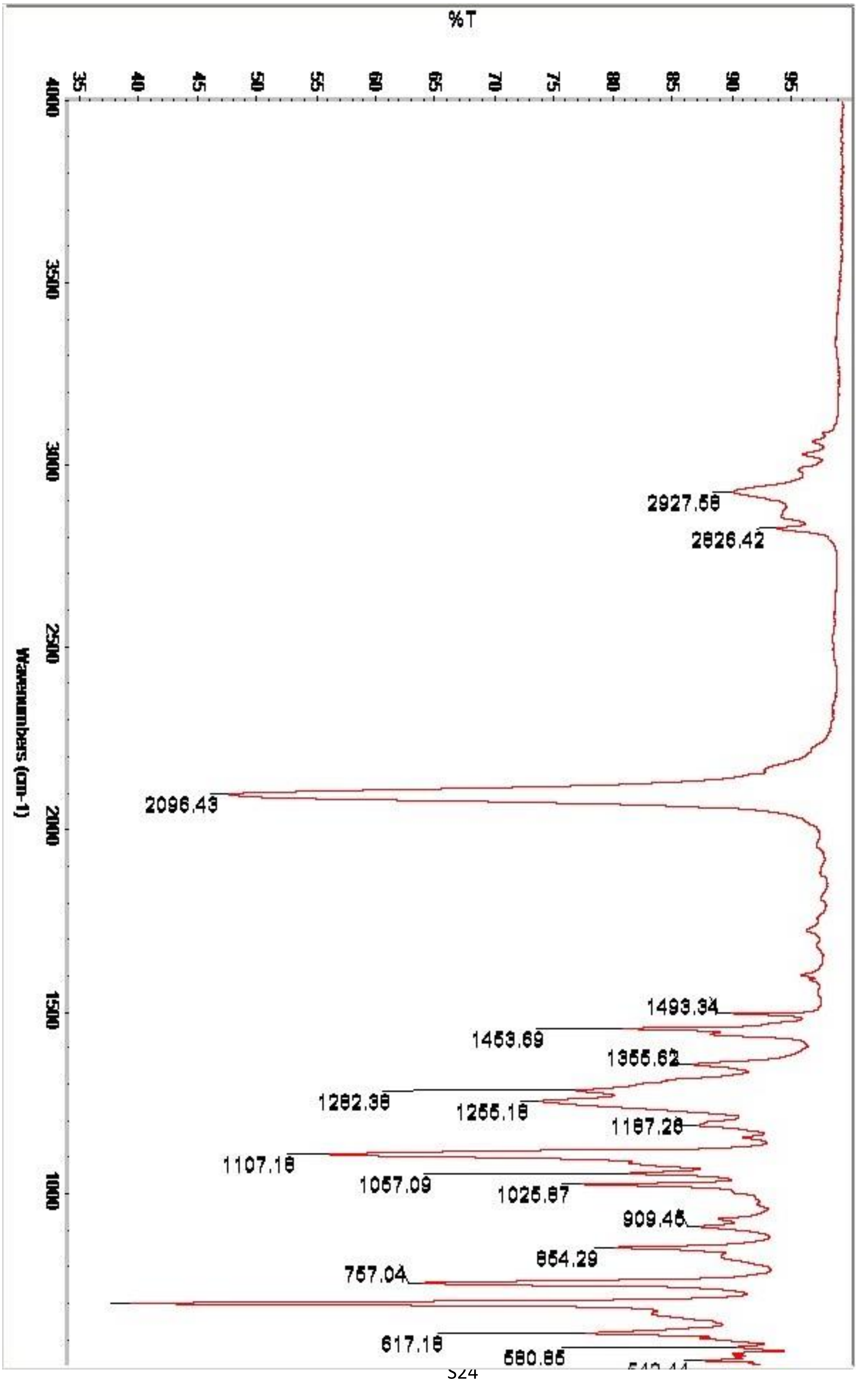
%T
Wavenumbers (cm-1)

Data for 1-(2-azido-1-methoxyethyl)-2-methylbenzene (**3b**)

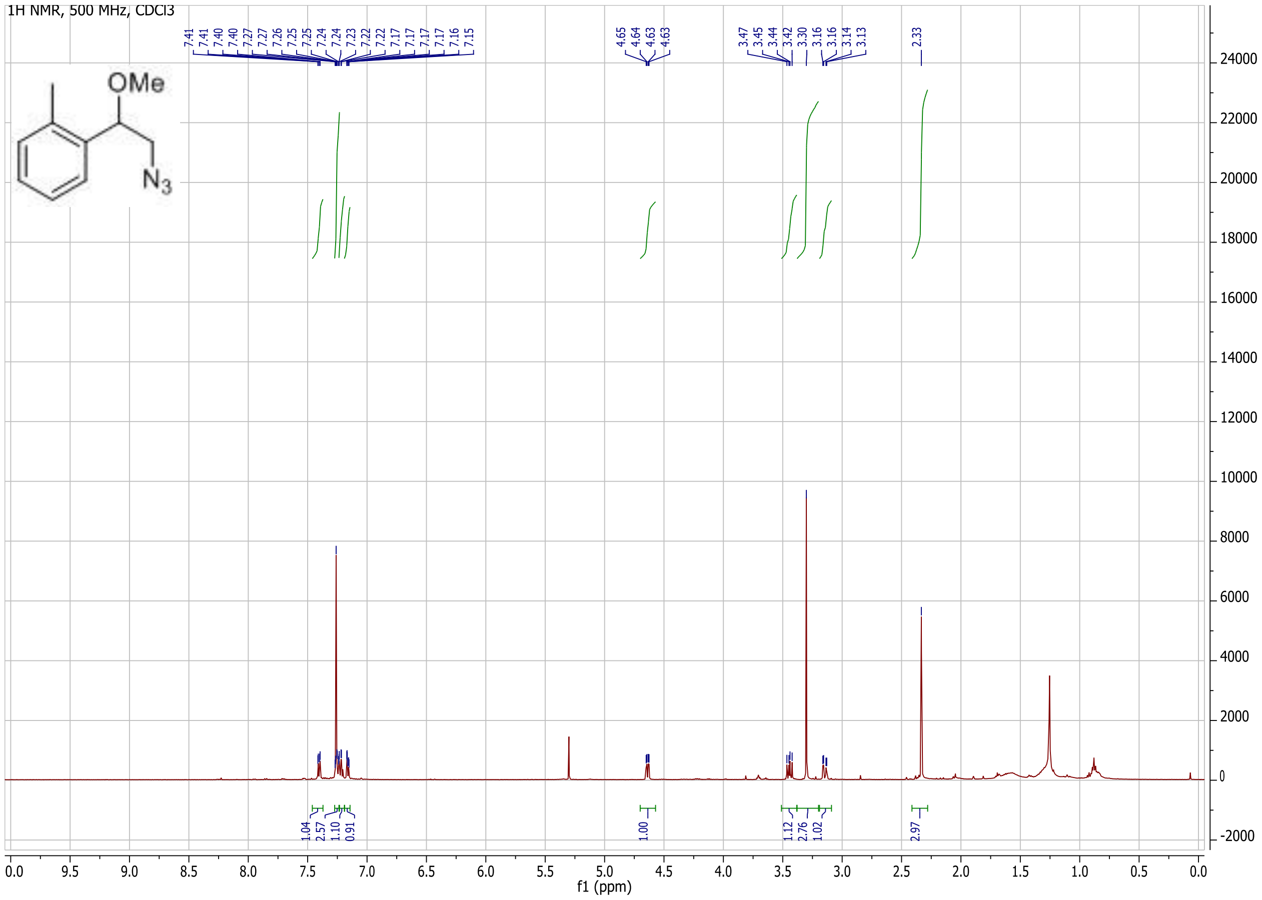

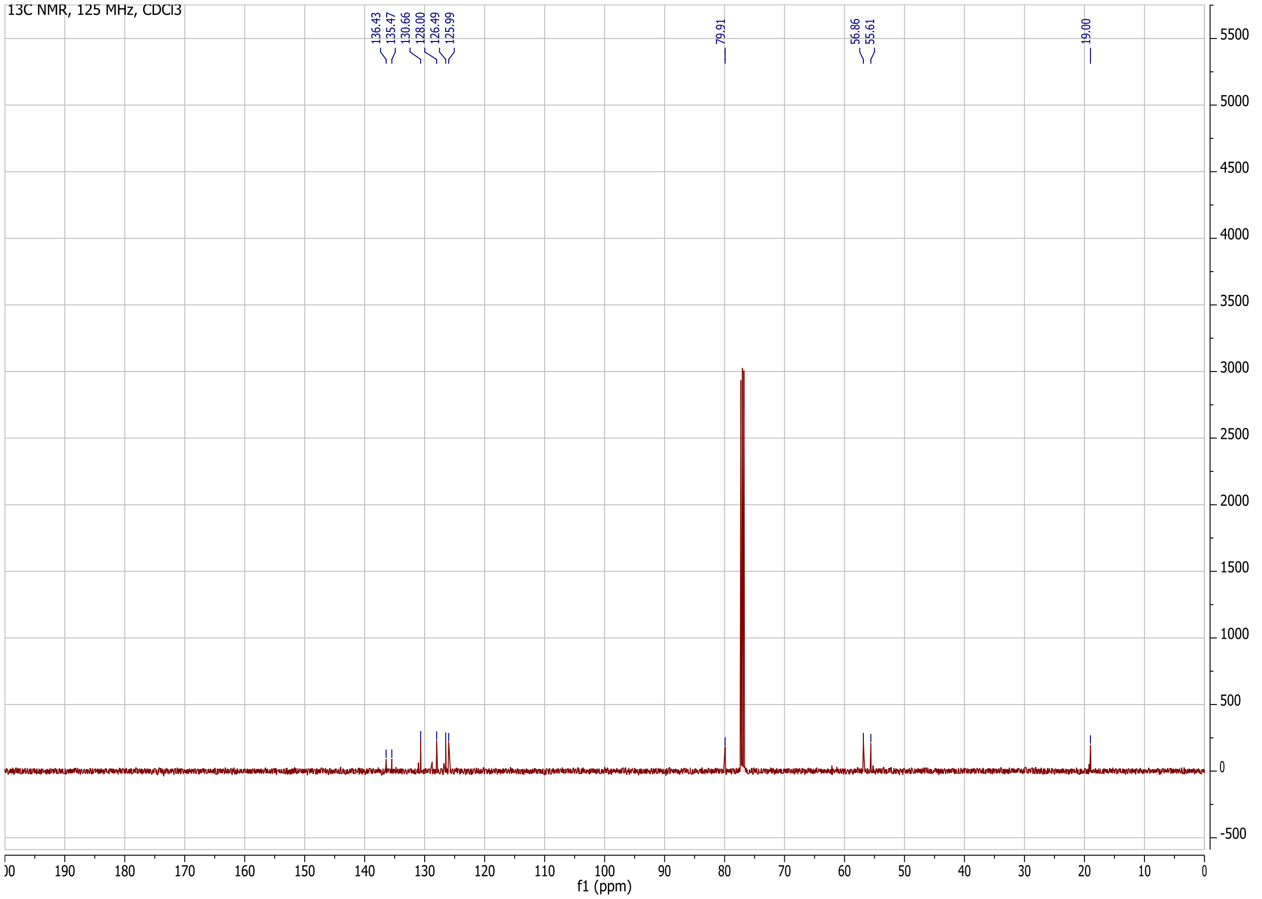

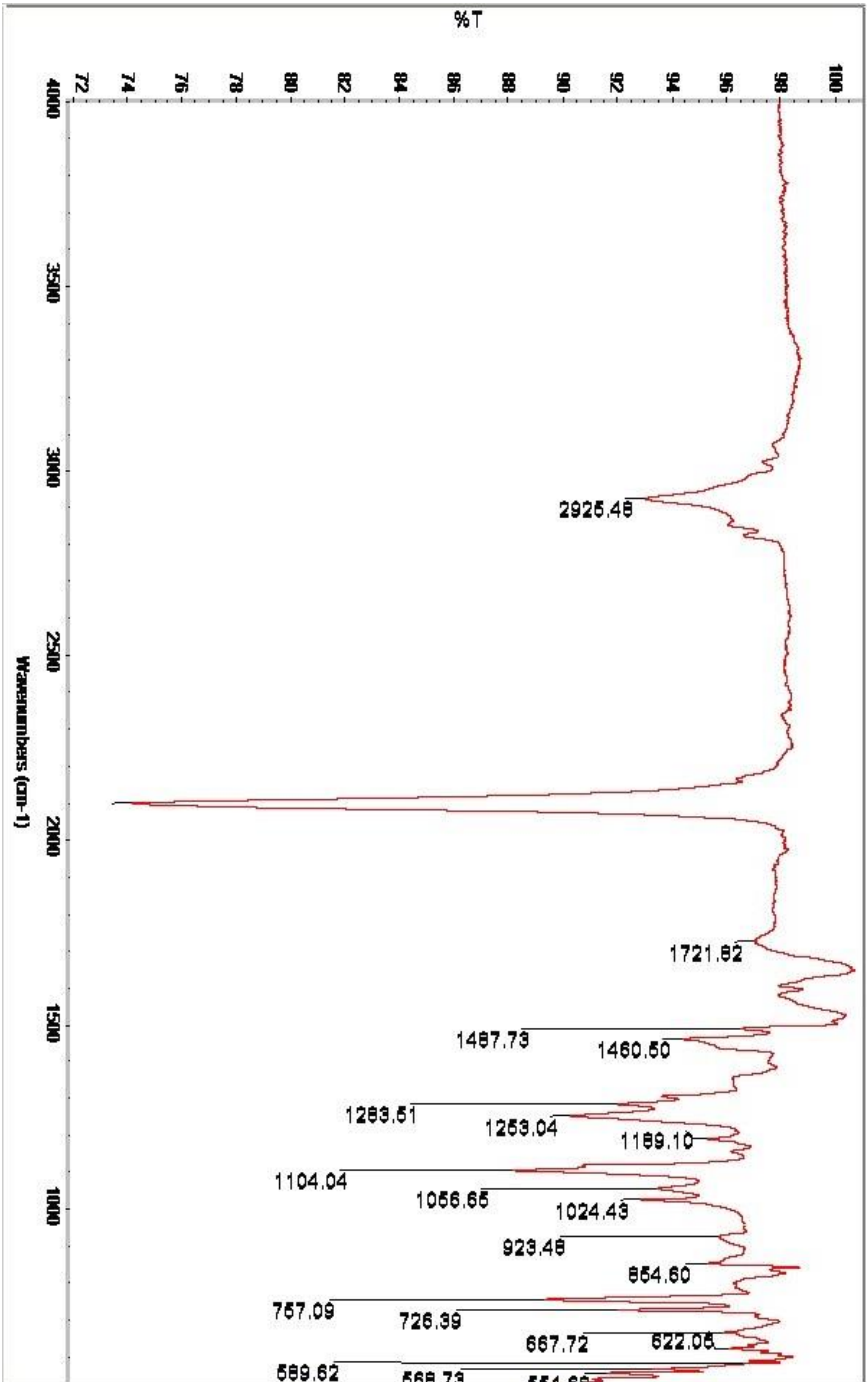
%T
Wavenumbers (cm-1)
2925.46
1721.82
1487.73
1460.50
1283.51
1253.04
1169.10
1104.04
1056.65
1024.43
923.46
854.60
757.09
726.39
667.72
622.06
589.62
568.73

Data for 1-(2-azido-1-methoxyethyl)-3-methylbenzene (**3c**)

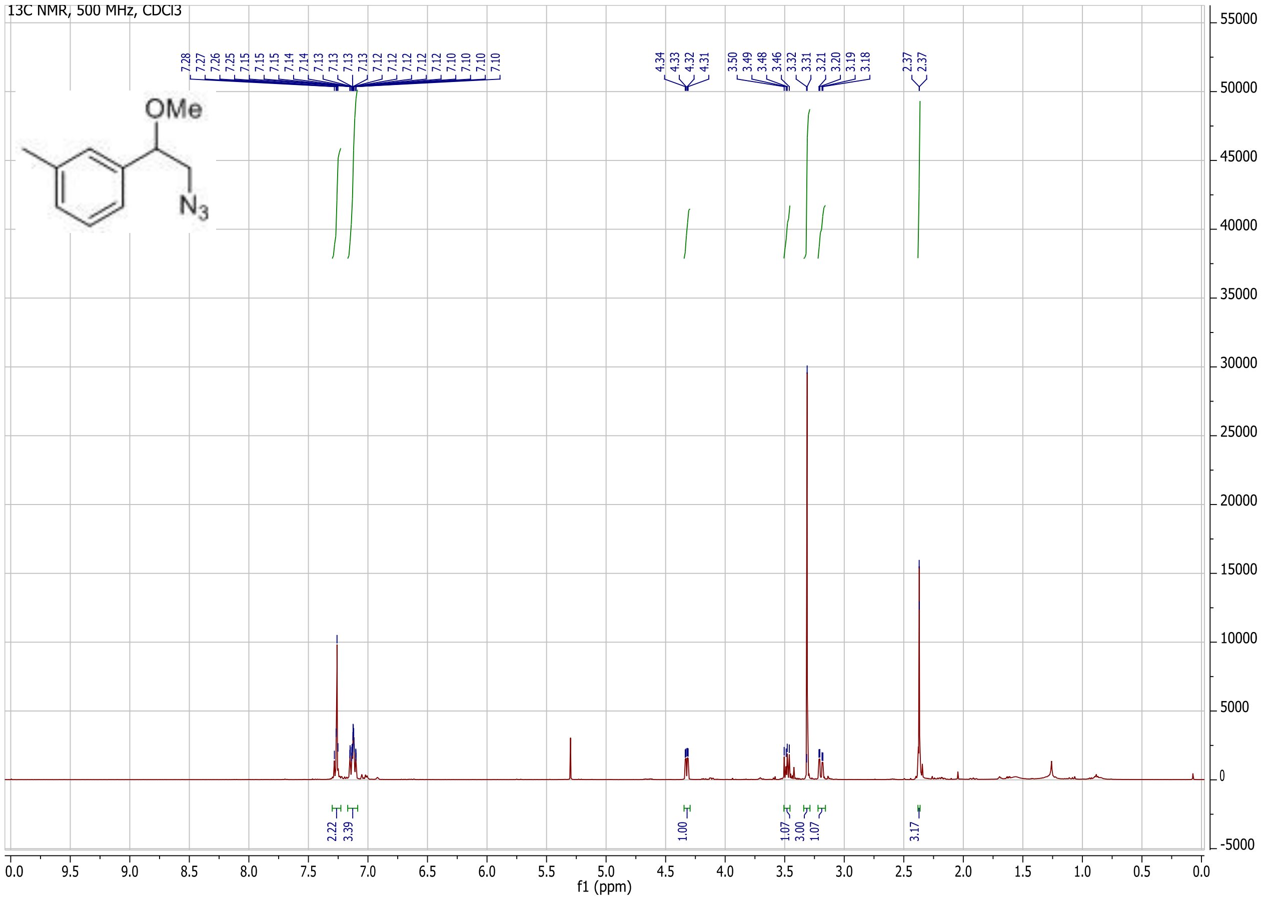

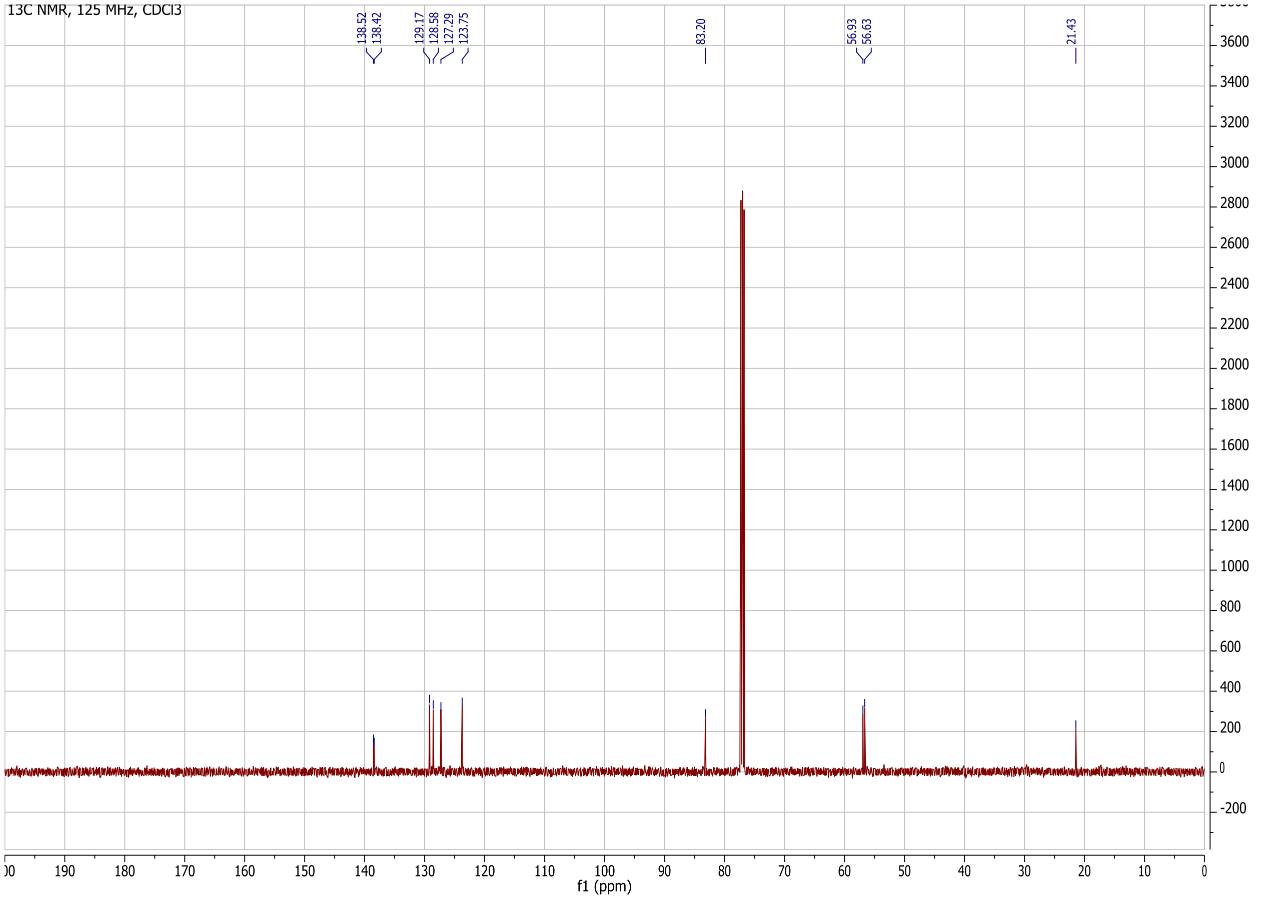

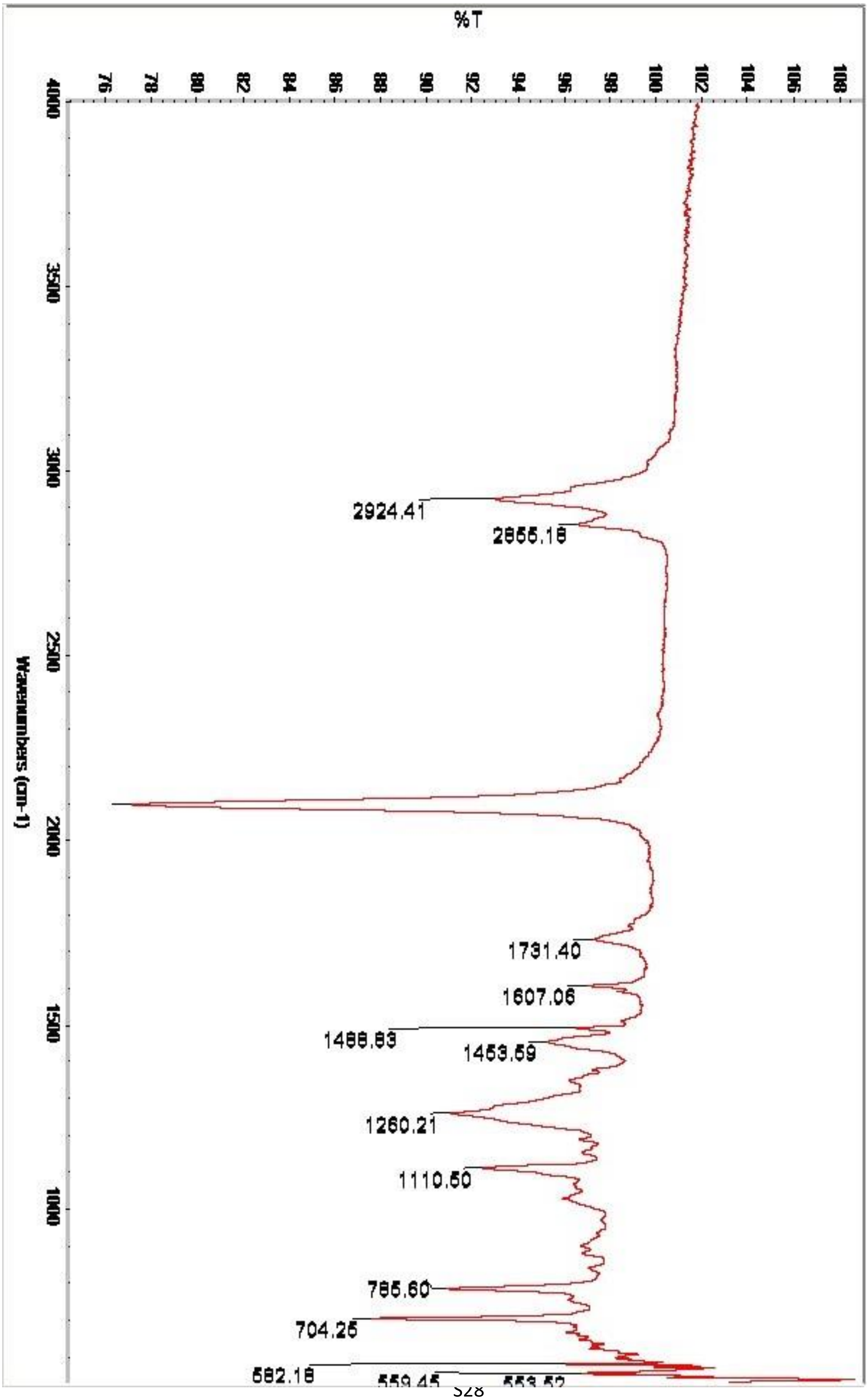

%T
76
78
80
82
84
86
88
90
92
94
96
98
100
102
104
106
108
4000
3500
3000
2500
2000
1500
1000
Wavenumbers (cm-1)
2924.41
2855.18
1731.40
1607.06
1488.83
1453.59
1260.21
1110.50
785.60
704.25
582.16
559.45
553.52

Data of 1-(2-azido-1-methoxyethyl)-4-methylbenzene (**3d**)

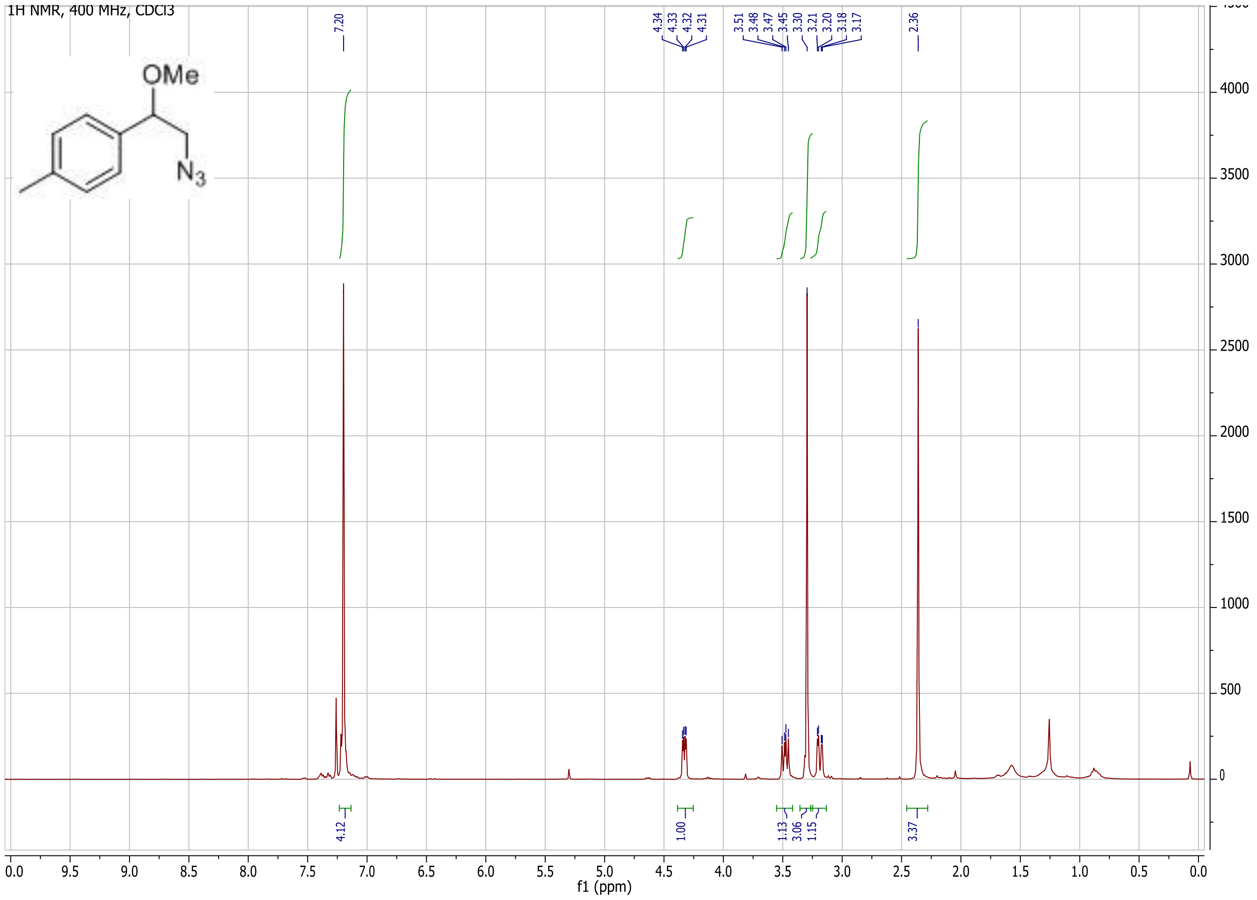

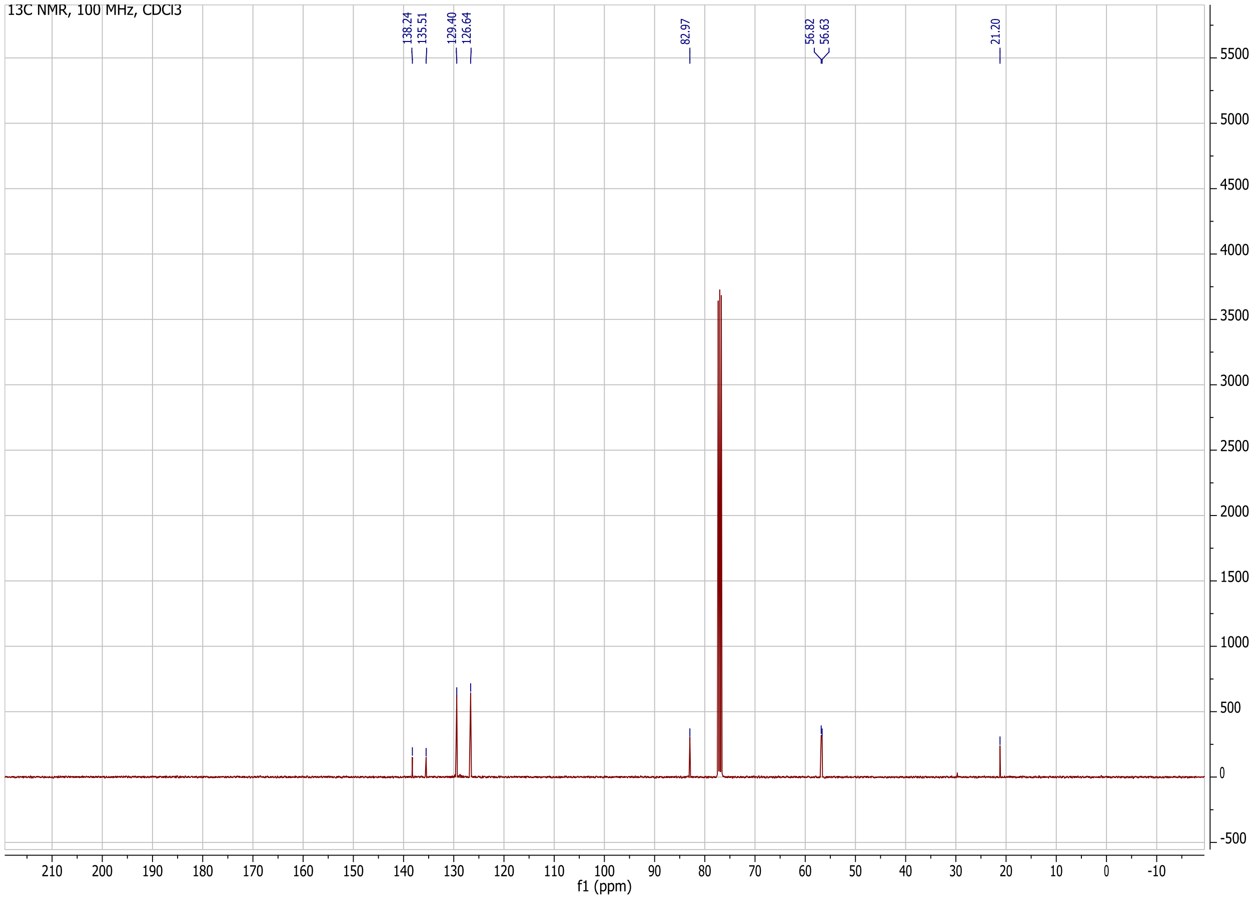

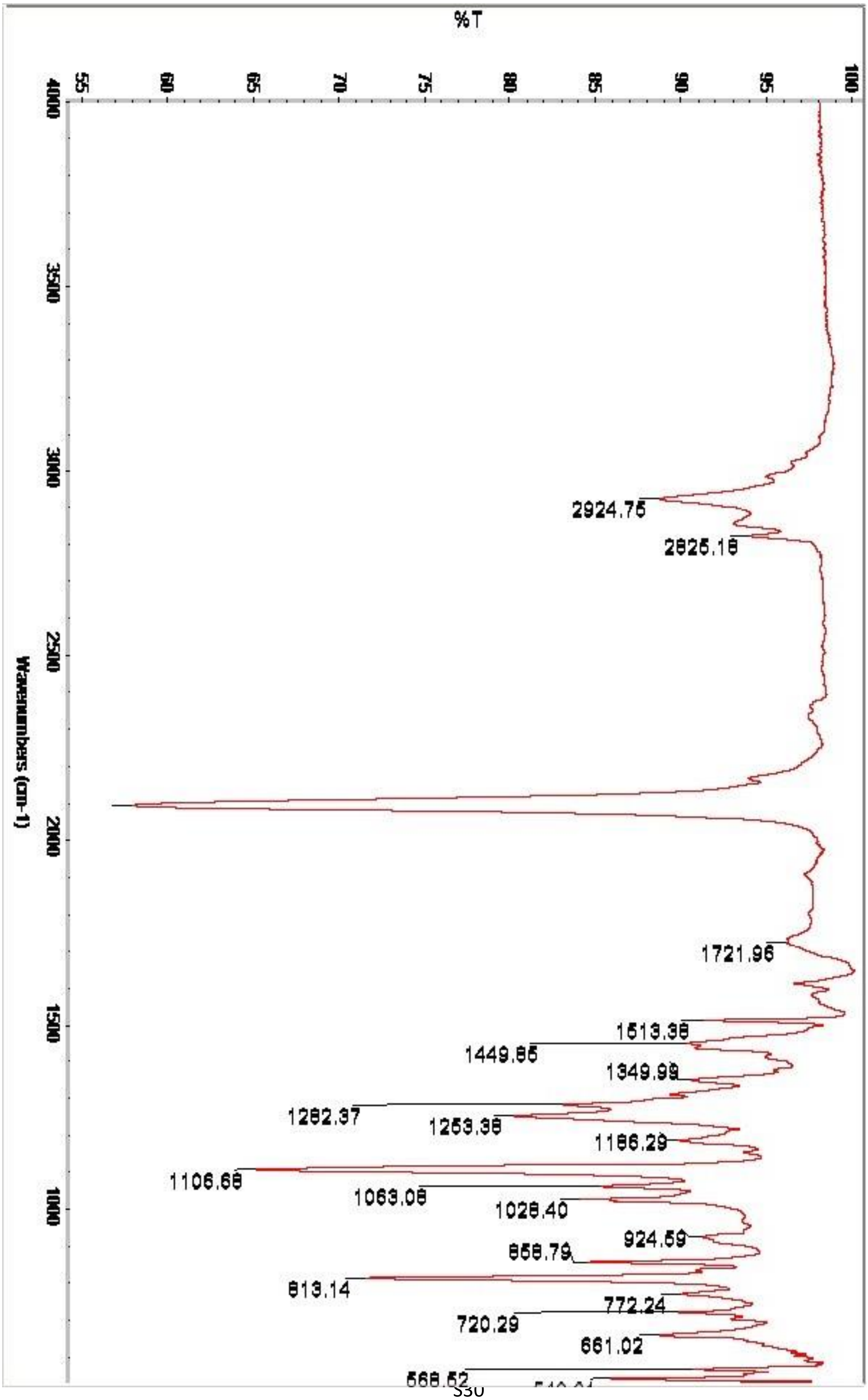
%T
55
60
65
70
75
80
85
90
95
100
4000
3500
3000
2500
2000
1500
1000
Wavenumbers (cm-1)
2924.75
2825.16
1721.96
1513.38
1449.85
1349.99
1282.37
1253.38
1186.29
1106.68
1063.08
1028.40
924.59
858.79
813.14
772.24
720.29
661.02

Data for 1-(2-azido-1-methoxyethyl)-2,4,6-trimethylbenzene (**3e**)

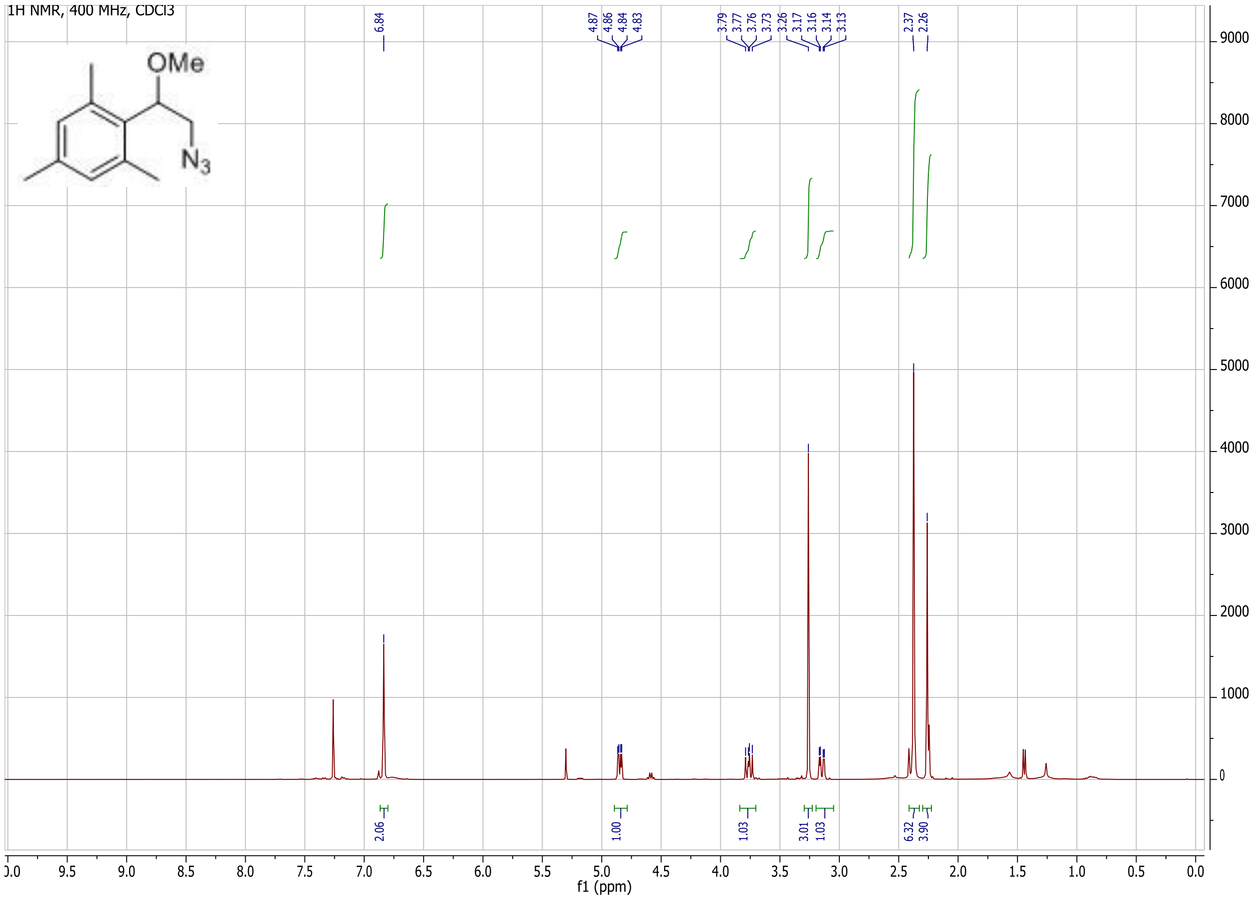

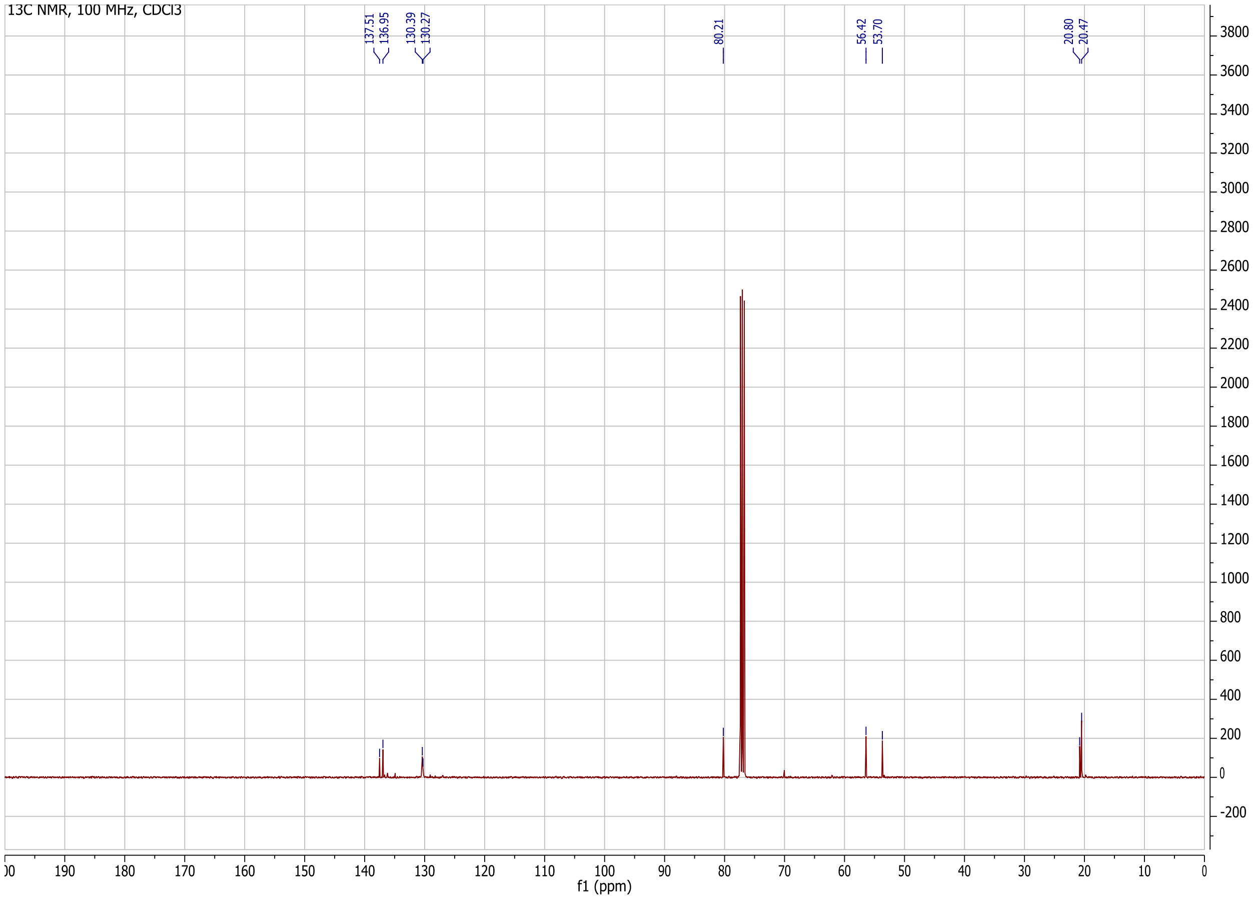

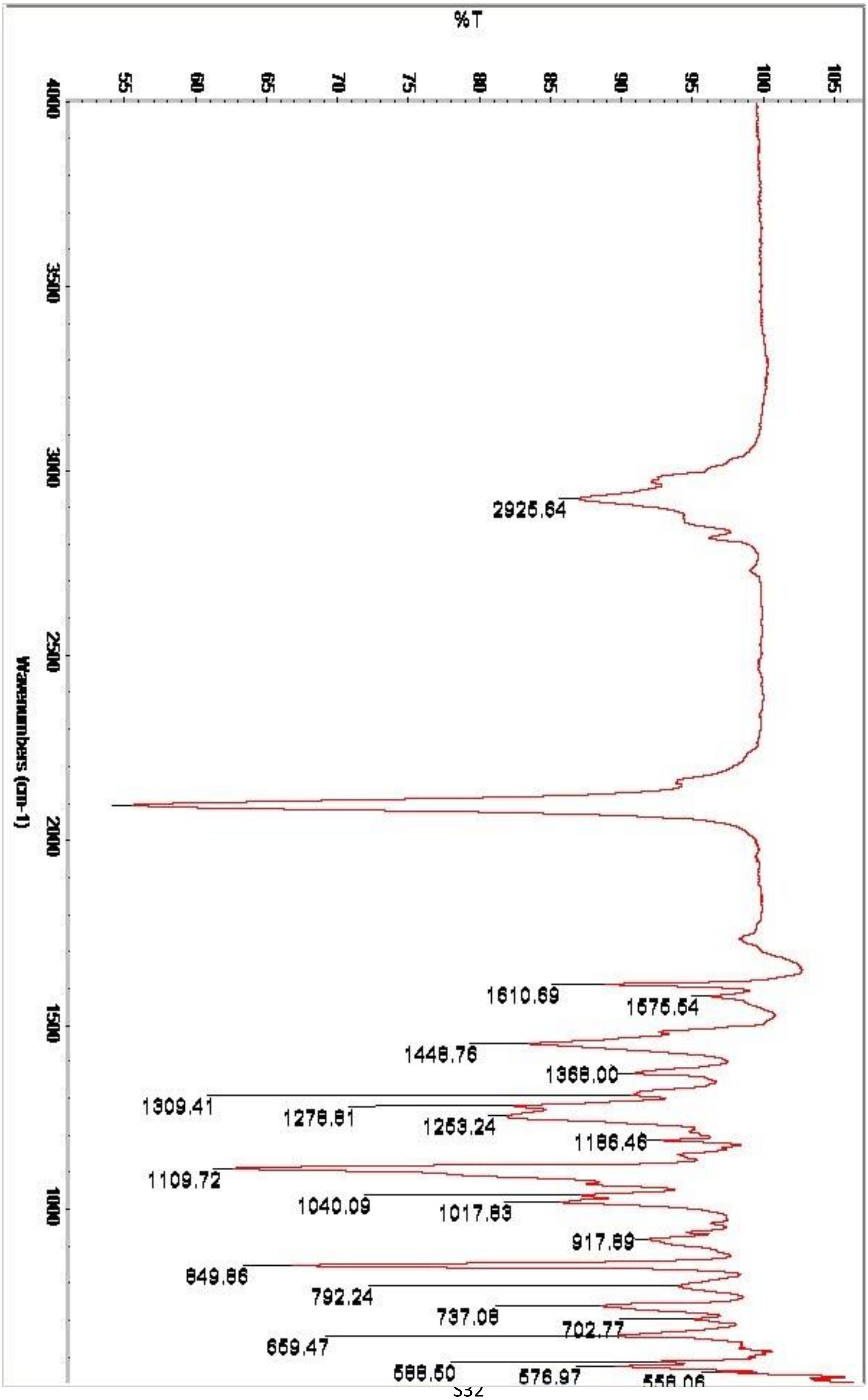
%T
55
60
65
70
75
80
85
90
95
100
105
4000
3500
3000
2500
2000
1500
1000
Wavenumbers (cm-1)
2925.64
1610.69
1575.54
1446.76
1368.00
1309.41
1276.81
1253.24
1186.46
1109.72
1040.09
1017.83
917.89
849.86
792.24
737.08
702.77
659.47
588.50
576.97
556.06

Data for 2-(2-azido-1-methoxyethyl)naphthalene (**3f**)

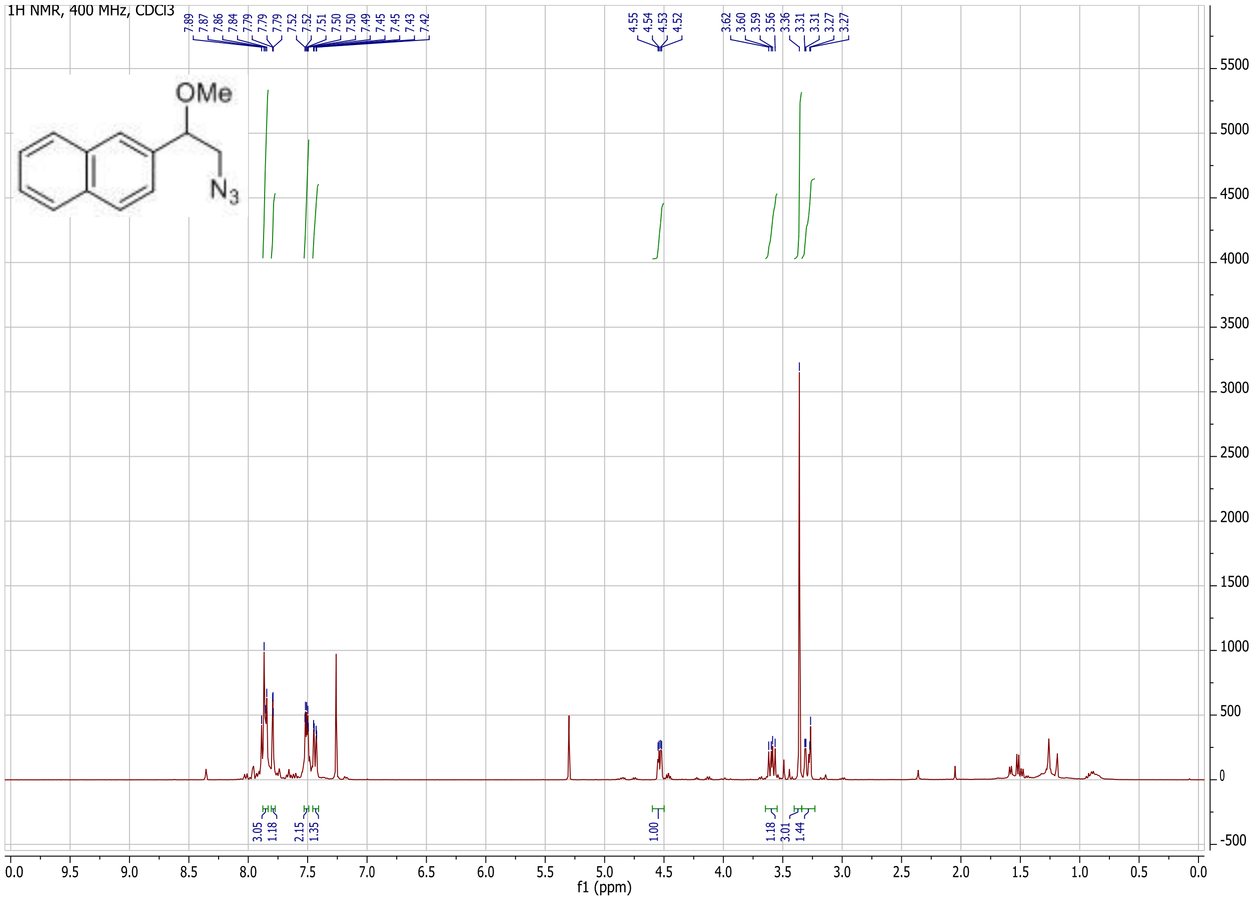

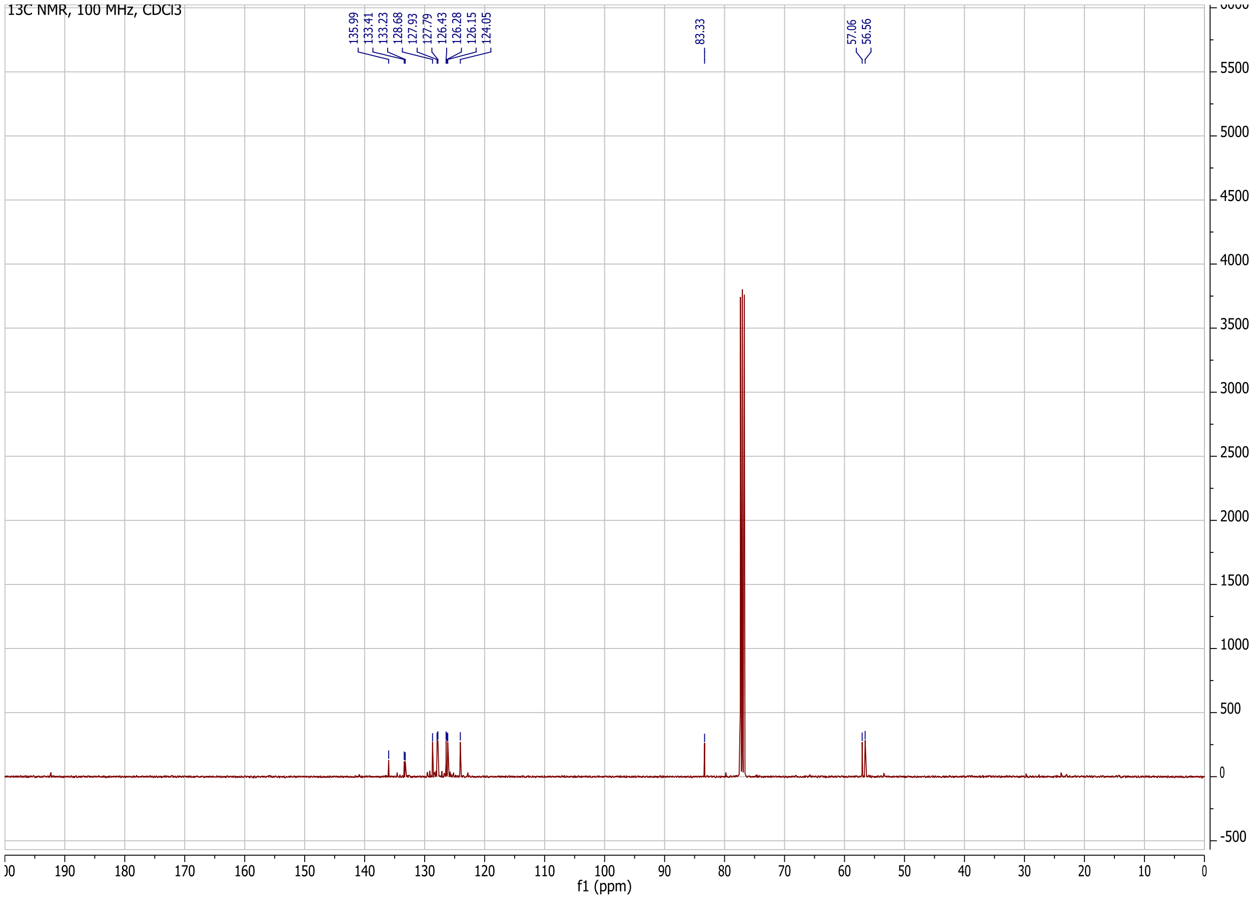

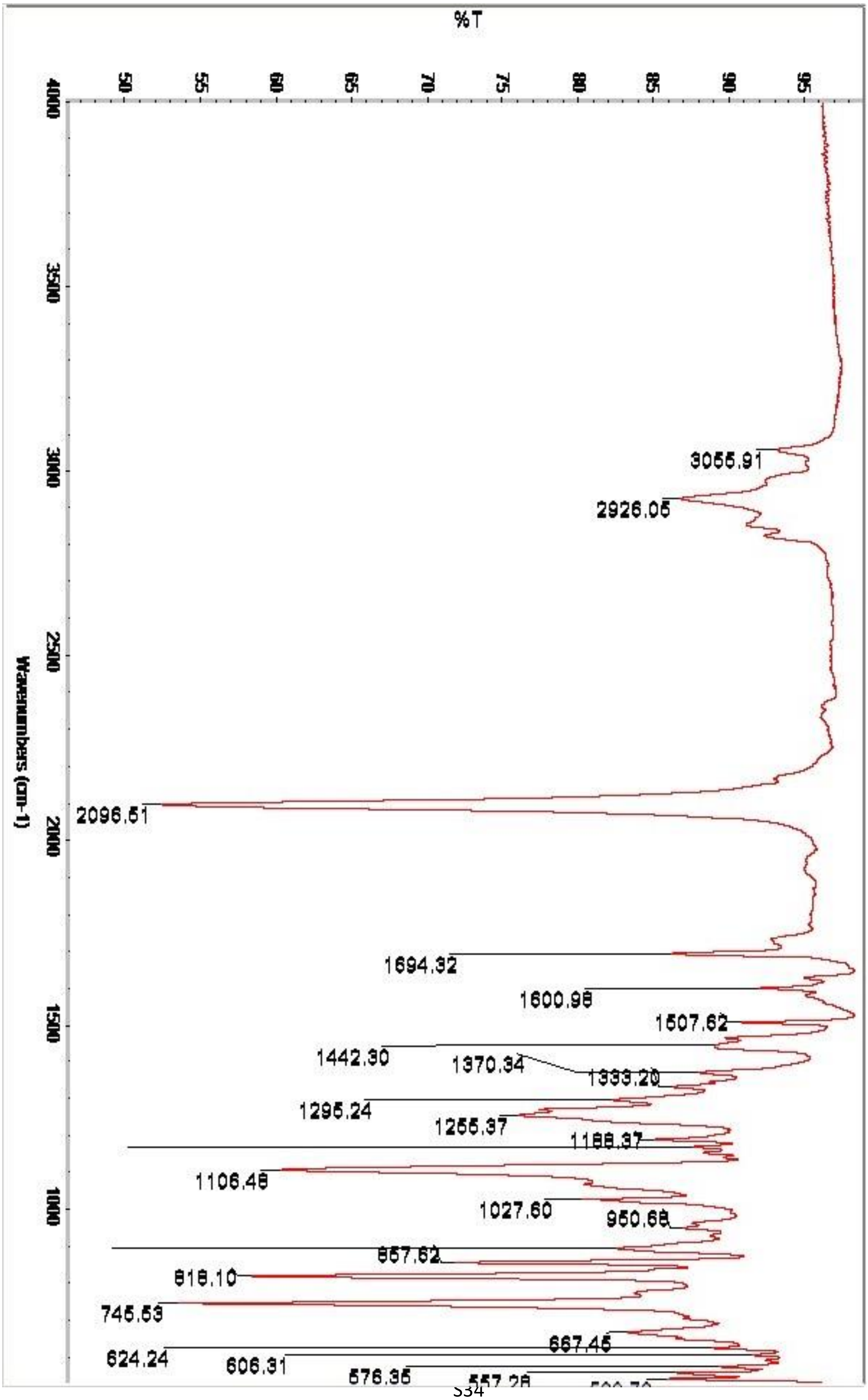
%T
Wavenumbers (cm-1)
3055.91
2926.05
2096.51
1694.32
1600.98
1507.62
1442.30
1370.34
1333.20
1295.24
1255.37
1186.37
1106.48
1027.60
950.68
867.62
816.10
745.53
667.45
624.24
606.31
576.35

Data for 2-azido-1-methoxy-2,3-dihydro-1*H*-indene (**3g**)

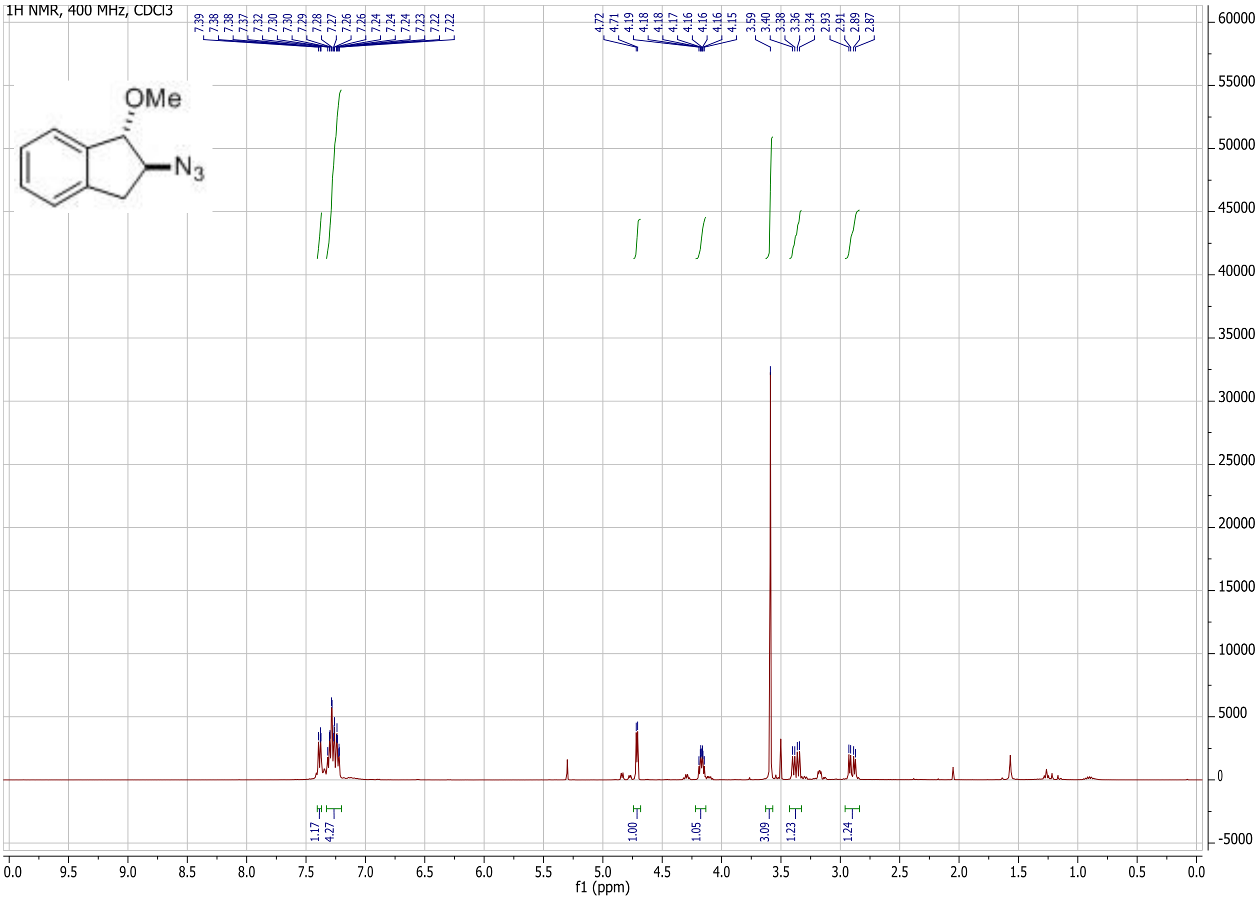

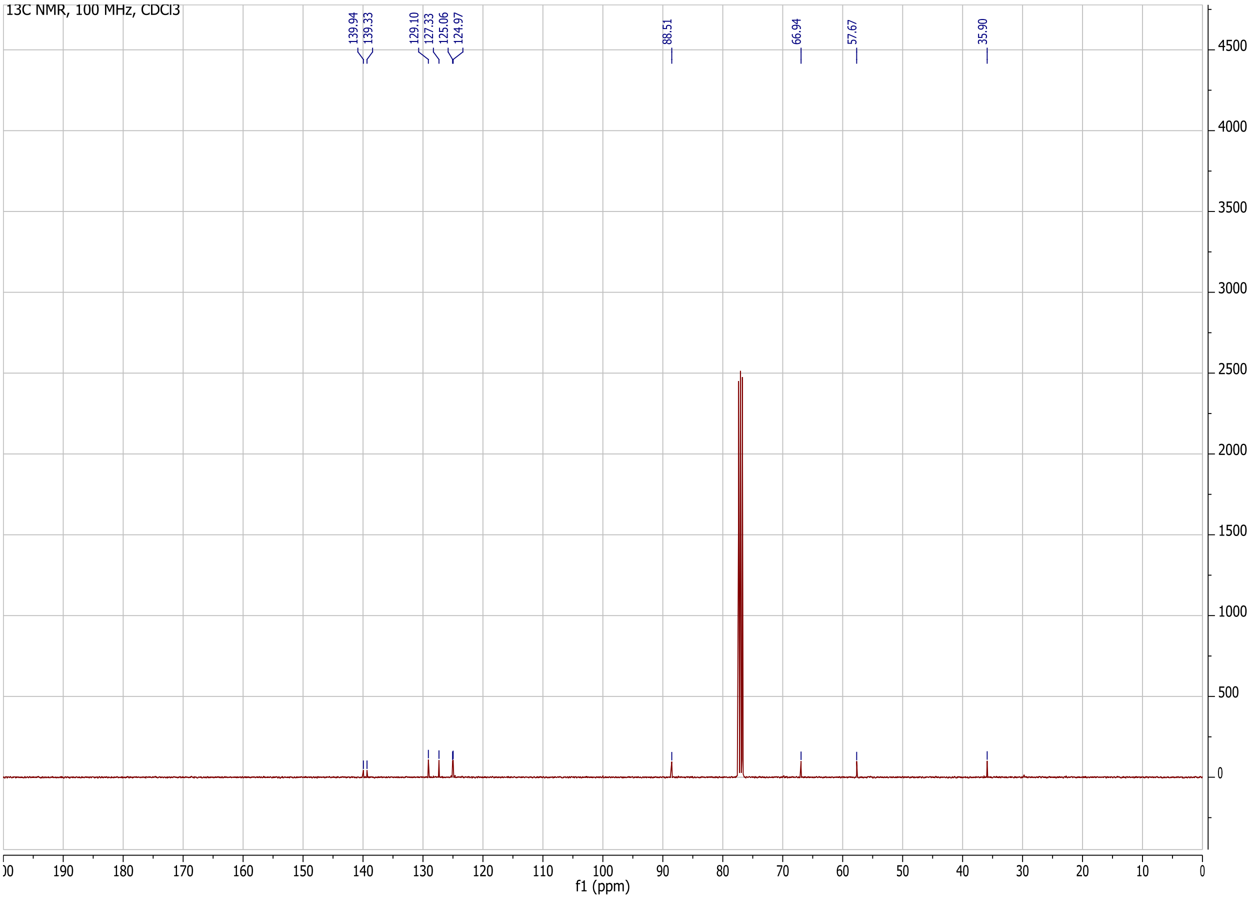

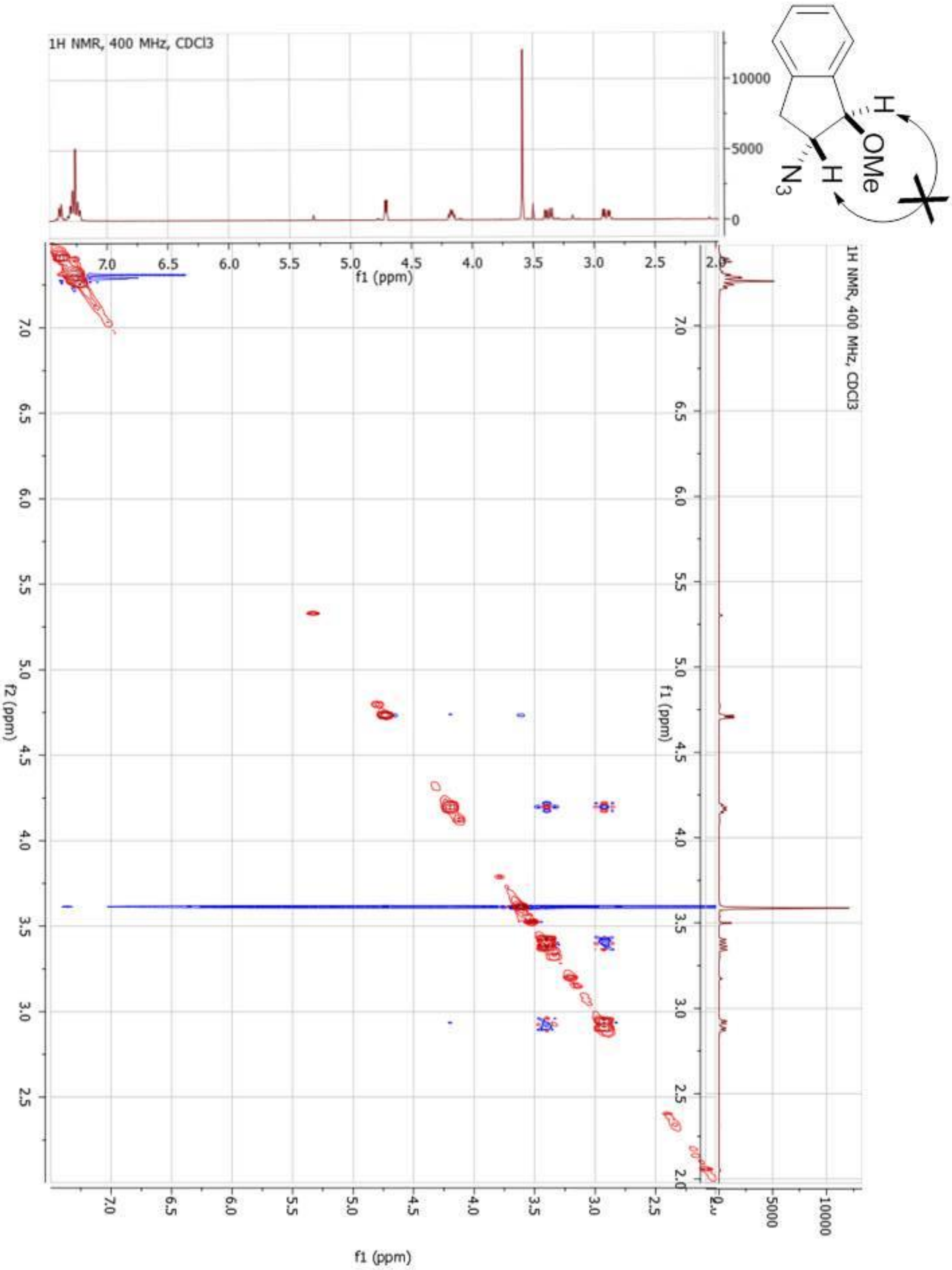
1H NMR, 400 MHz, CDCl3
f1 (ppm)
f2 (ppm)
N3
H
OMe
H

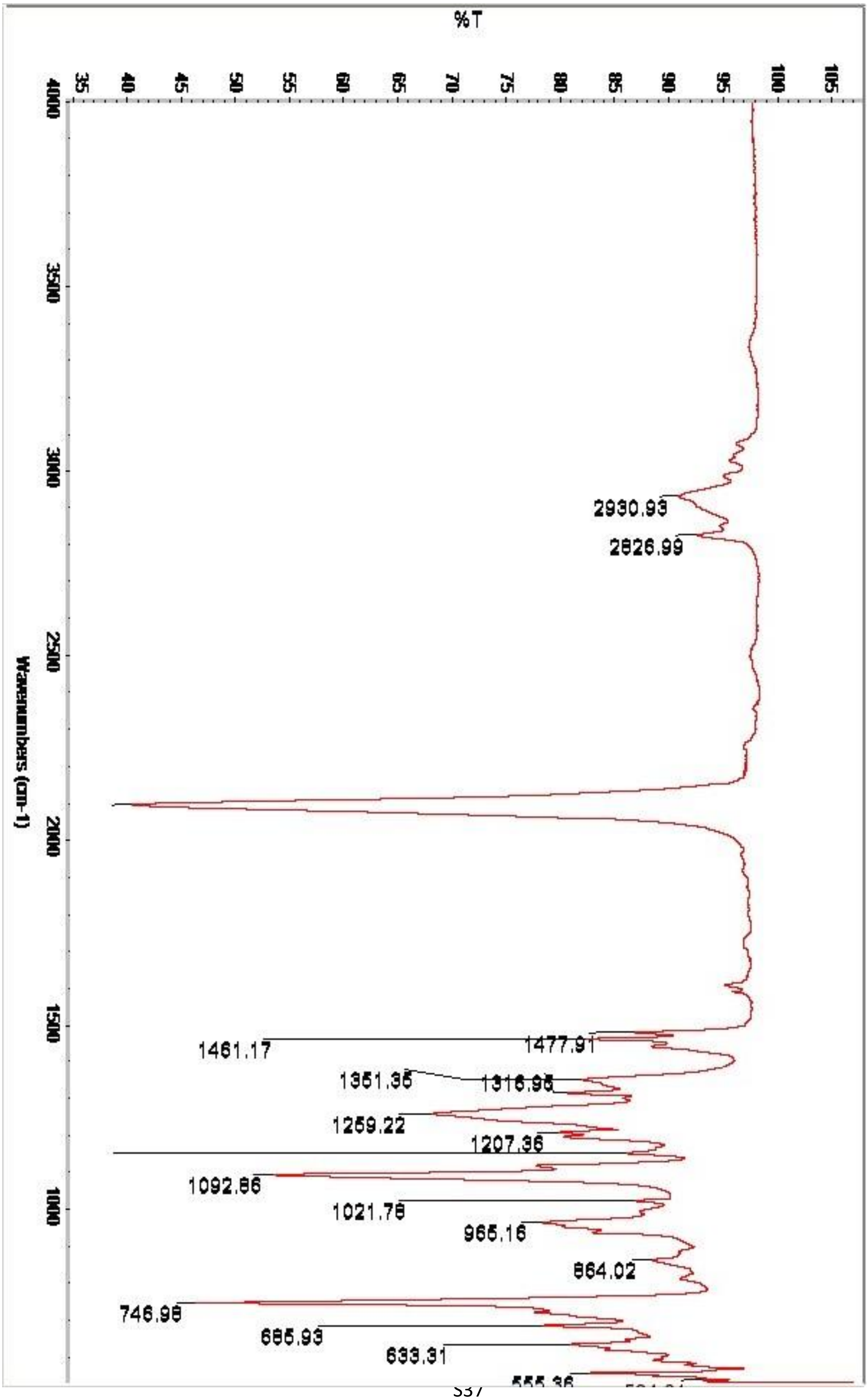
%T
Wavenumbers (cm-1)
2930.93
2826.99
1477.91
1461.17
1351.35
1316.95
1259.22
1207.36
1092.86
1021.76
965.16
864.02
746.98
685.93
633.31

Data for 2-azido-1-methoxy-1,2,3,4-tetrahydronaphthalene (**3h**)

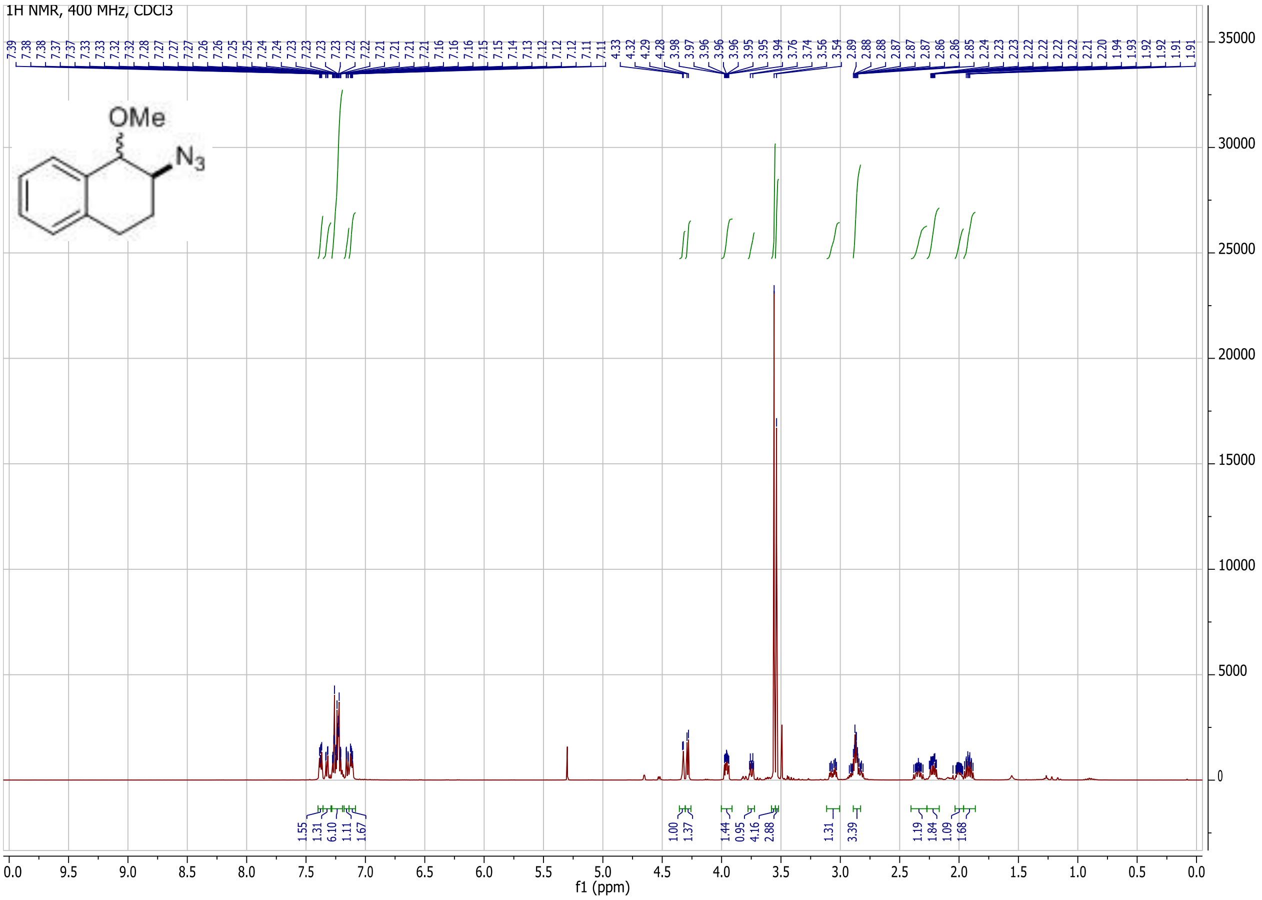

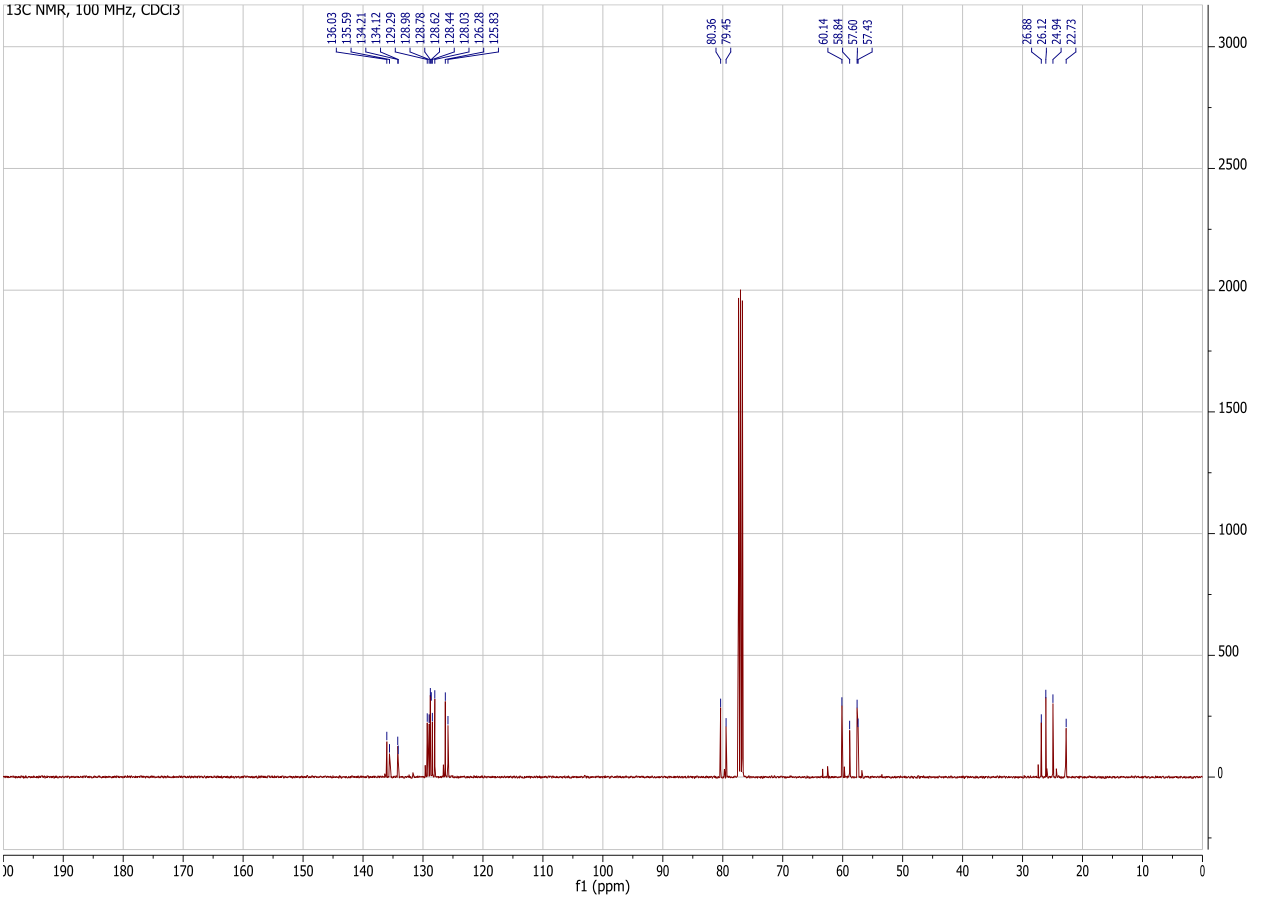

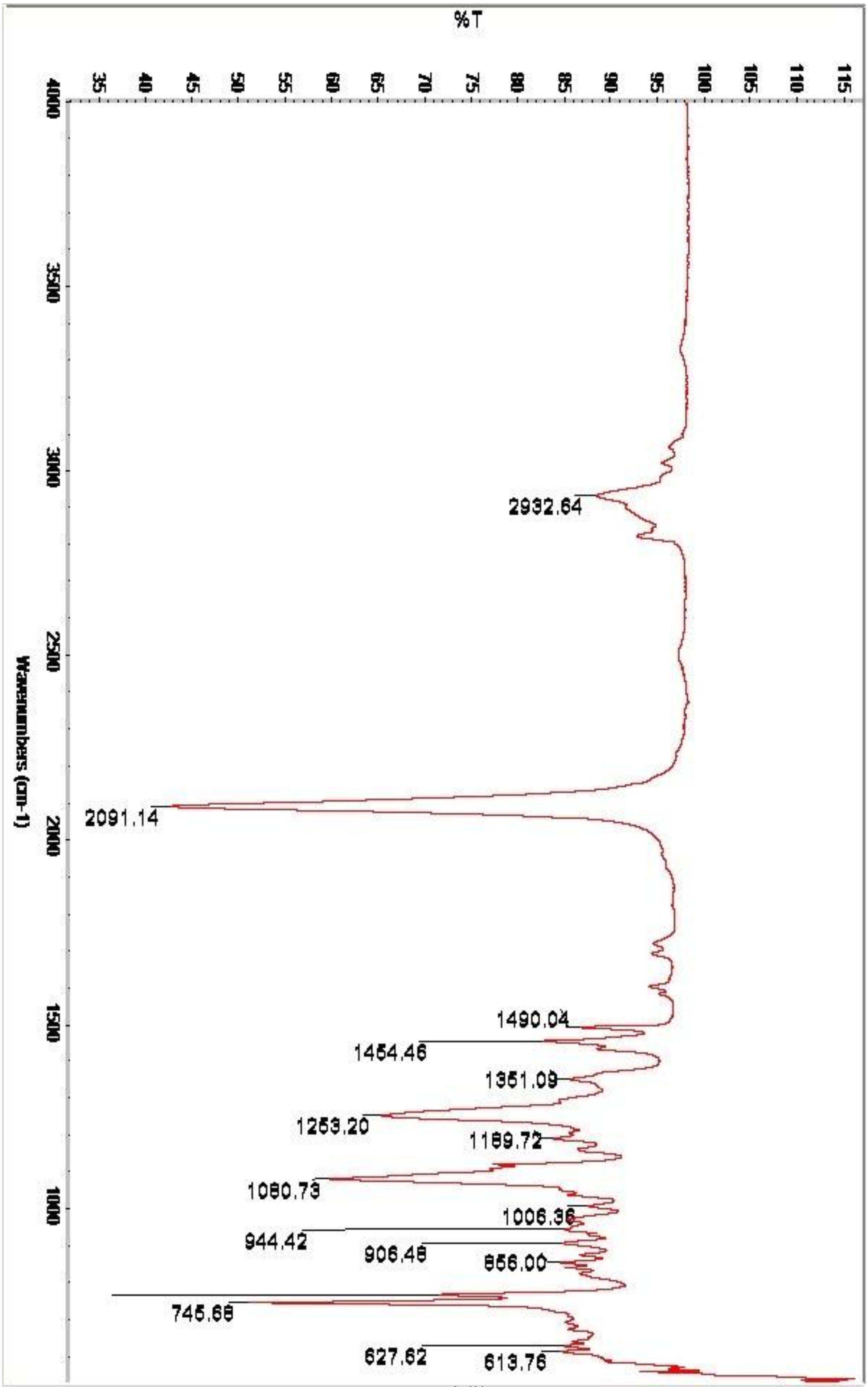
%T
Wavenumbers (cm-1)
4000
3500
3000
2500
2000
1500
1000
35
40
45
50
55
60
65
70
75
80
85
90
95
100
105
110
115
2932.64
2091.14
1490.04
1454.46
1351.09
1253.20
1169.72
1060.73
1006.36
944.42
906.46
856.00
745.66
627.62
613.76

Data for (1-azido-2-methoxypropan-2-yl)benzene (**3i**)

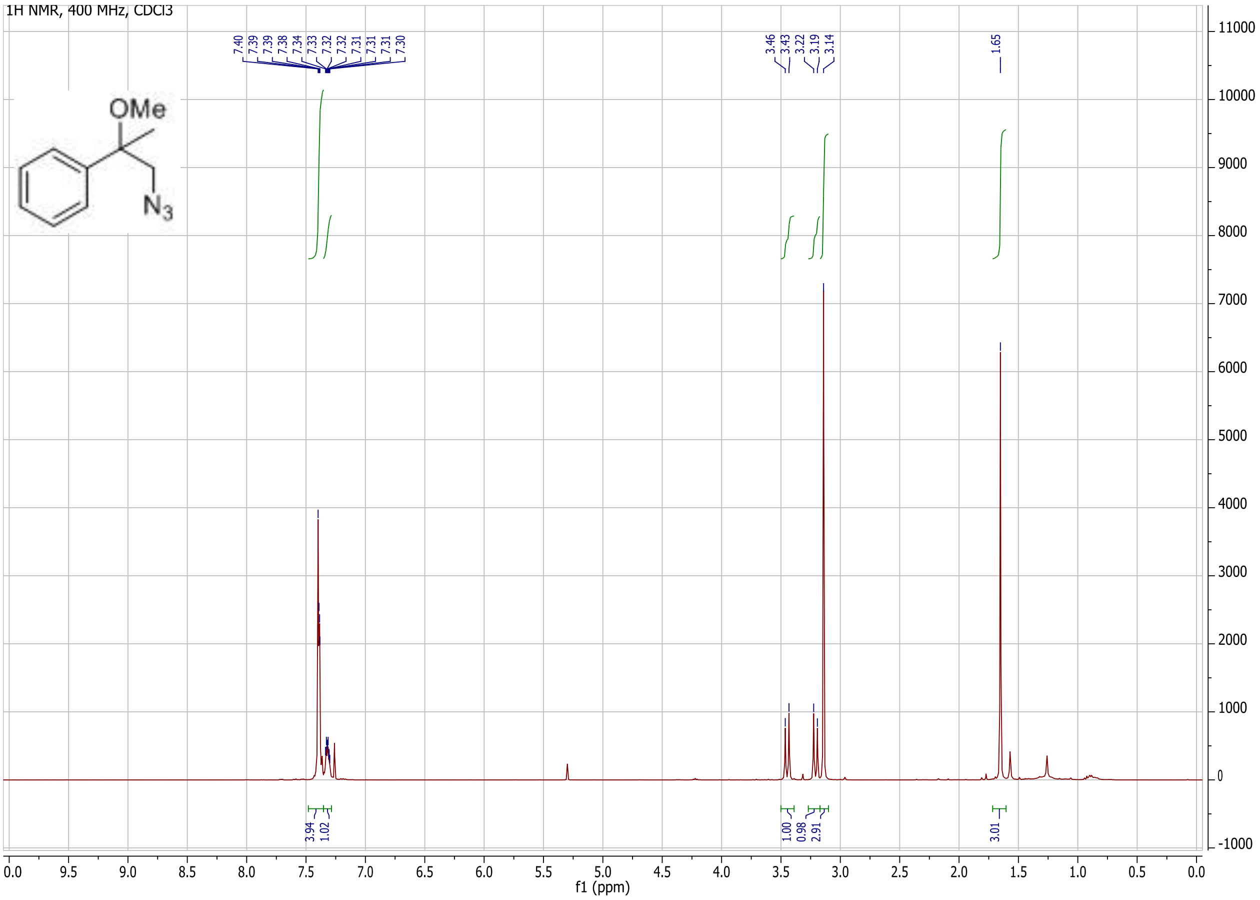

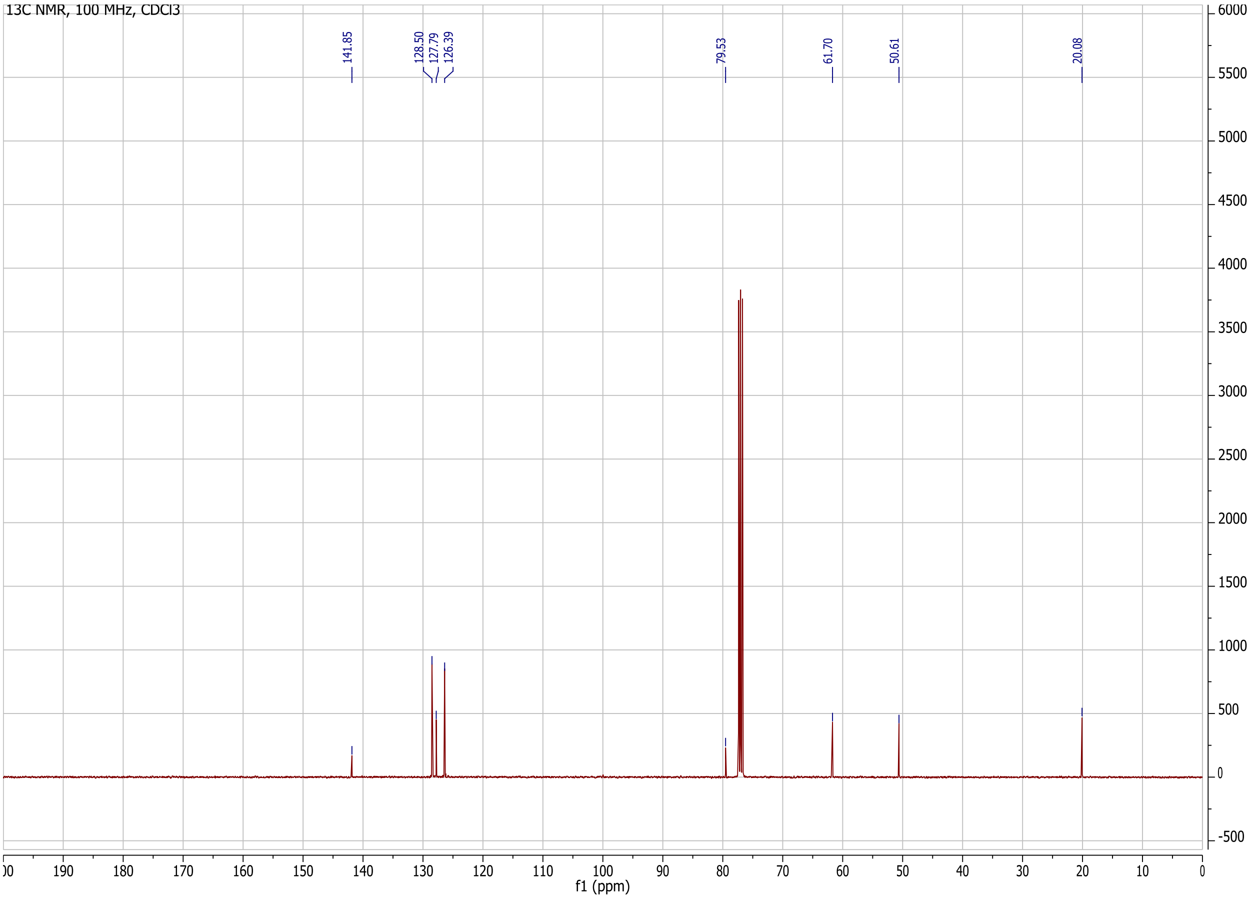

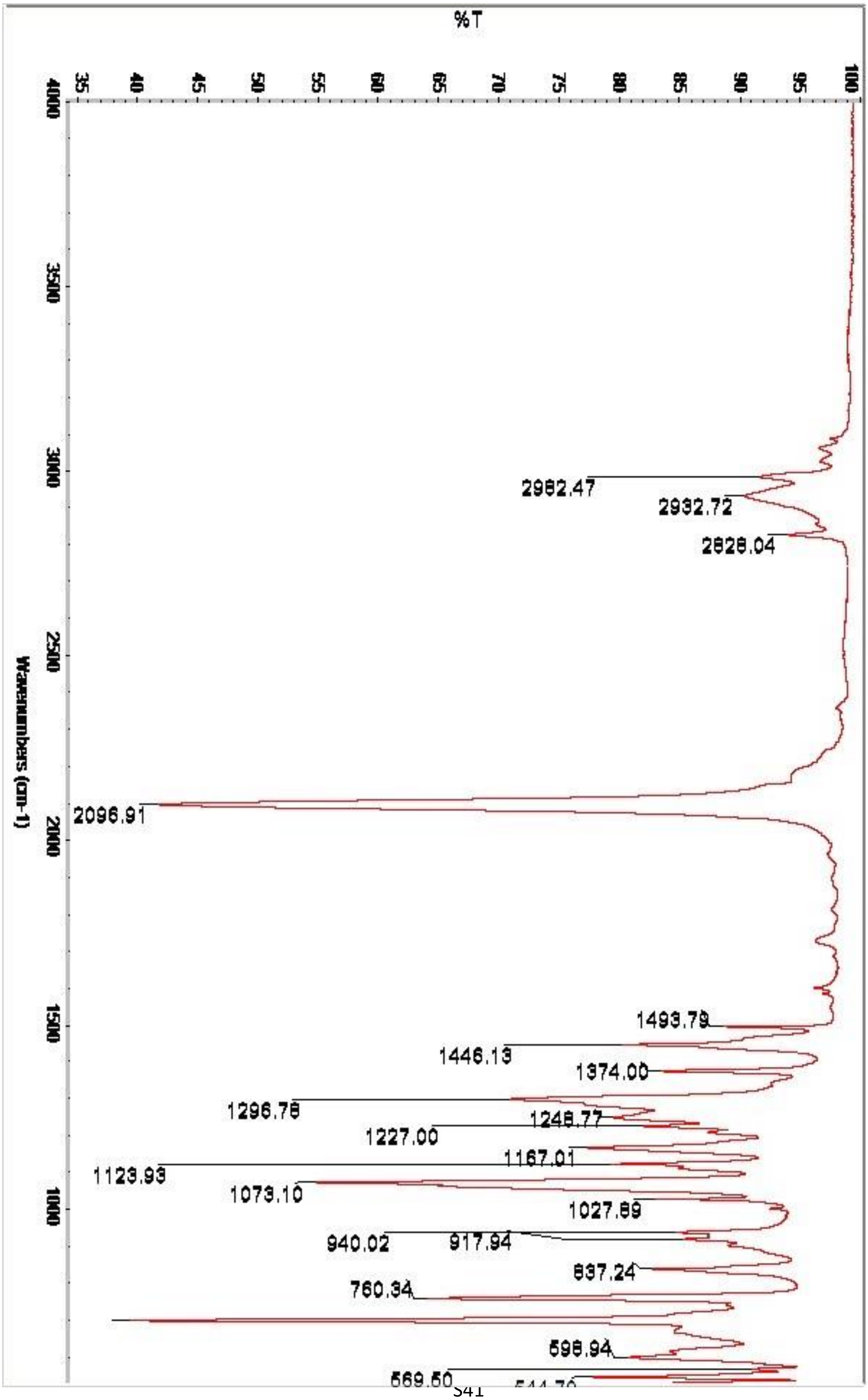
%T
Wavenumbers (cm-1)
2982.47
2932.72
2828.04
2096.91
1493.79
1446.13
1374.00
1296.78
1248.77
1227.00
1167.01
1123.93
1073.10
1027.89
940.02
917.94
837.24
760.34
598.94
569.50

Data for 2-azido-1,1-diphenyl-1-methoxyethane (**3j**)

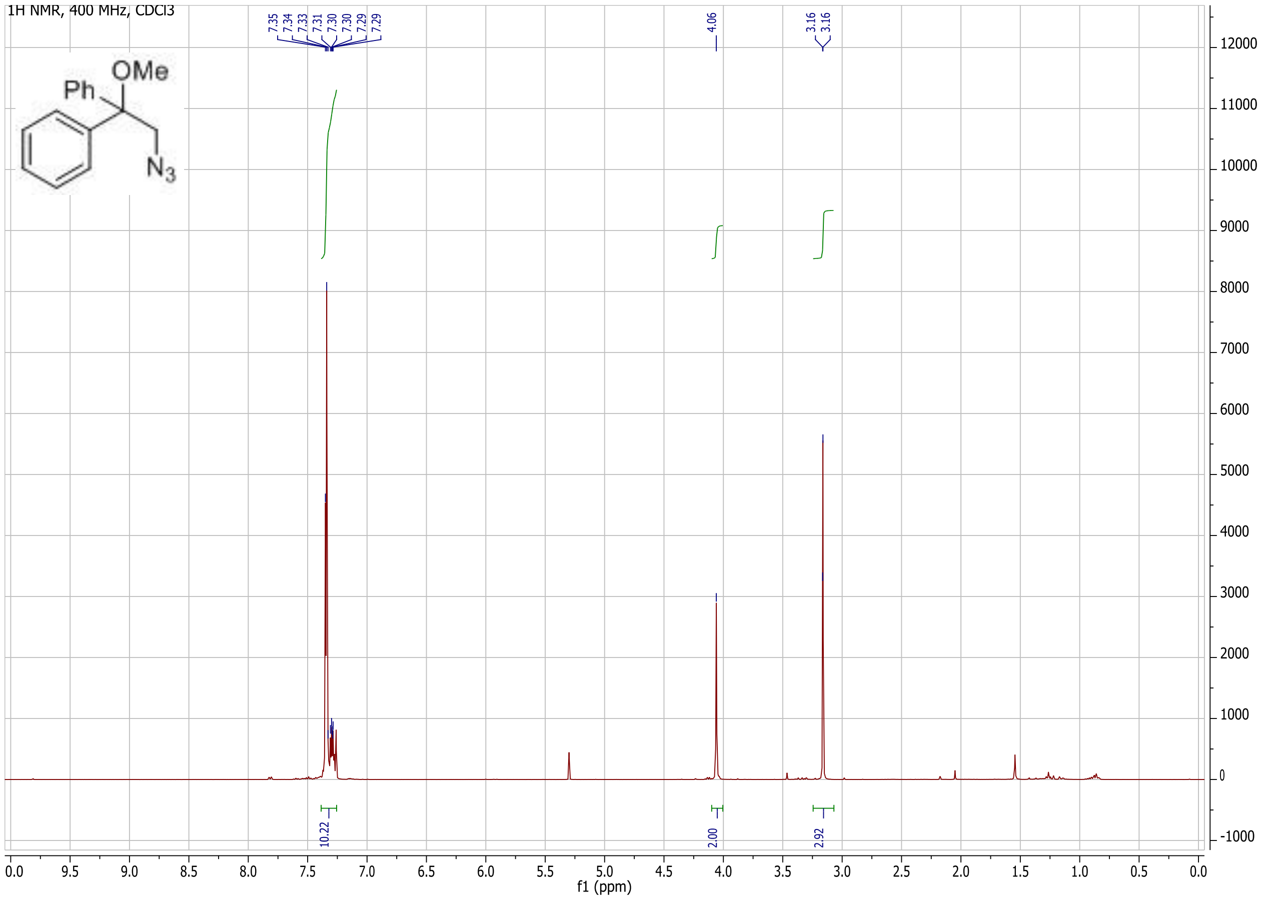

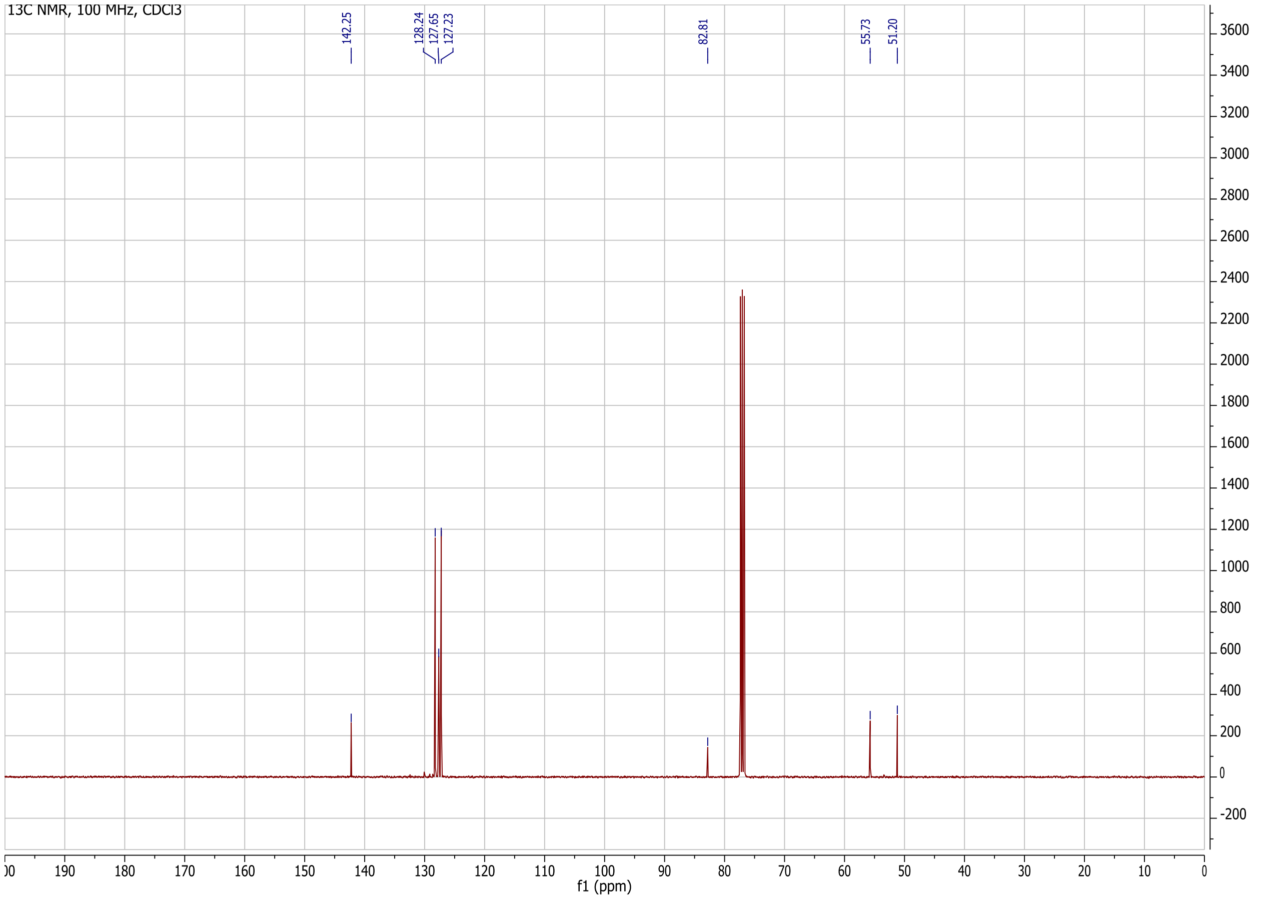

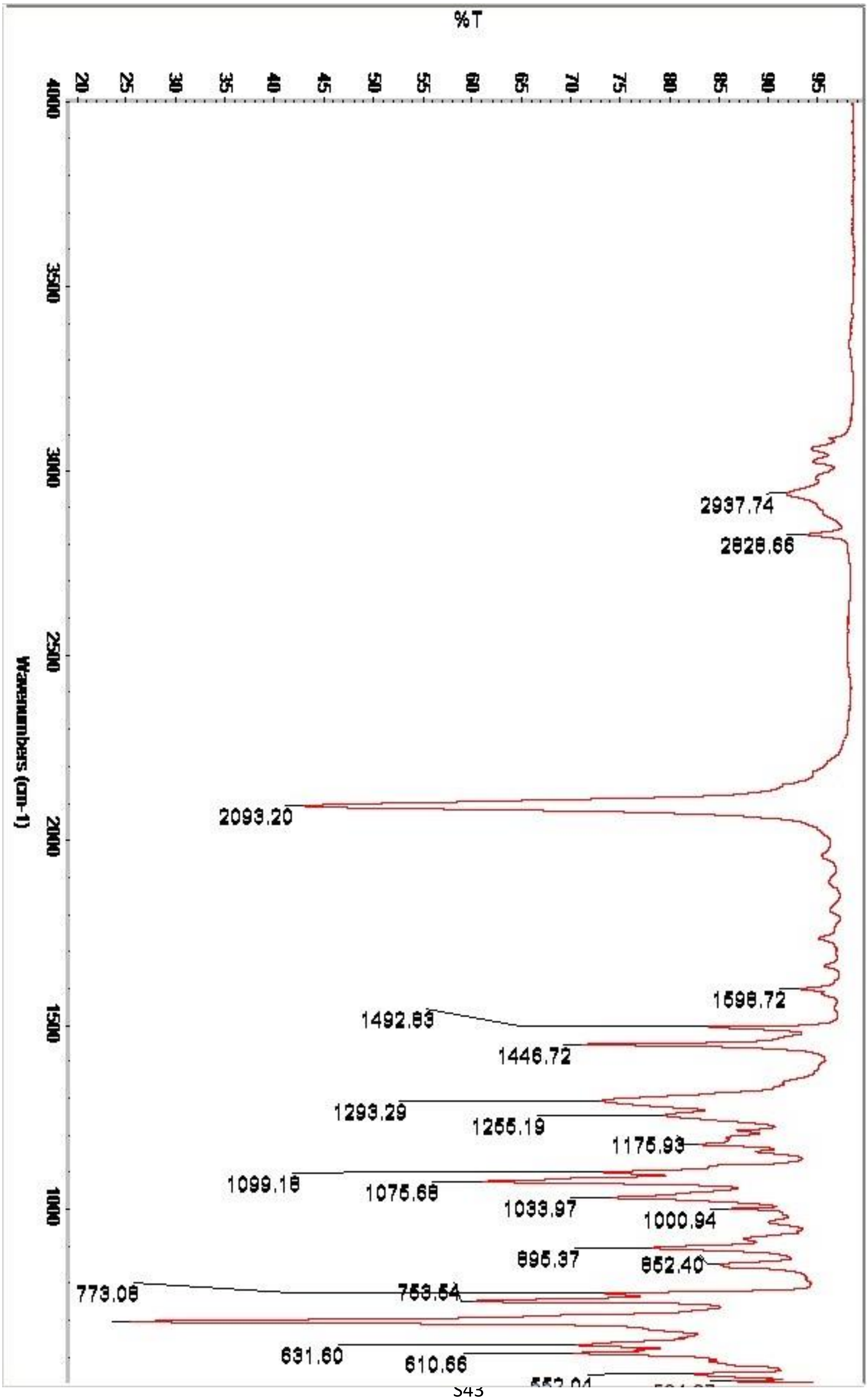
%T
Wavenumbers (cm-1)
2937.74
2828.66
2093.20
1596.72
1492.83
1446.72
1293.29
1255.19
1175.93
1099.16
1075.68
1033.97
1000.94
895.37
852.40
773.06
753.54
631.60
610.66

Data for (2-azido-1-methoxycyclohexyl)benzene (**3k**)

Cis-isomer

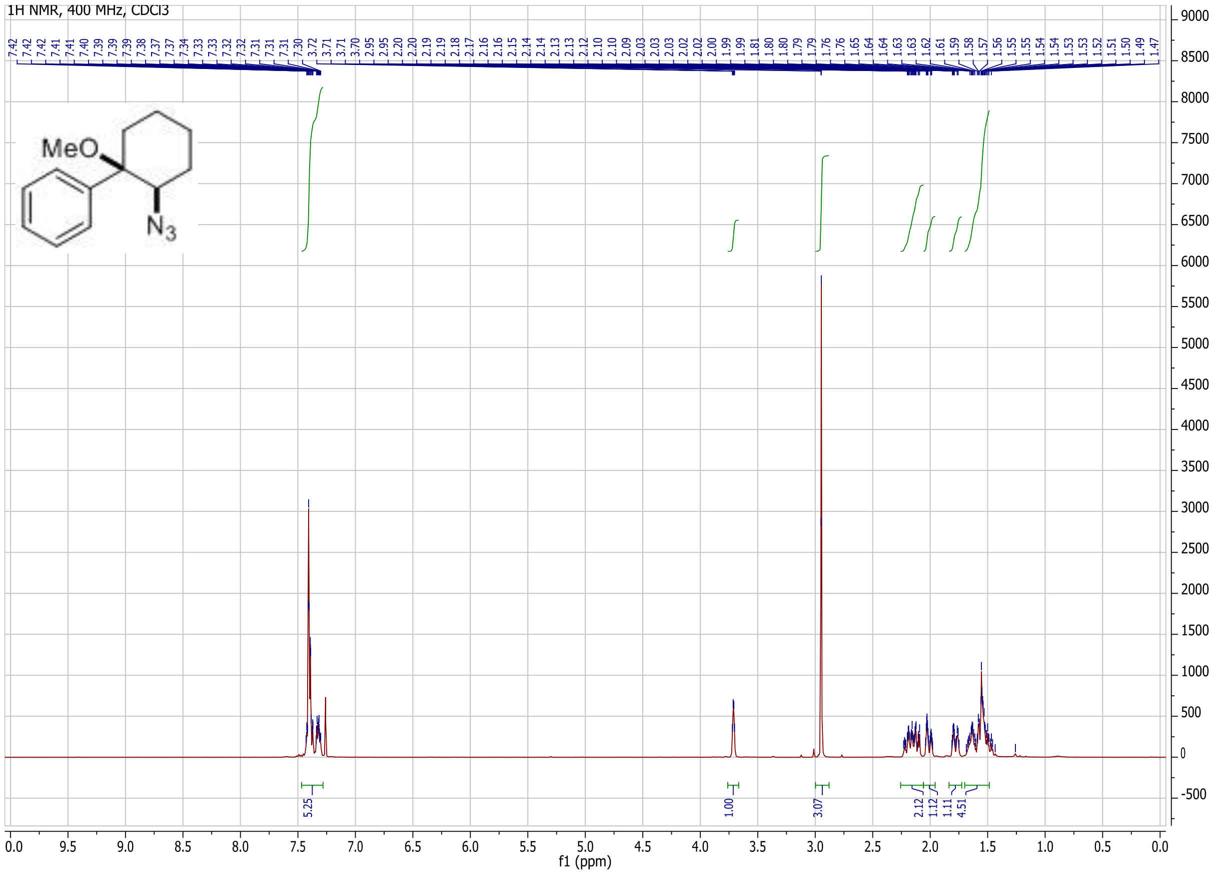

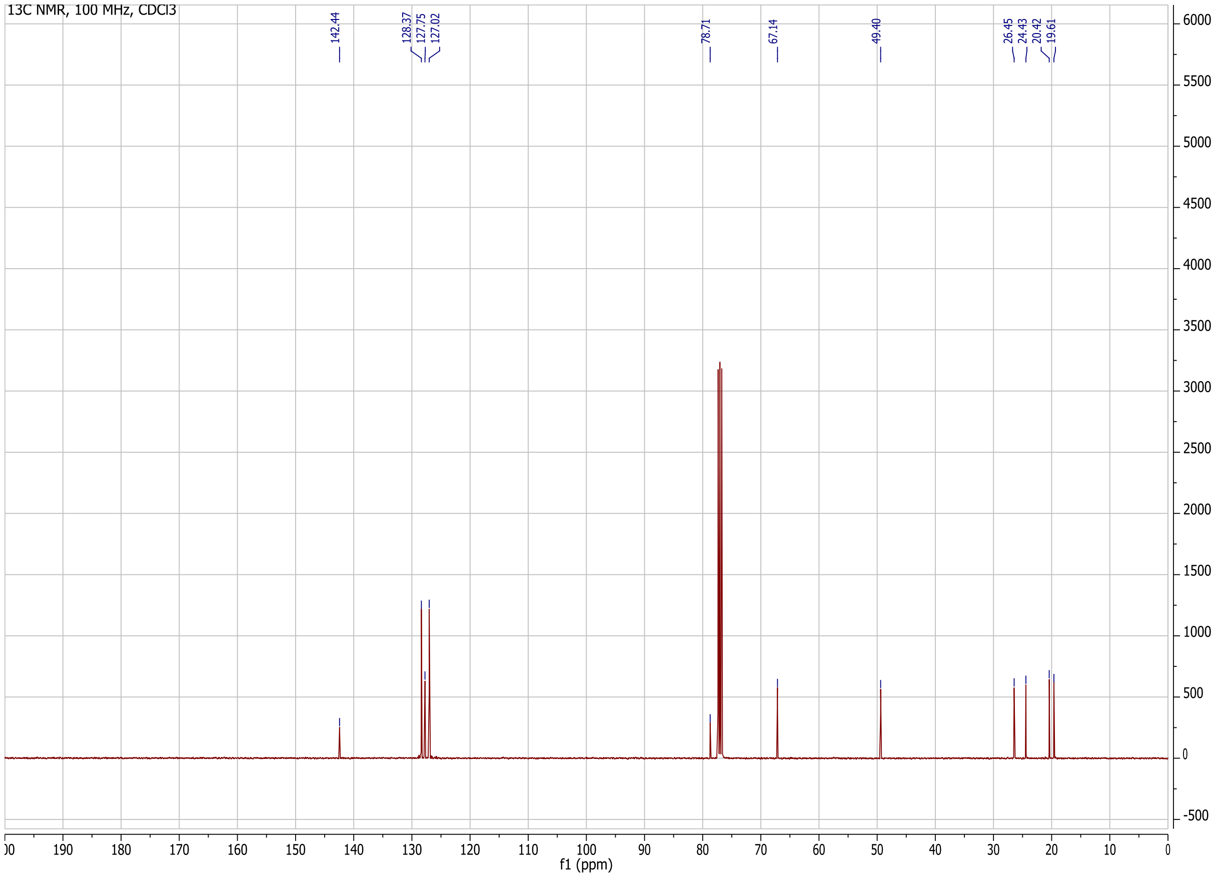

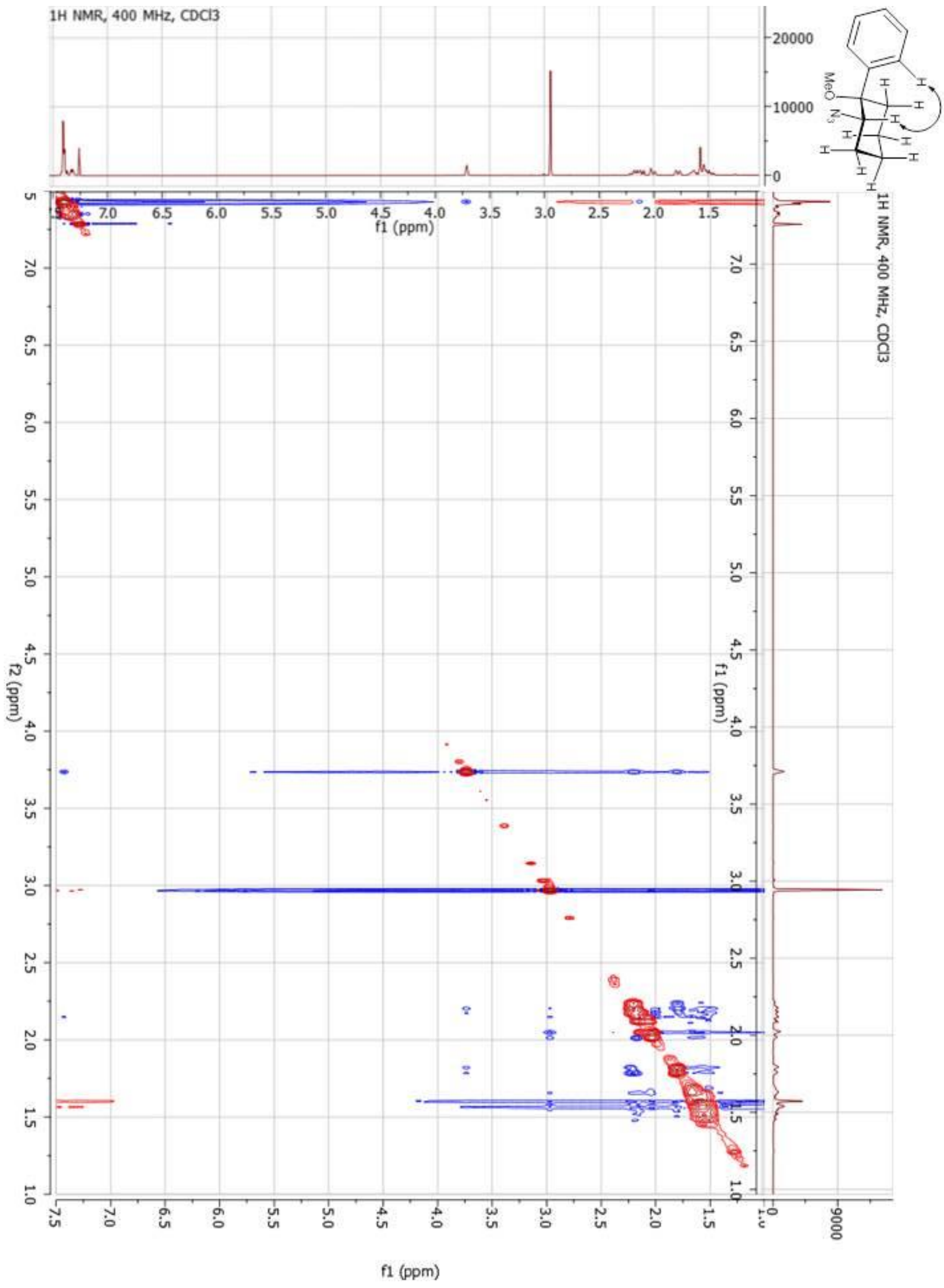
1H NMR, 400 MHz, CDCl3
f1 (ppm)
f2 (ppm)
MeO
N3

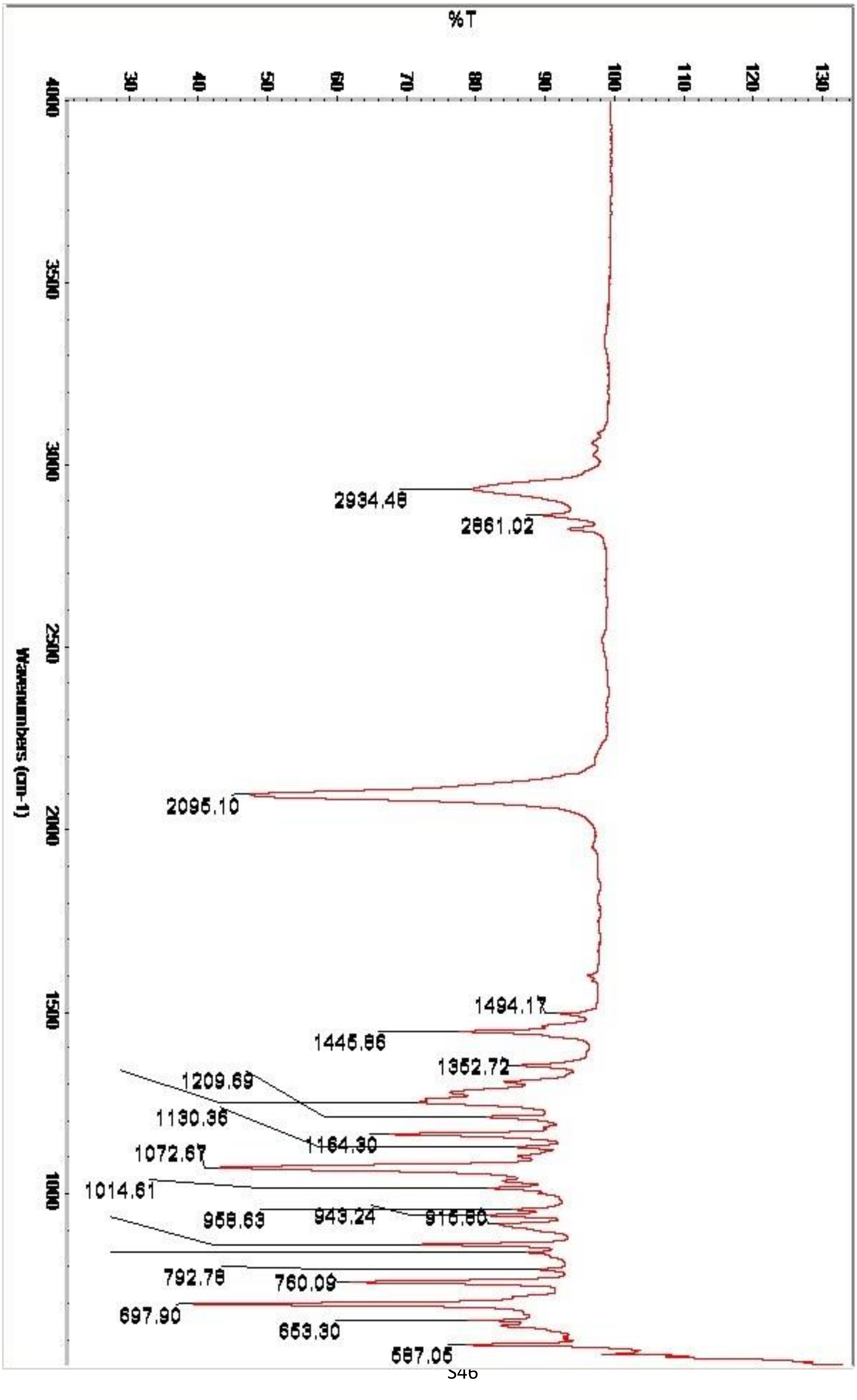
%T
Wavenumbers (cm-1)
2934.48
2861.02
2095.10
1494.17
1445.86
1352.72
1209.69
1130.36
1164.30
1072.67
1014.61
958.63
943.24
916.80
792.76
760.09
697.90
653.30
587.05

Trans-isomer

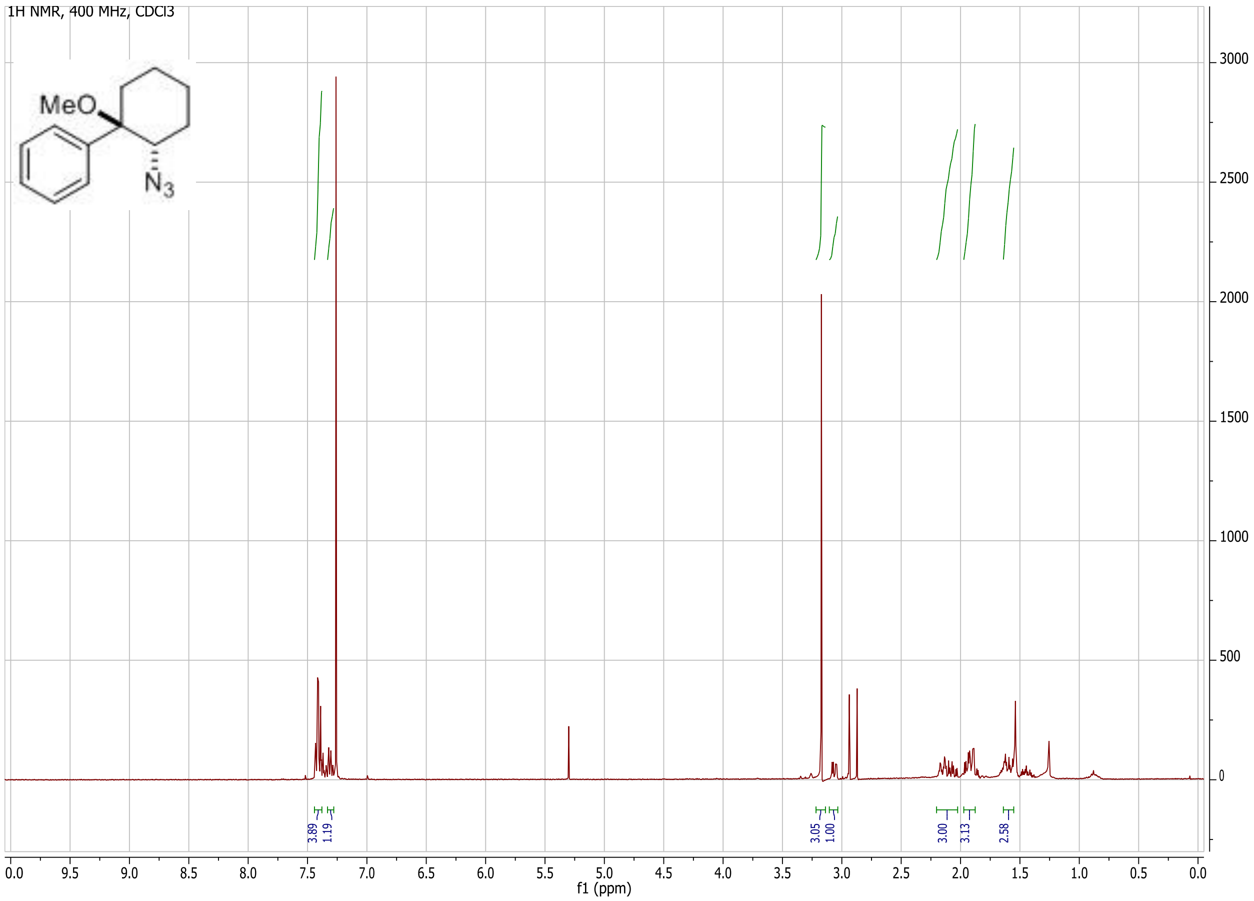

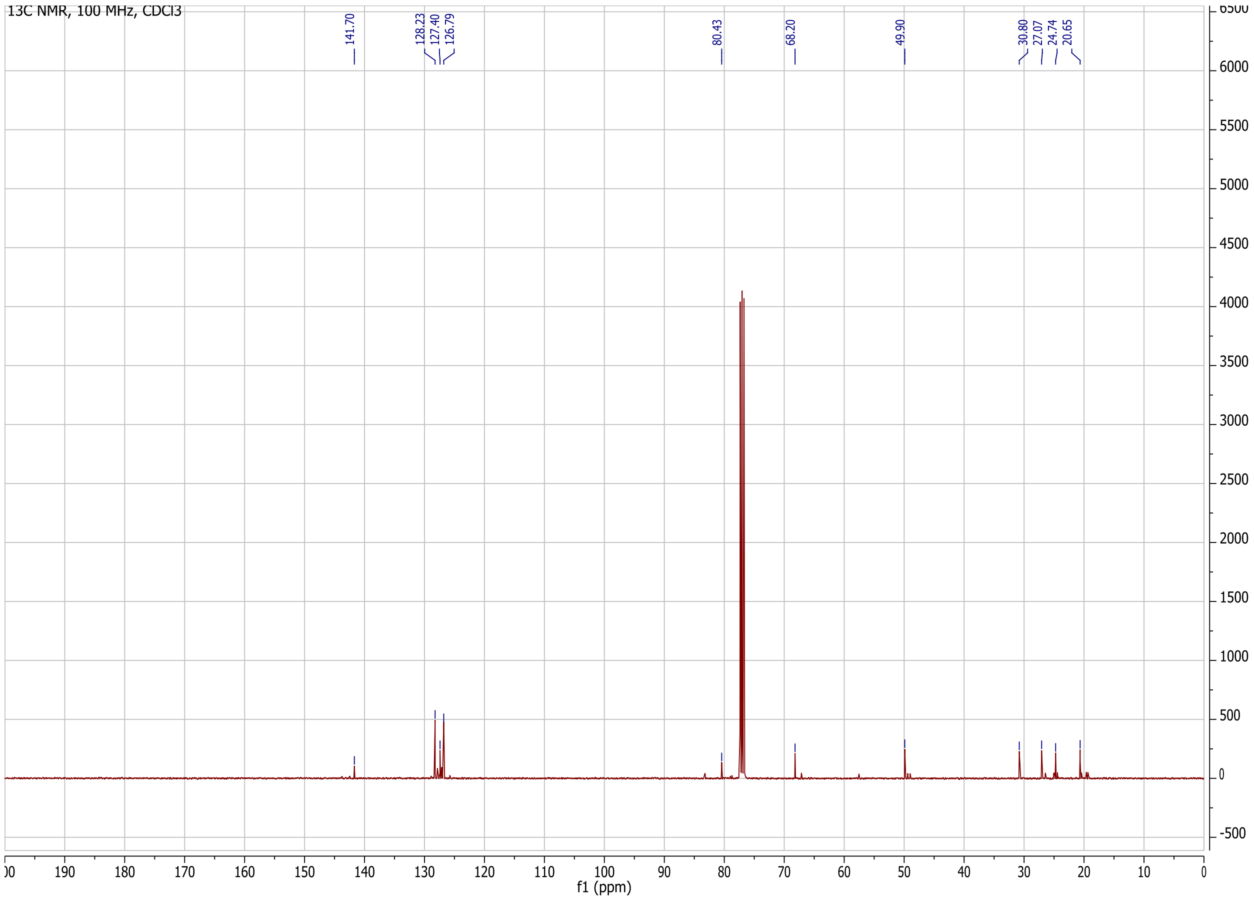

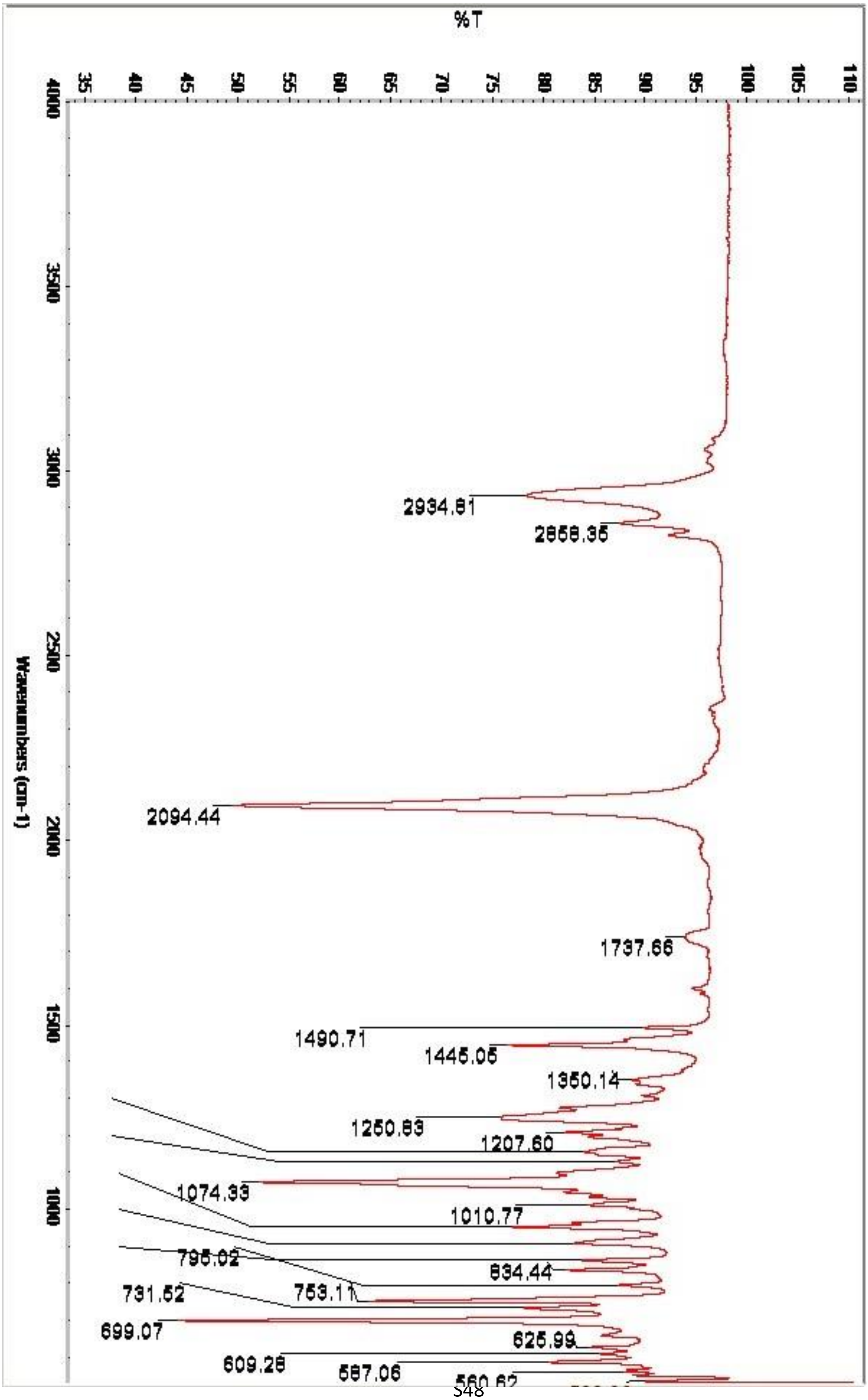
%T
Wavenumbers (cm-1)
2934.81
2858.35
2094.44
1737.66
1490.71
1445.05
1350.14
1250.63
1207.60
1074.33
1010.77
795.02
834.44
753.11
731.52
699.07
625.99
609.26
587.06
560.62

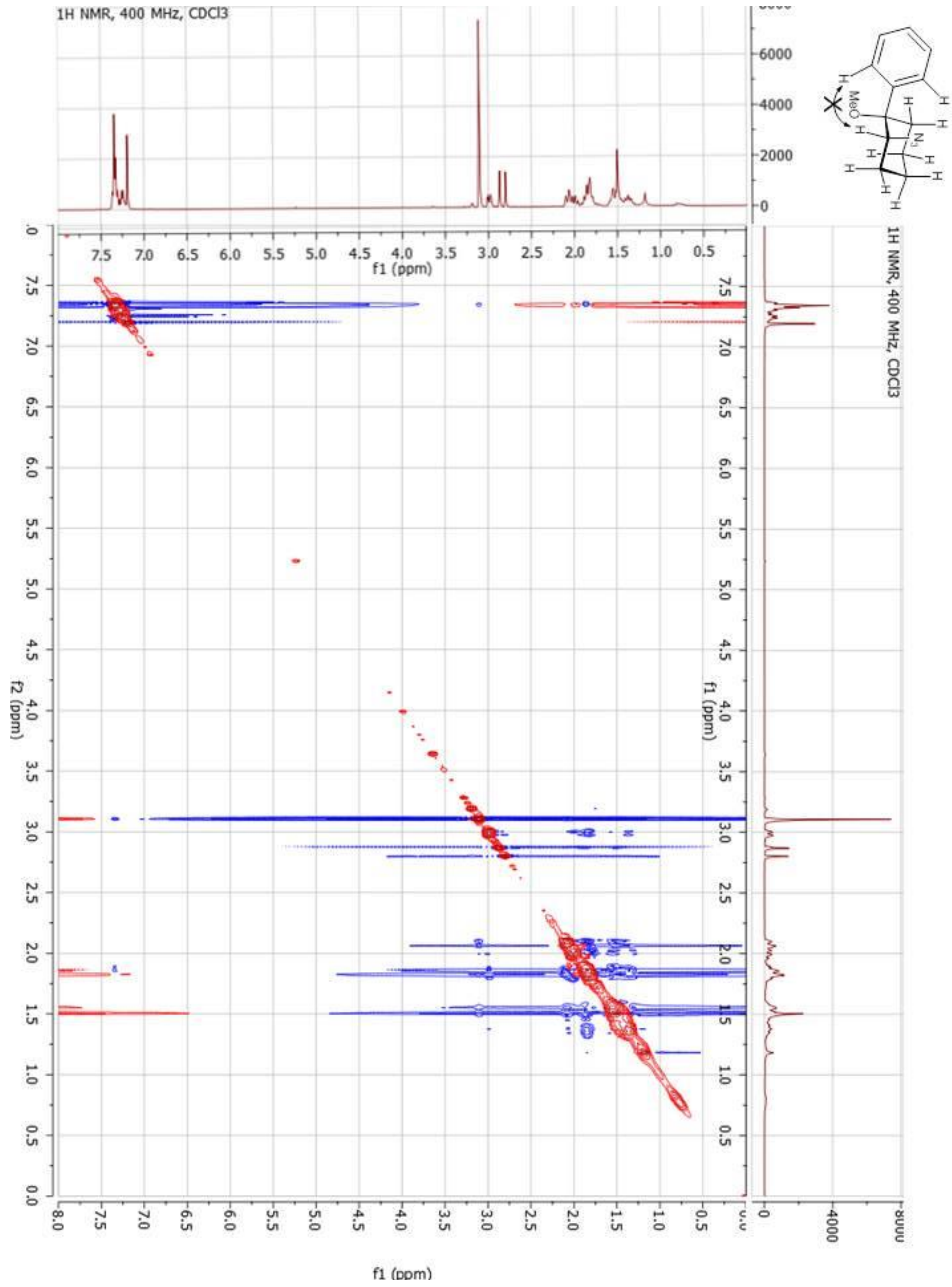
1H NMR, 400 MHz, CDCl3
f1 (ppm)
f2 (ppm)
MeO
N3
1H NMR, 400 MHz, CDCl3

Data for (2-azido-1-methoxypropyl)benzene (**3l**)

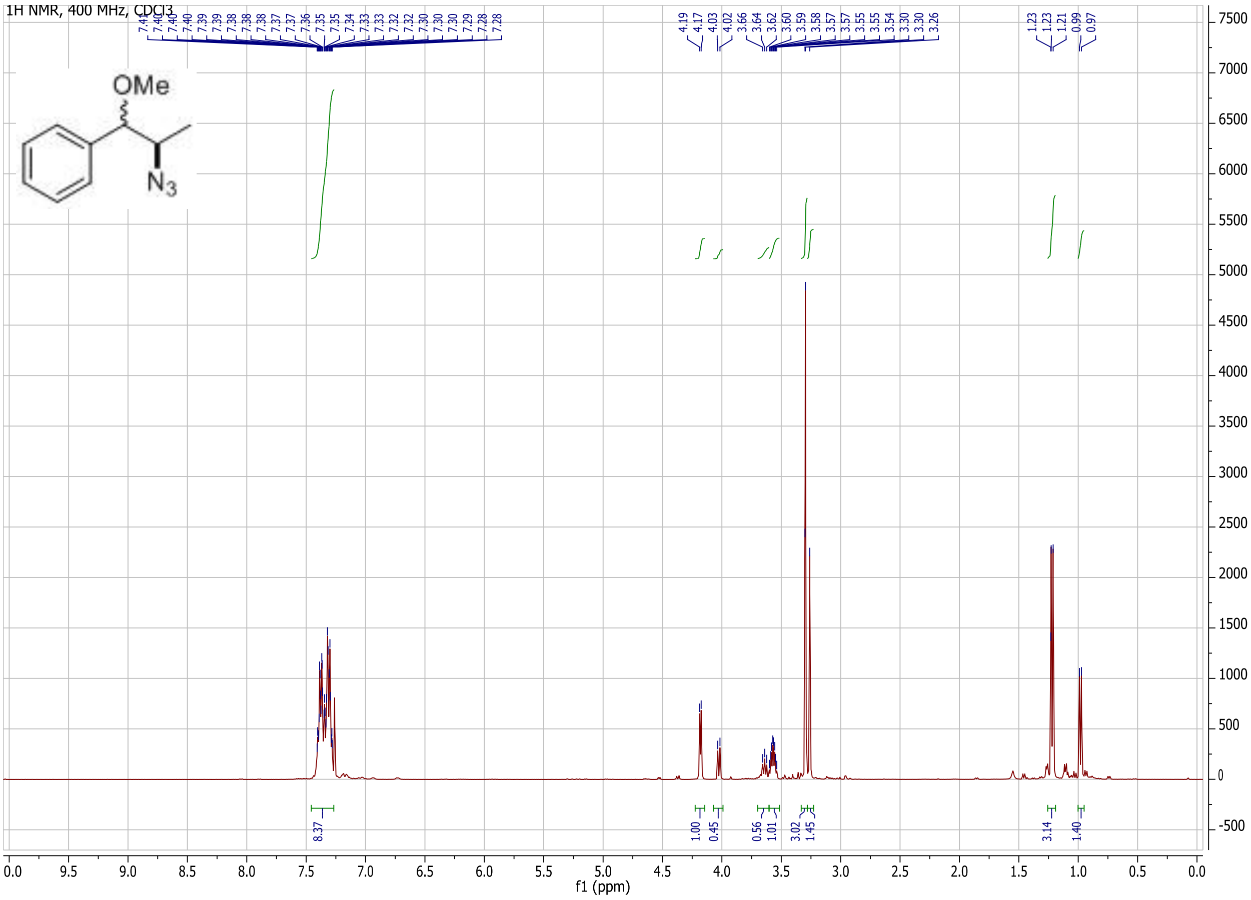

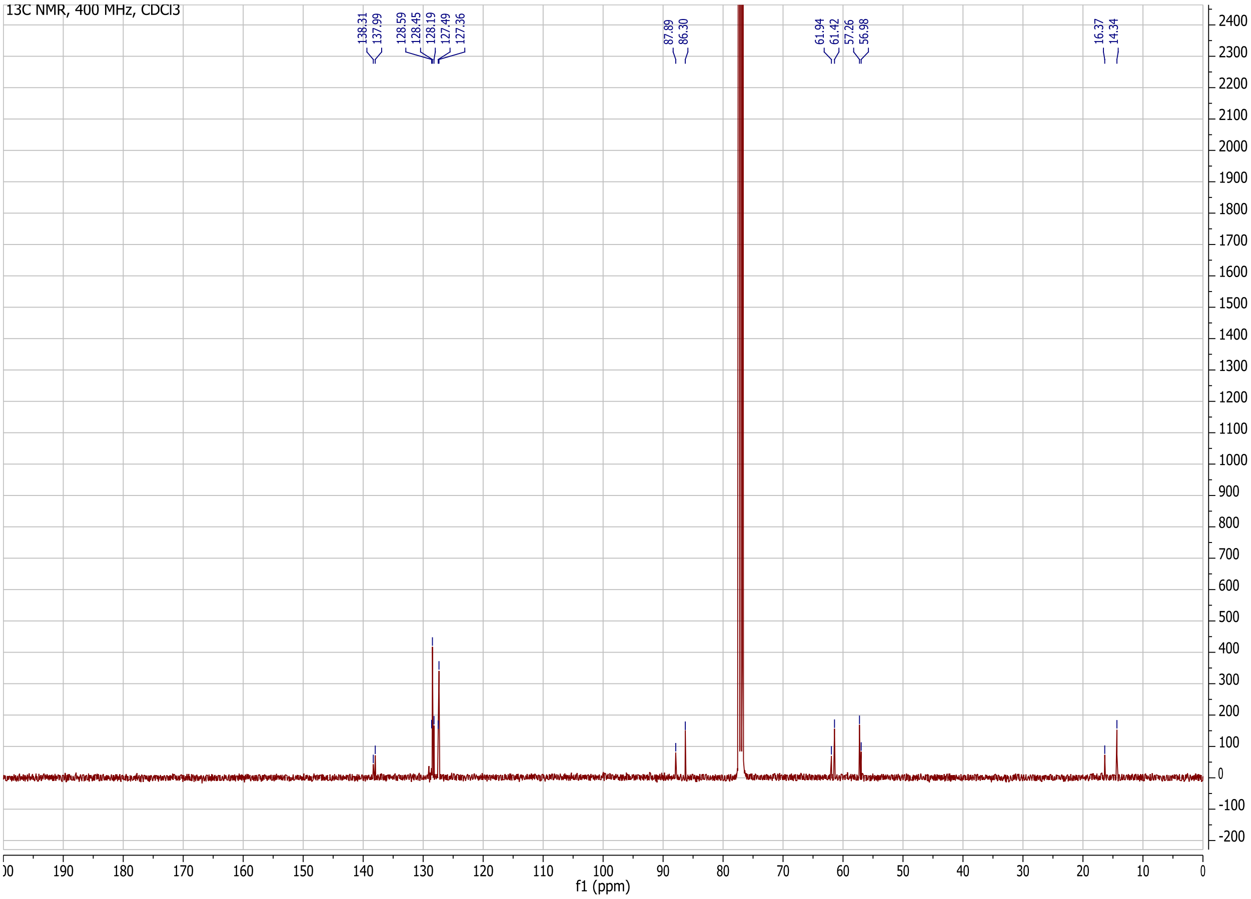

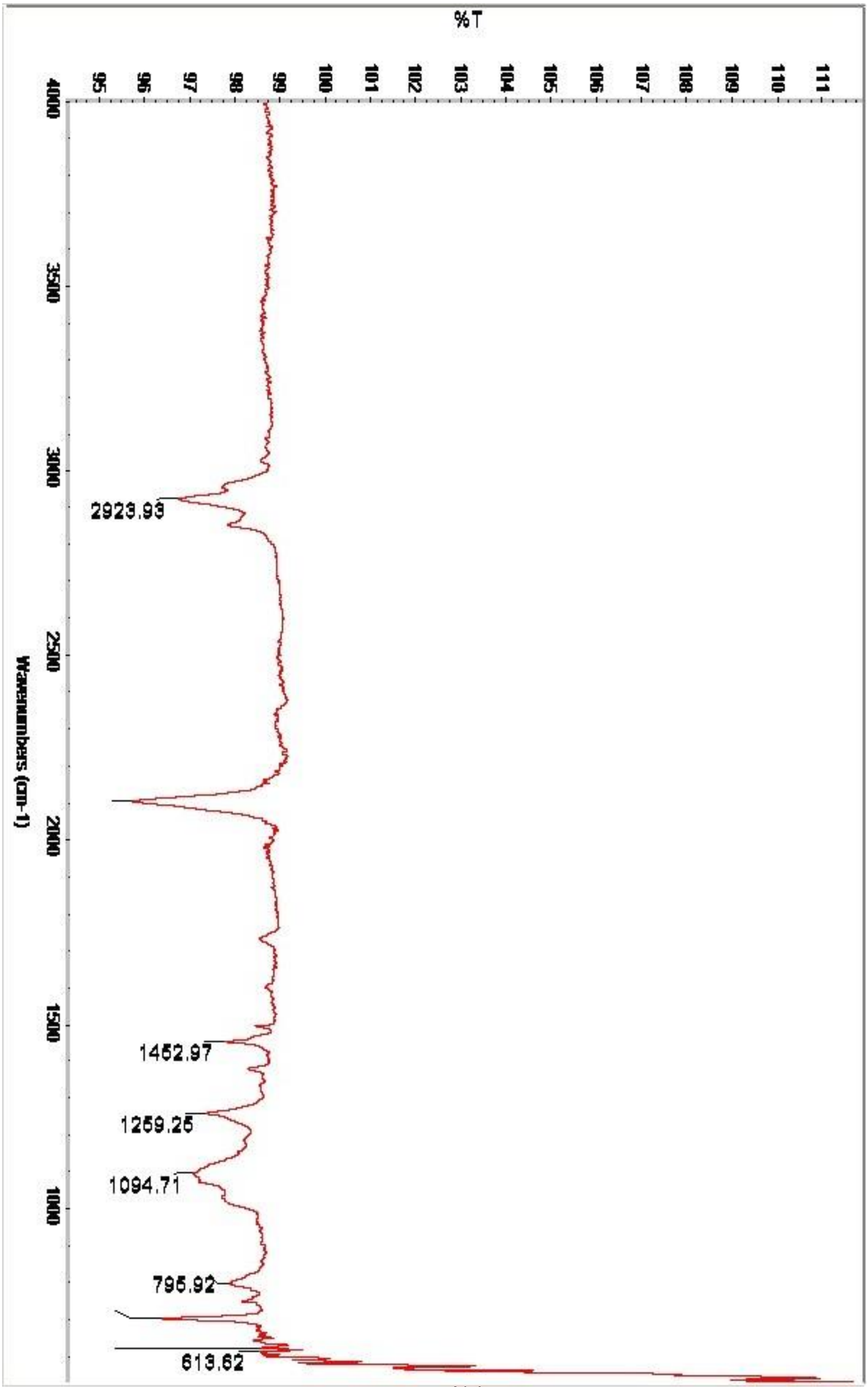
%T
Wavenumbers (cm-1)
2923.93
1452.97
1259.25
1094.71
795.92
613.62

Data for 2-azido-1,2-diphenyl-1-methoxyethane (**3m**)

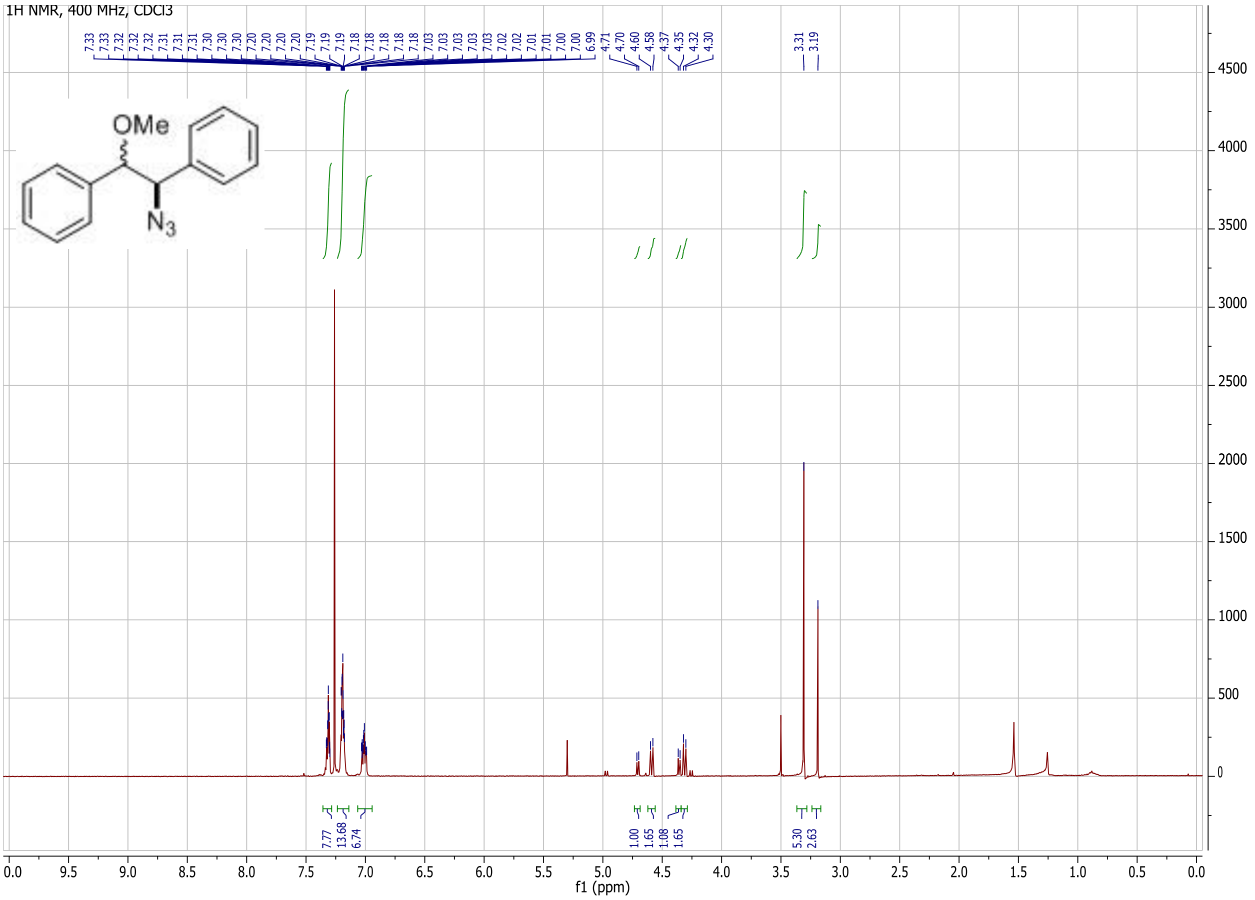

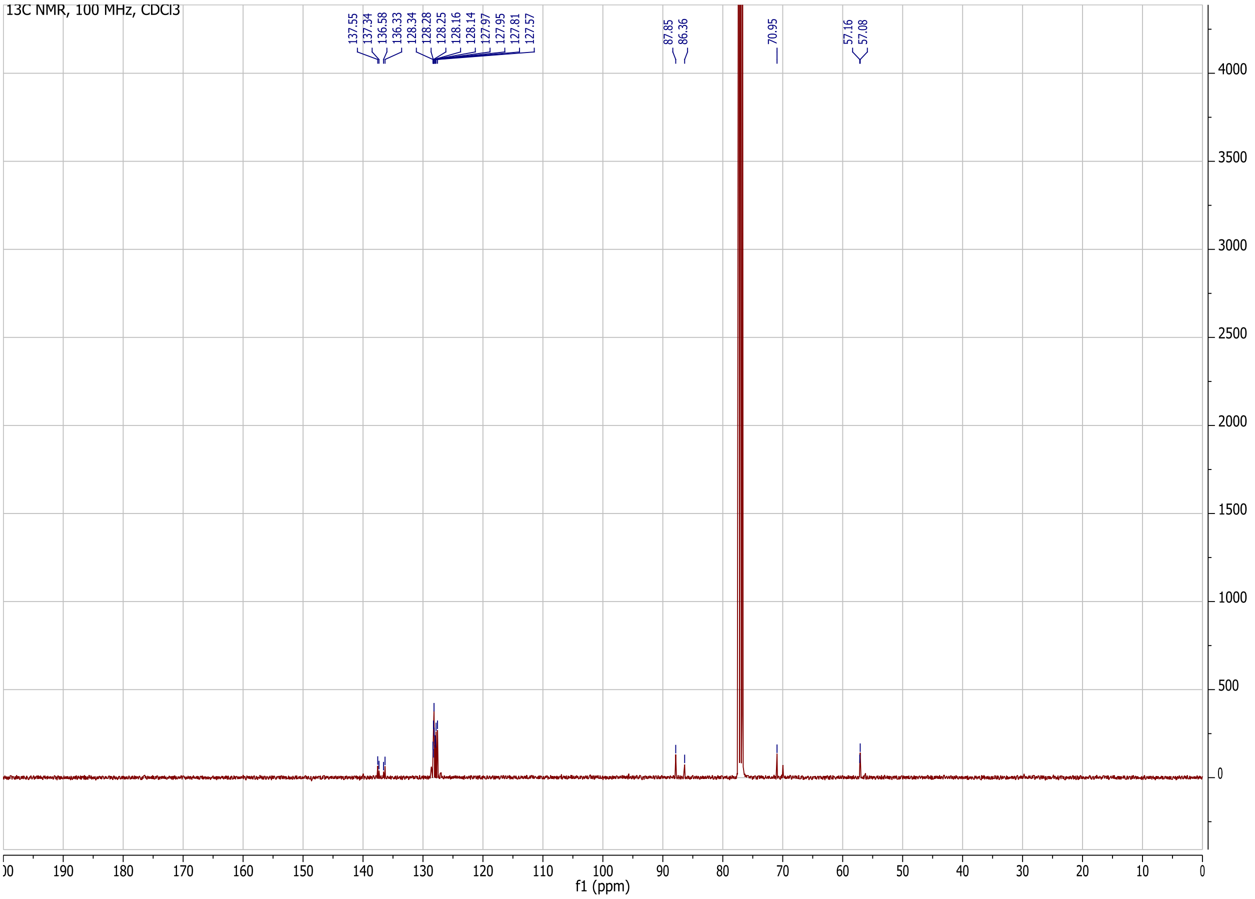

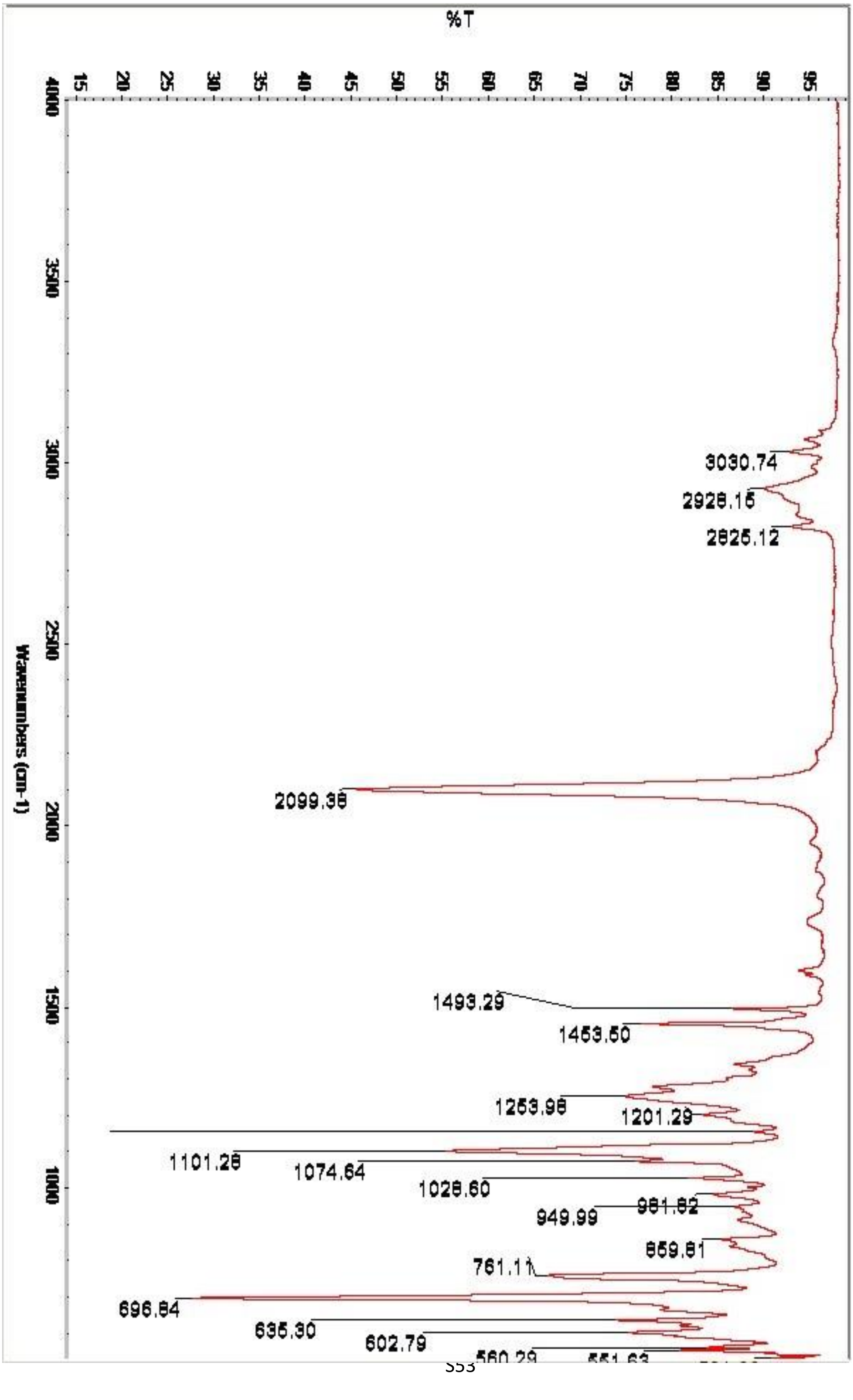
%T
Wavenumbers (cm-1)
3030.74
2926.15
2825.12
2099.38
1493.29
1453.50
1253.98
1201.29
1101.28
1074.64
1028.60
981.82
949.99
859.81
761.11
696.84
635.30
602.79
560.29

Data for (2-azido-1-methoxypropyl)-4-methoxybenzene (**3n**)

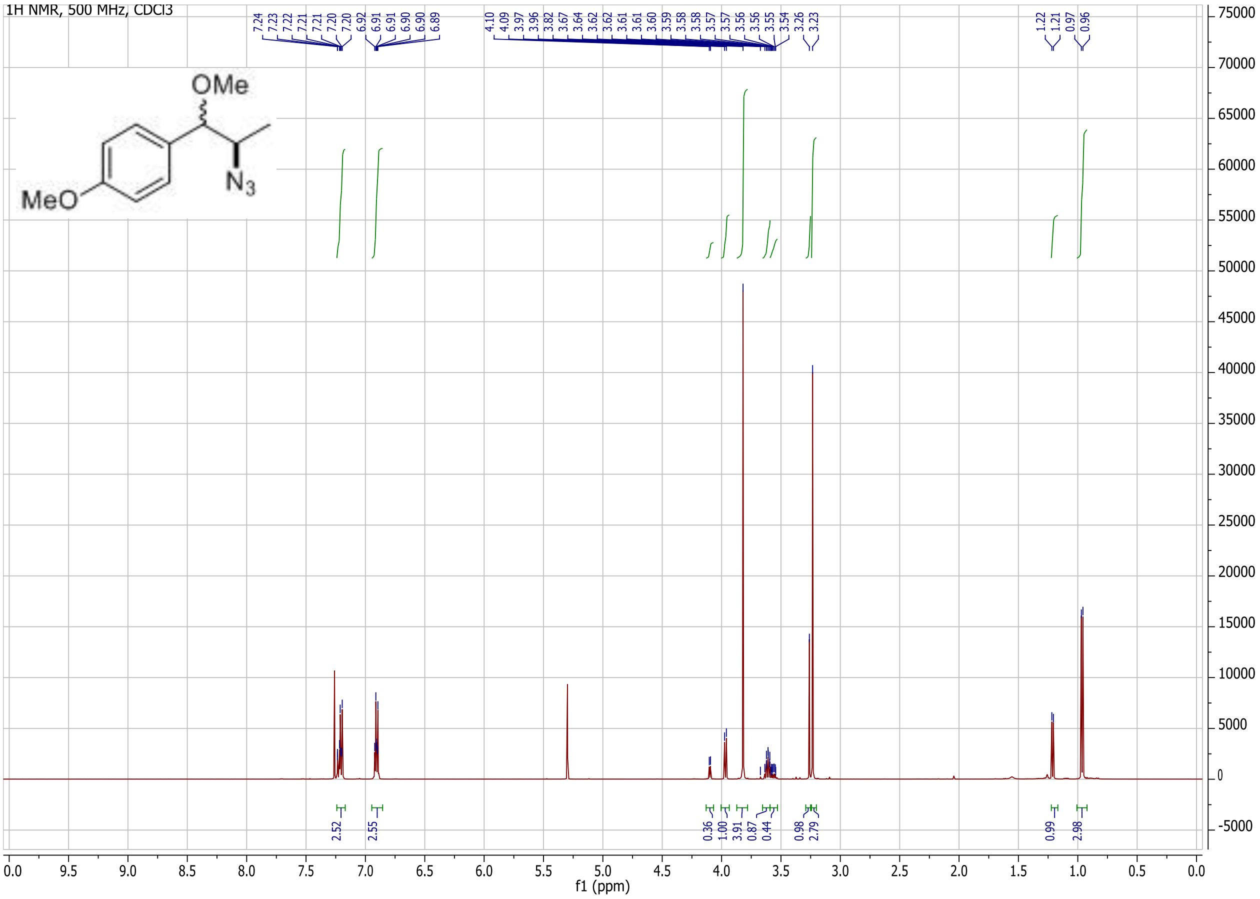

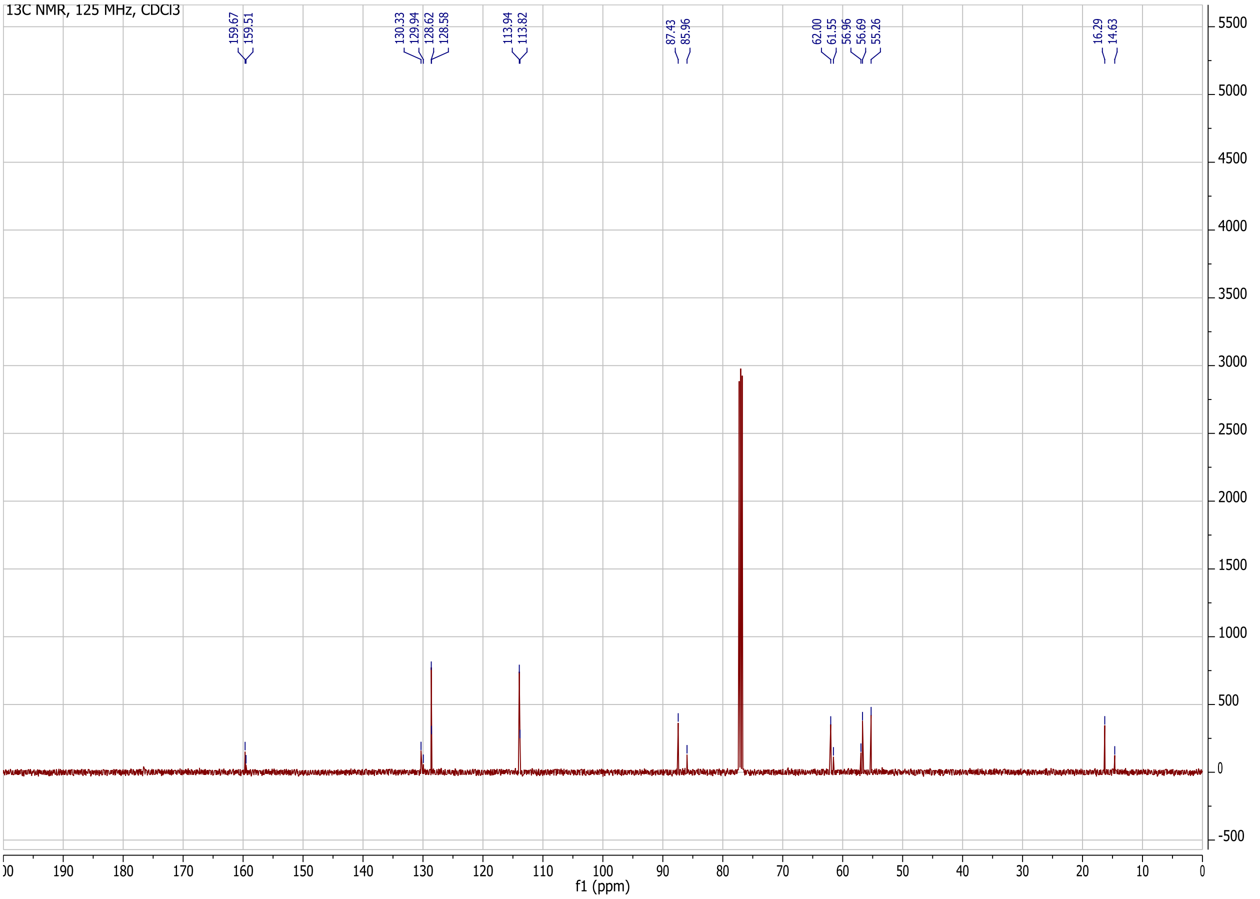

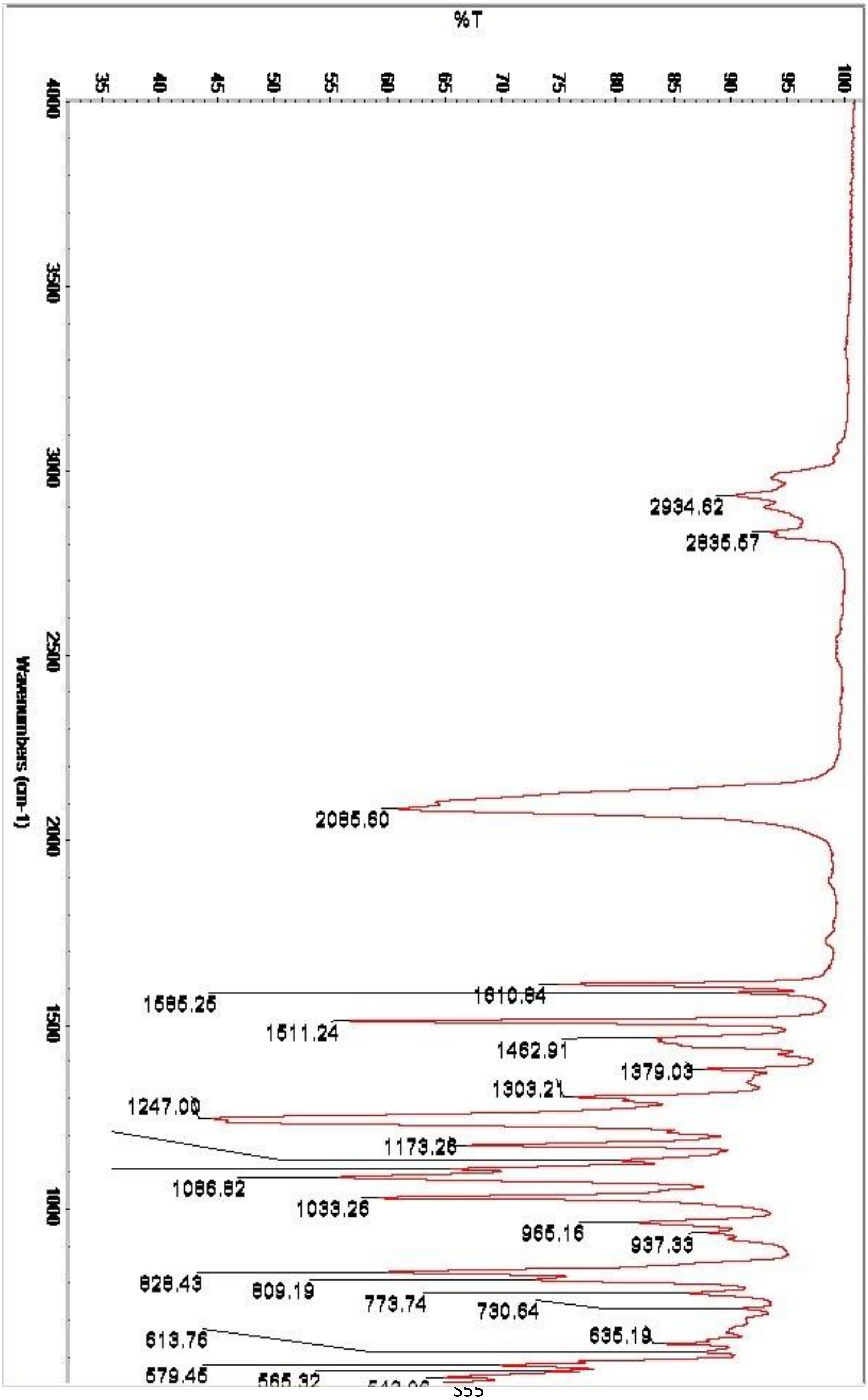
%T
Wavenumbers (cm-1)
2934.62
2835.57
2085.60
1610.64
1585.25
1511.24
1462.91
1379.03
1303.21
1247.00
1173.26
1086.82
1033.26
965.16
937.33
828.43
809.19
773.74
730.64
636.19
613.76
579.45
565.32

Data for Methyl 1-(2-azido-1-methoxyethyl)-4-fluorobenzene (**3o**)

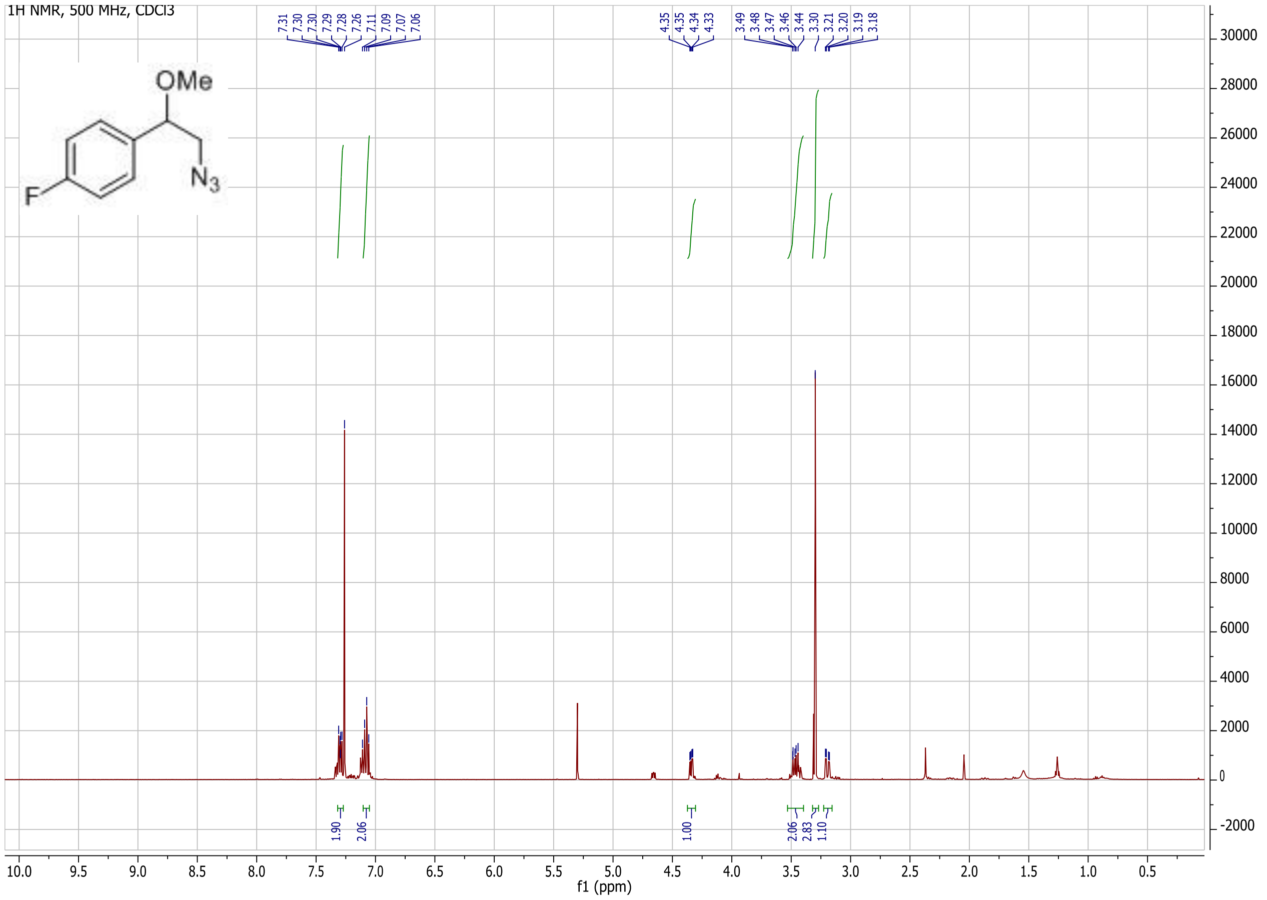

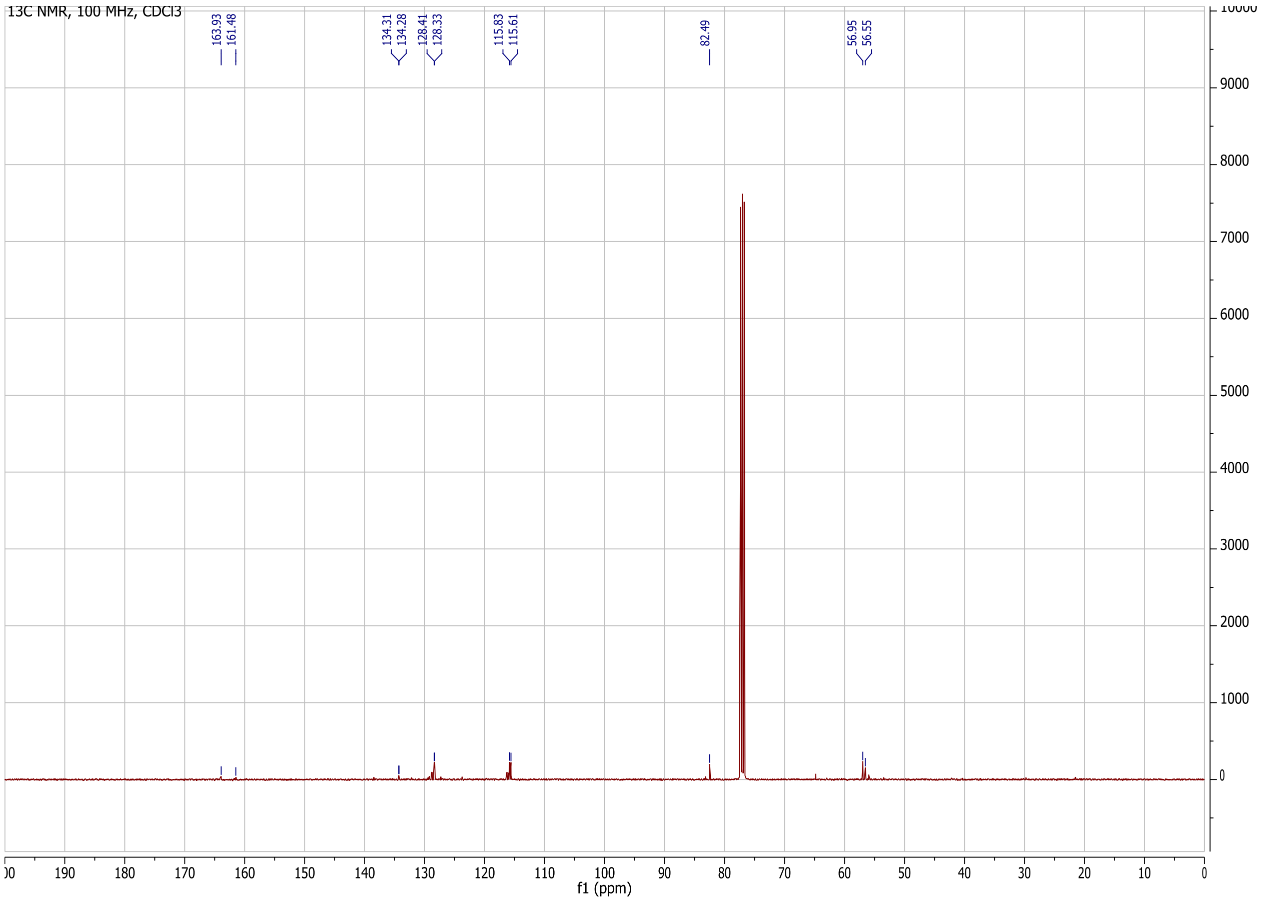

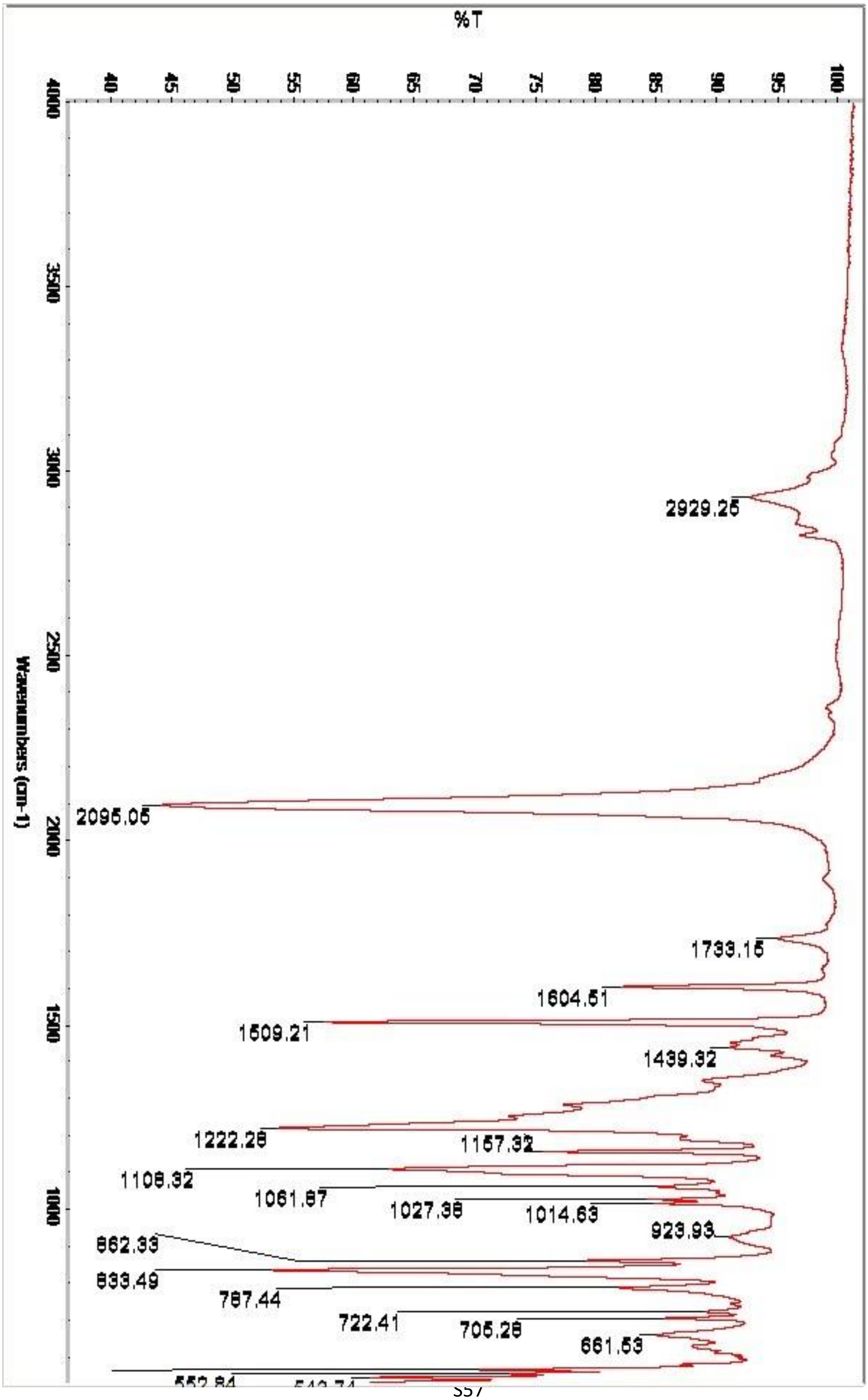
%T
Wavenumbers (cm-1)
2929.25
2095.05
1733.15
1604.51
1509.21
1439.32
1222.28
1157.32
1106.32
1061.87
1027.38
1014.63
923.93
862.33
833.49
787.44
722.41
705.28
661.53

Data for 1-(2-azido-1-methoxyethyl)-4-chlorobenzene (**3p**)

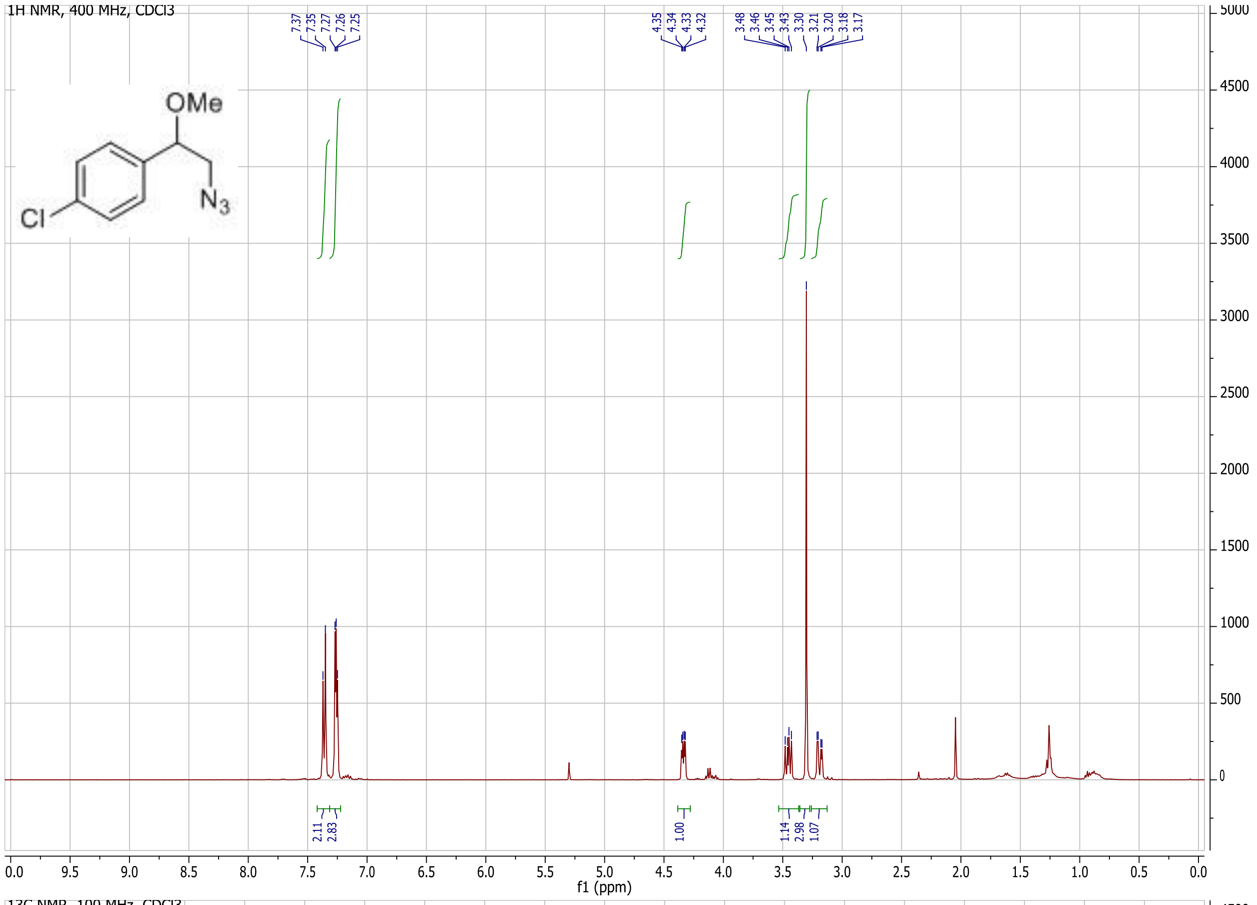

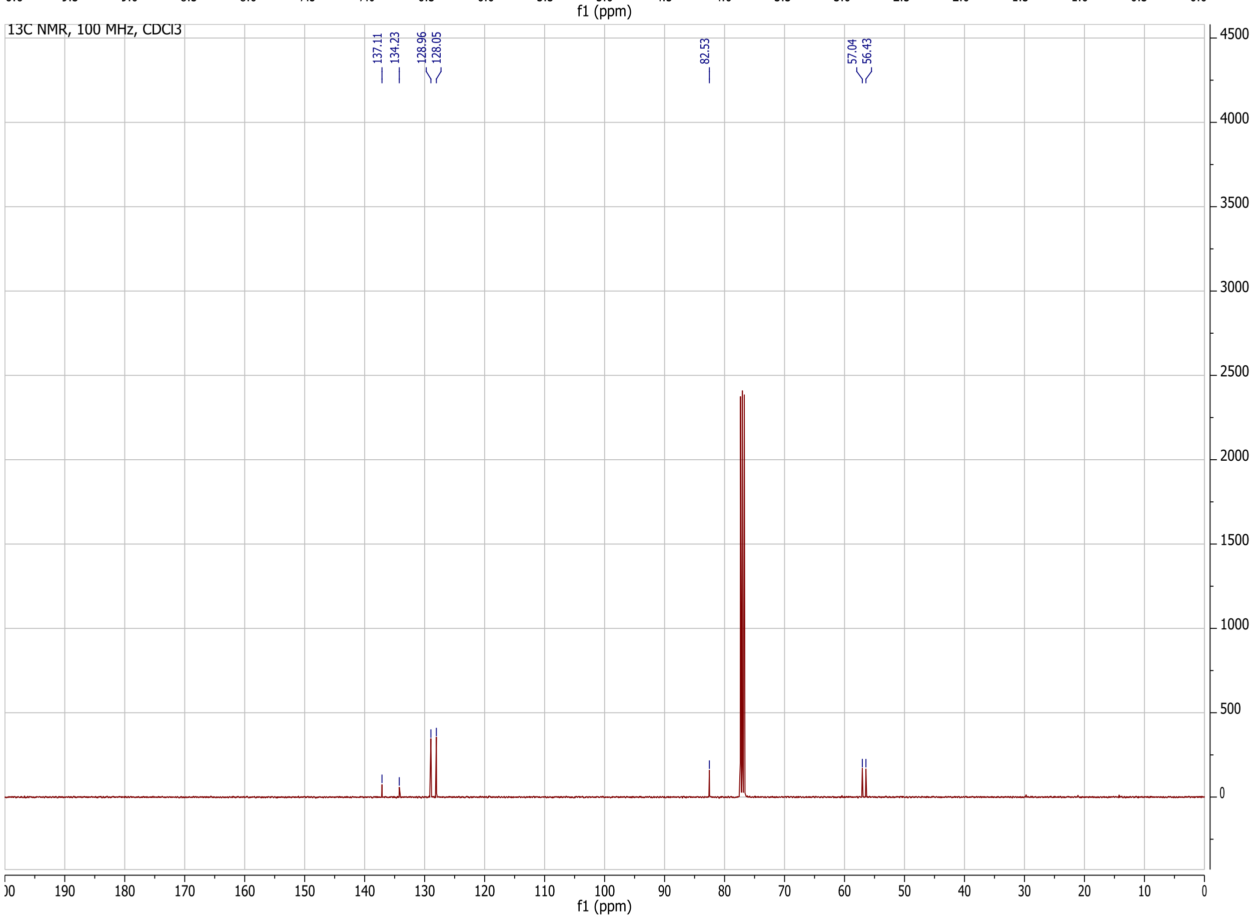

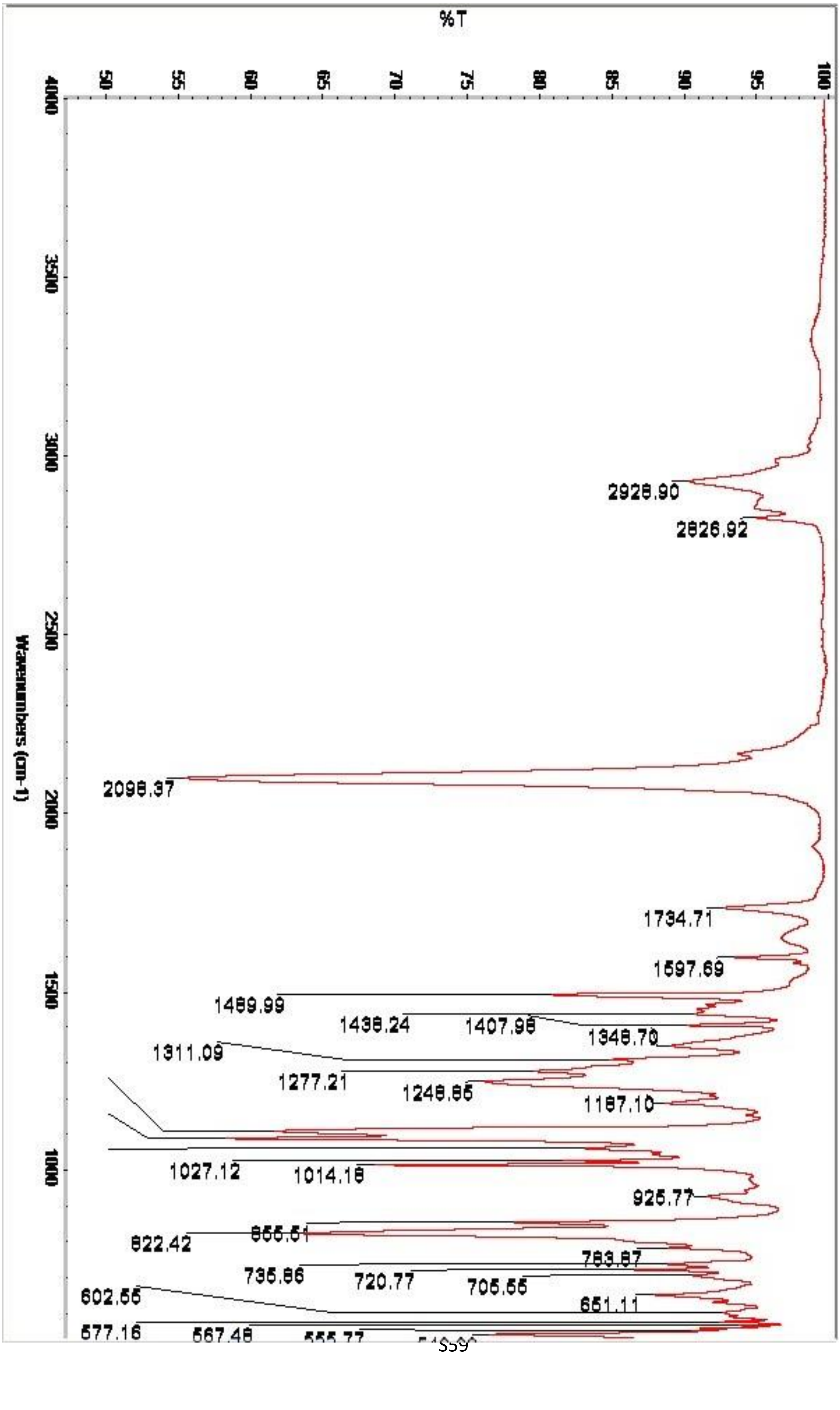
%T
Wavenumbers (cm-1)
2926.90
2826.92
2096.37
1734.71
1597.69
1469.99
1436.24
1407.96
1346.70
1311.09
1277.21
1246.85
1167.10
1027.12
1014.16
925.77
855.51
822.42
763.87
735.86
720.77
705.55
651.11
602.55
577.16
567.48
555.77

Data for 2-(2-azido-1-methoxyethyl)benzothiophene (**3q**)

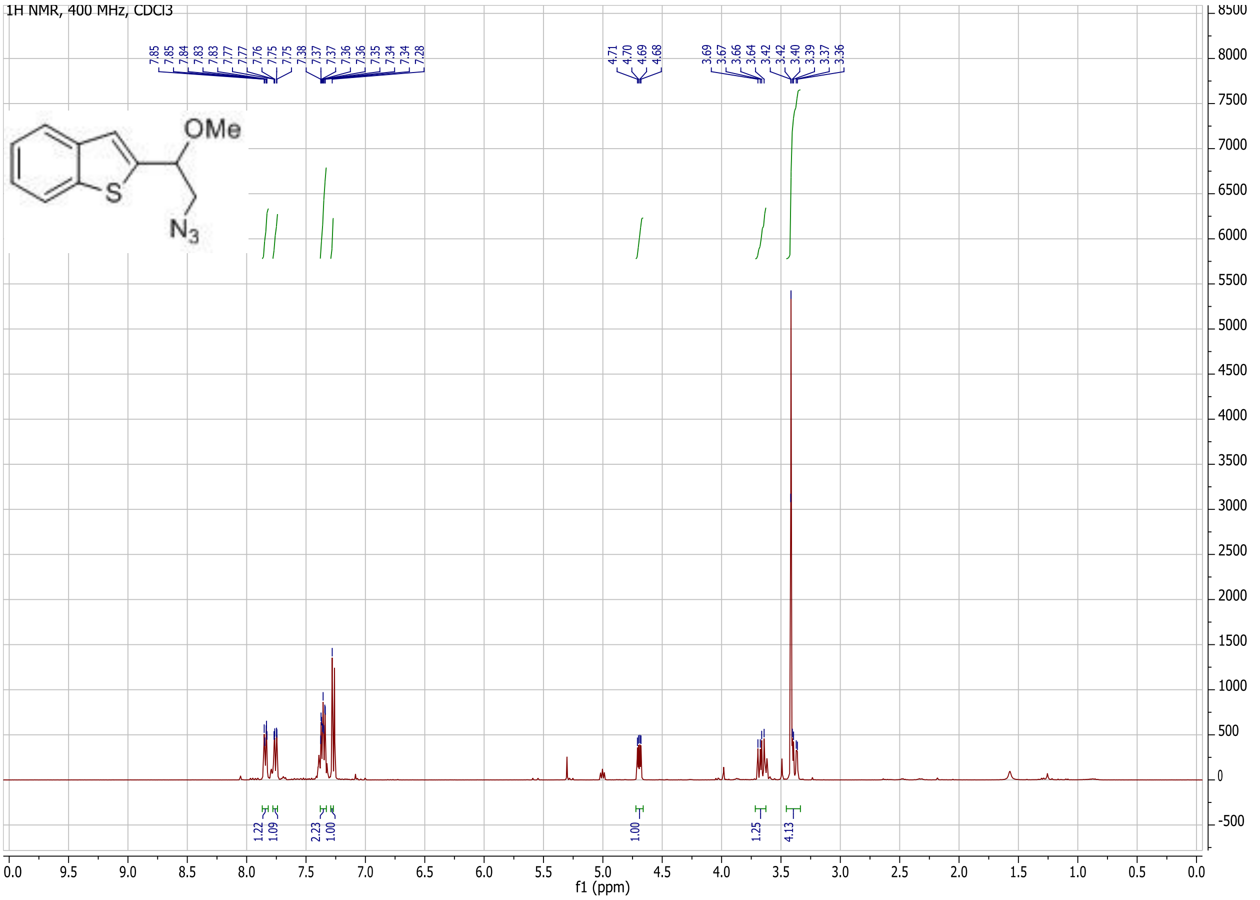

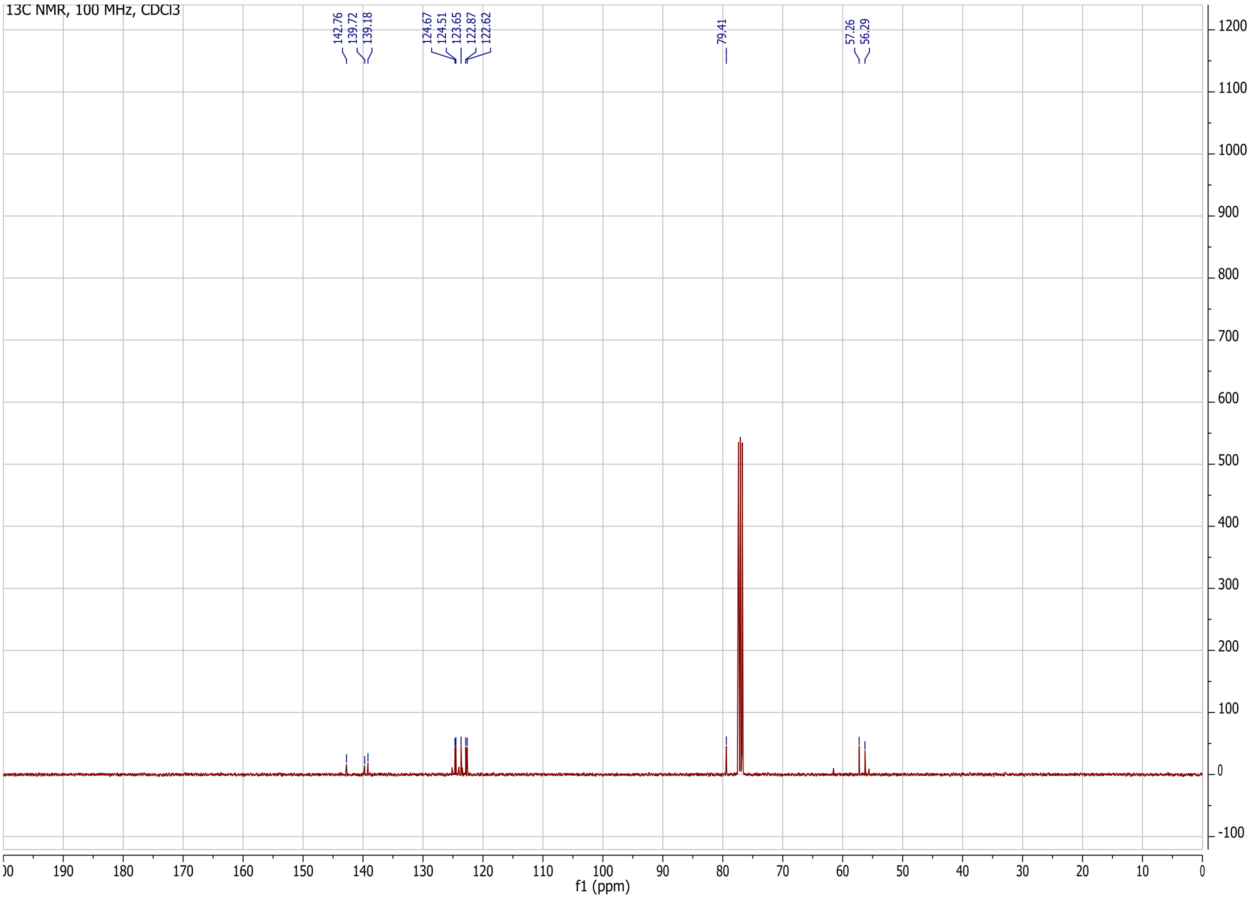

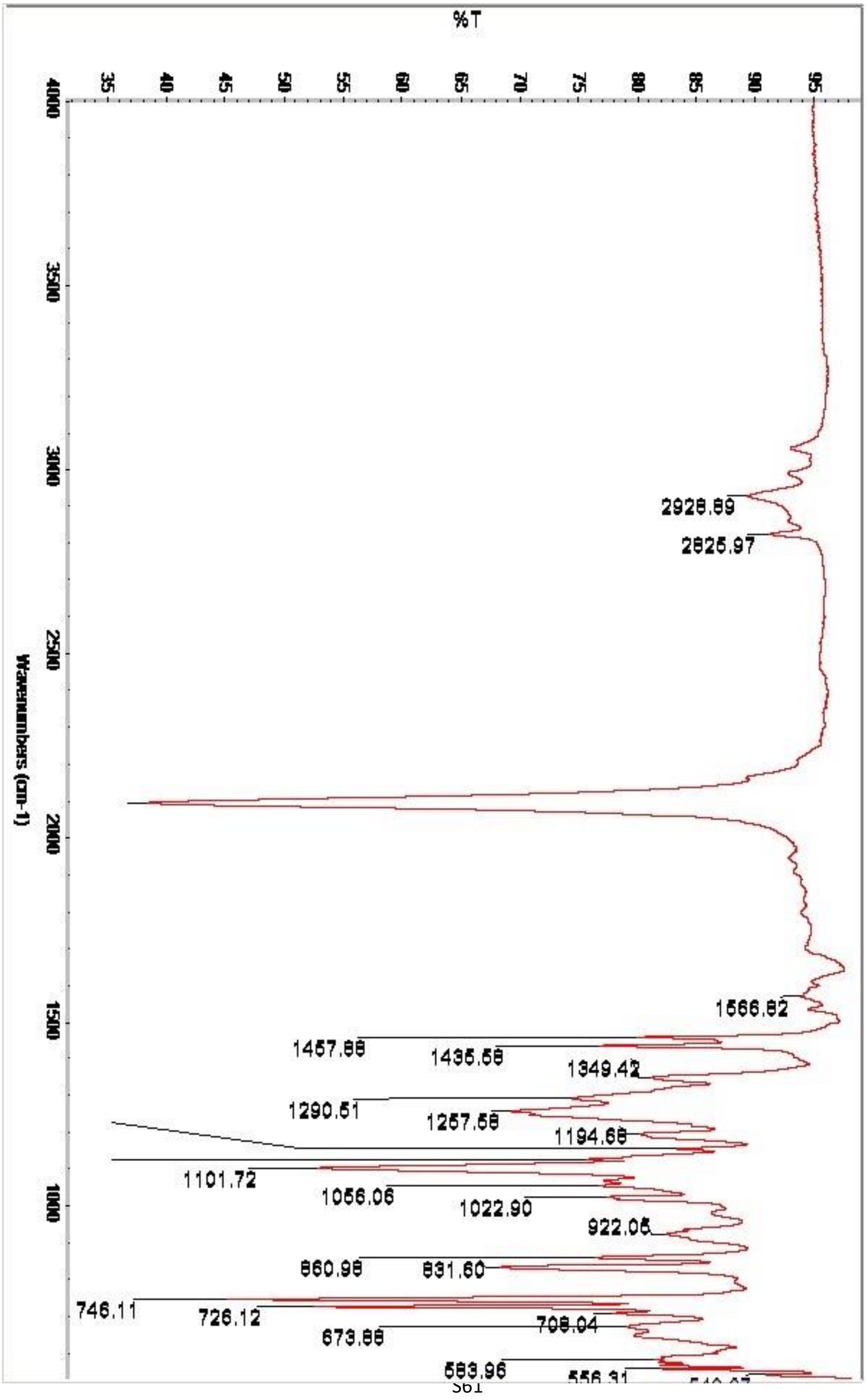
%T
Wavenumbers (cm-1)
2926.89
2825.97
1566.82
1457.88
1436.58
1349.42
1290.51
1257.58
1194.68
1101.72
1056.06
1022.90
922.05
860.98
831.60
746.11
726.12
708.04
673.88
583.96
556.31

Data for 2-(2-azido-1-methoxyethyl)benzofuran (**3r**)

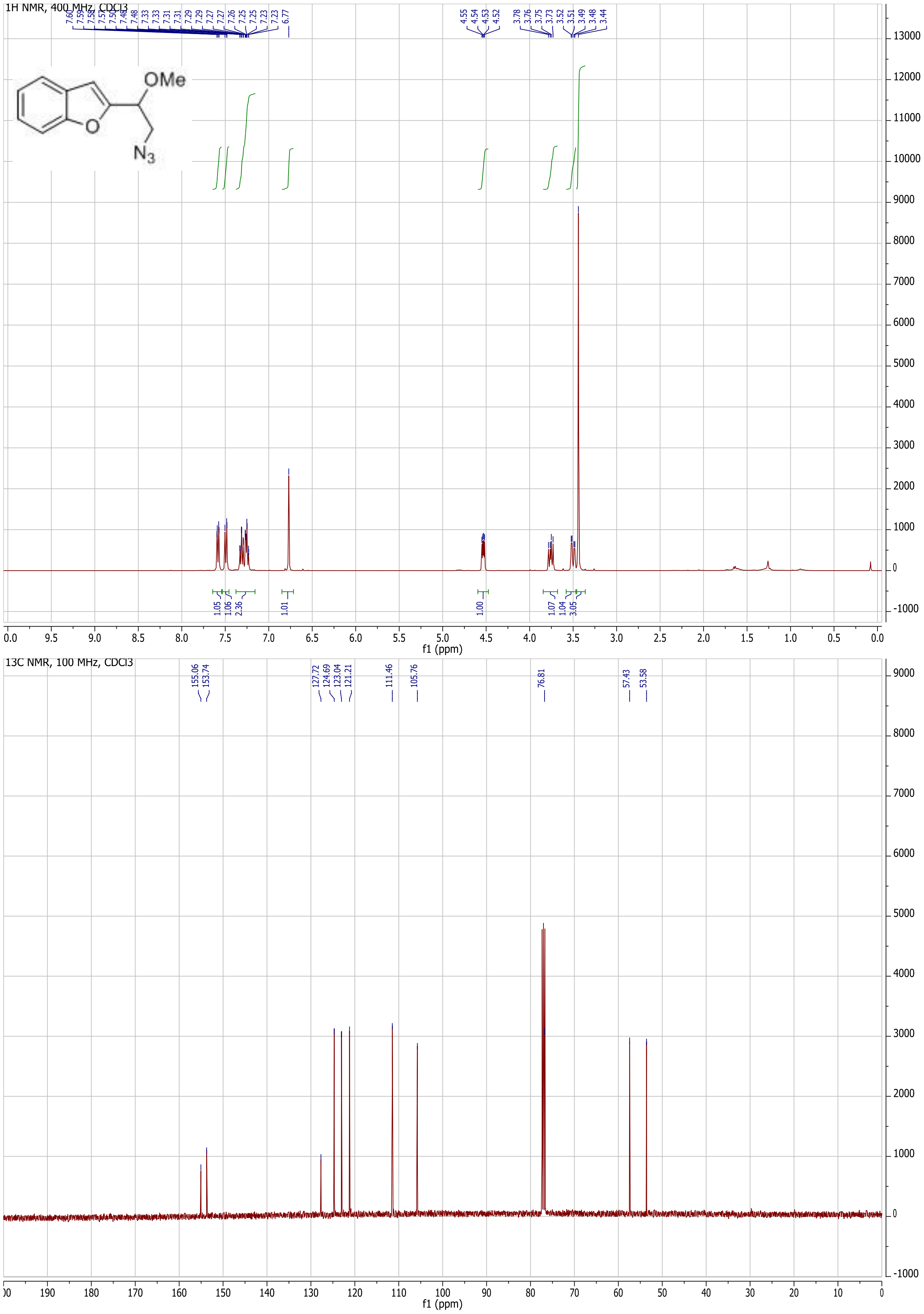

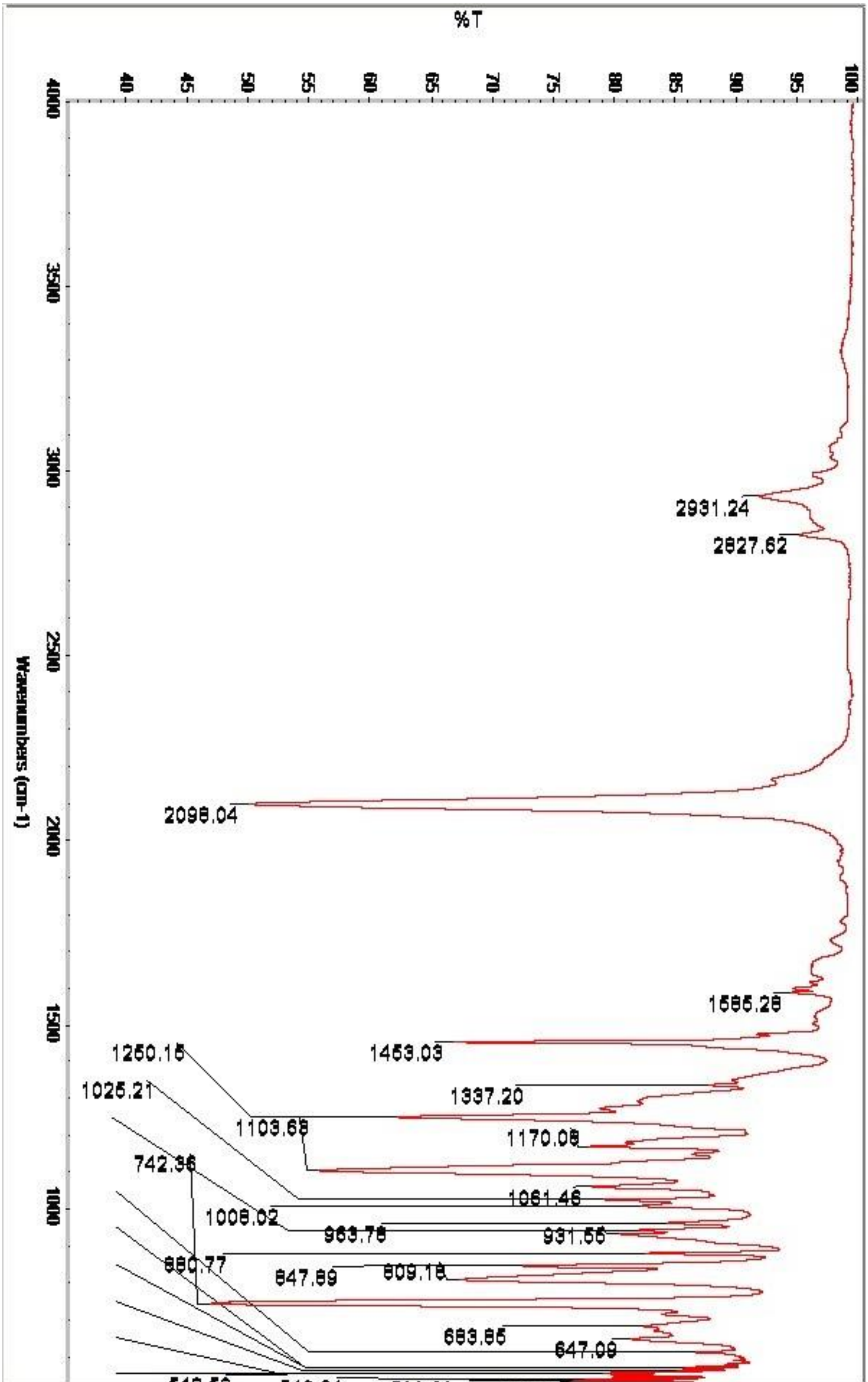
%T
Wavenumbers (cm-1)
2931.24
2827.62
2096.04
1585.28
1453.03
1250.15
1337.20
1025.21
1103.68
1170.08
742.36
1061.46
1006.02
963.76
931.55
660.77
847.89
809.18
683.85
647.09

Data for 1-(2-azido-1-methoxyethyl)-2-(prop-2-yn-1-yloxy)benzene (**3s**)

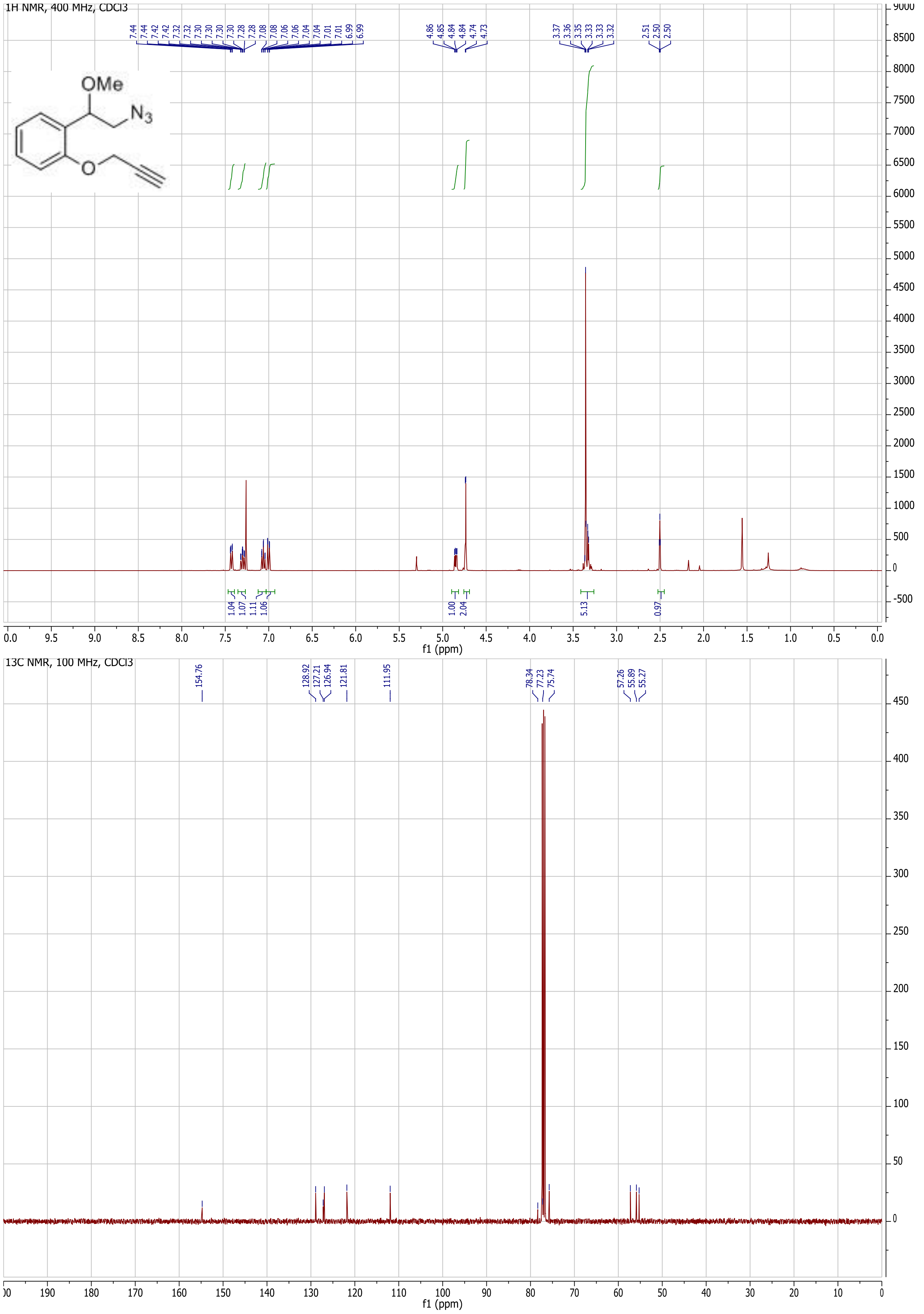

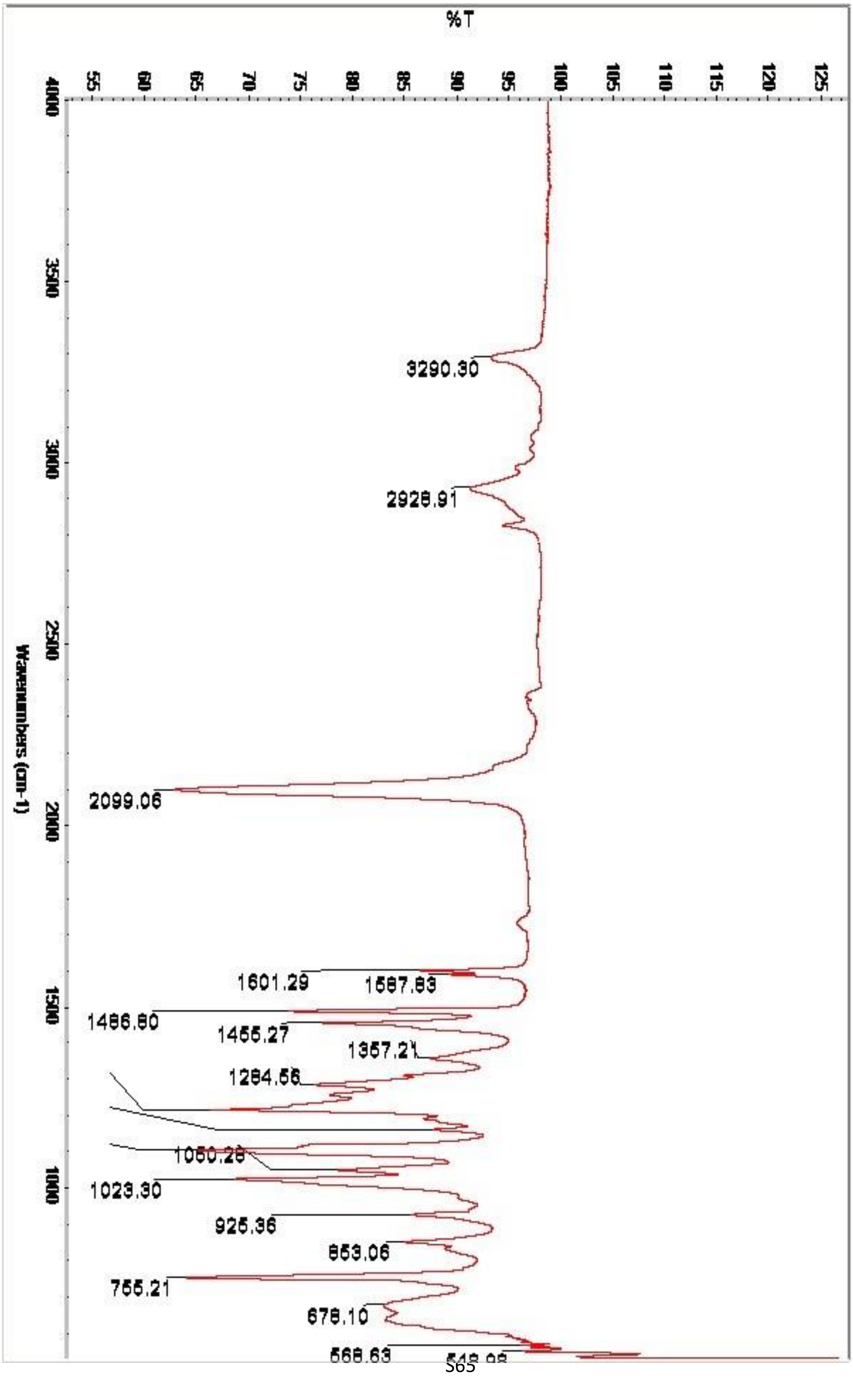
%T
55
60
65
70
75
80
85
90
95
100
105
110
115
120
125
4000
3500
3000
2500
2000
1500
1000
Wavenumbers (cm-1)
3290.30
2926.91
2099.06
1601.29
1587.83
1486.80
1455.27
1357.21
1264.56
1050.28
1023.30
925.36
853.06
755.21
676.10
568.63

Data for 1-(1-azido-2-methoxypropan-2-yl)-4-fluorobenzene (not in table)

1H NMR, 400 MHz, CDCl3

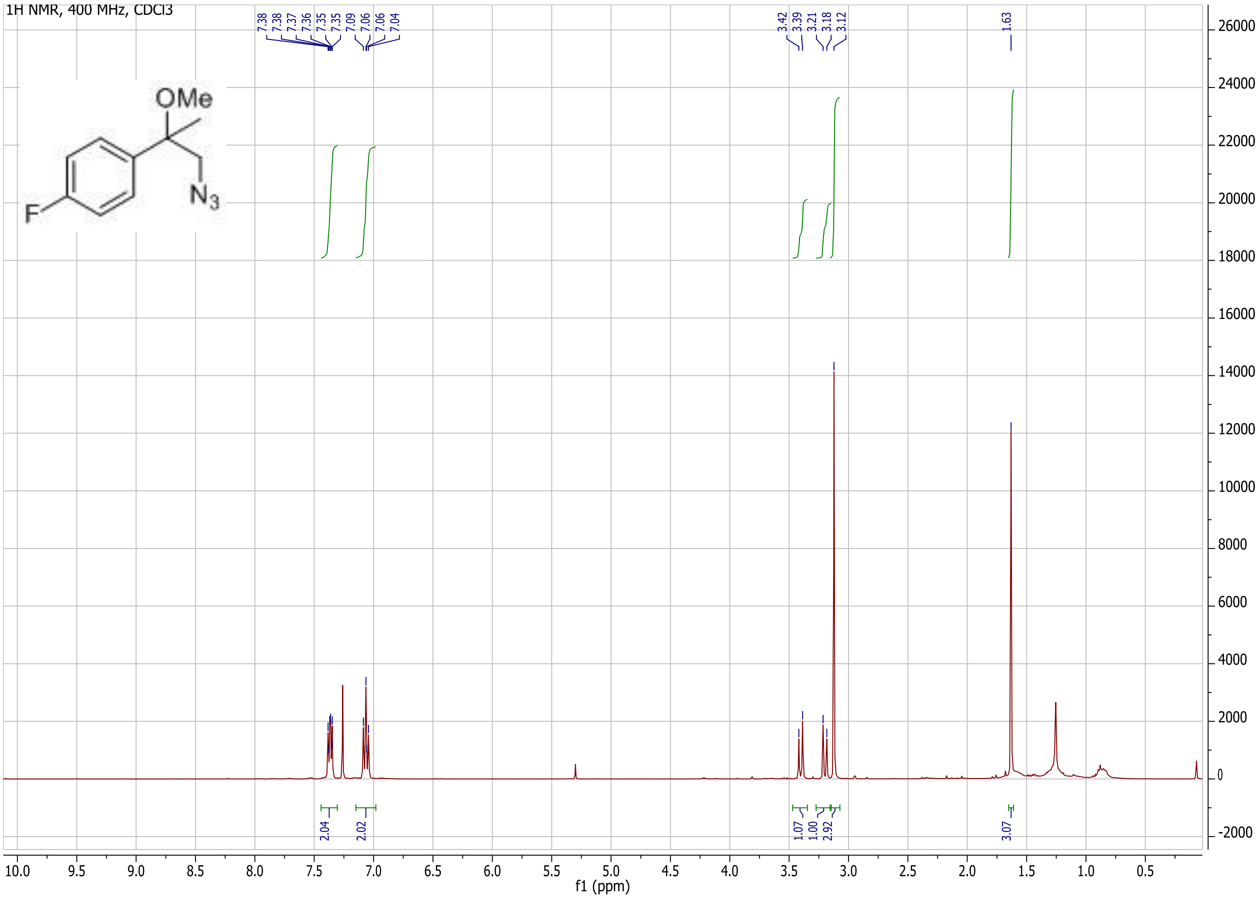

13C NMR, 100 MHz, CDCl3

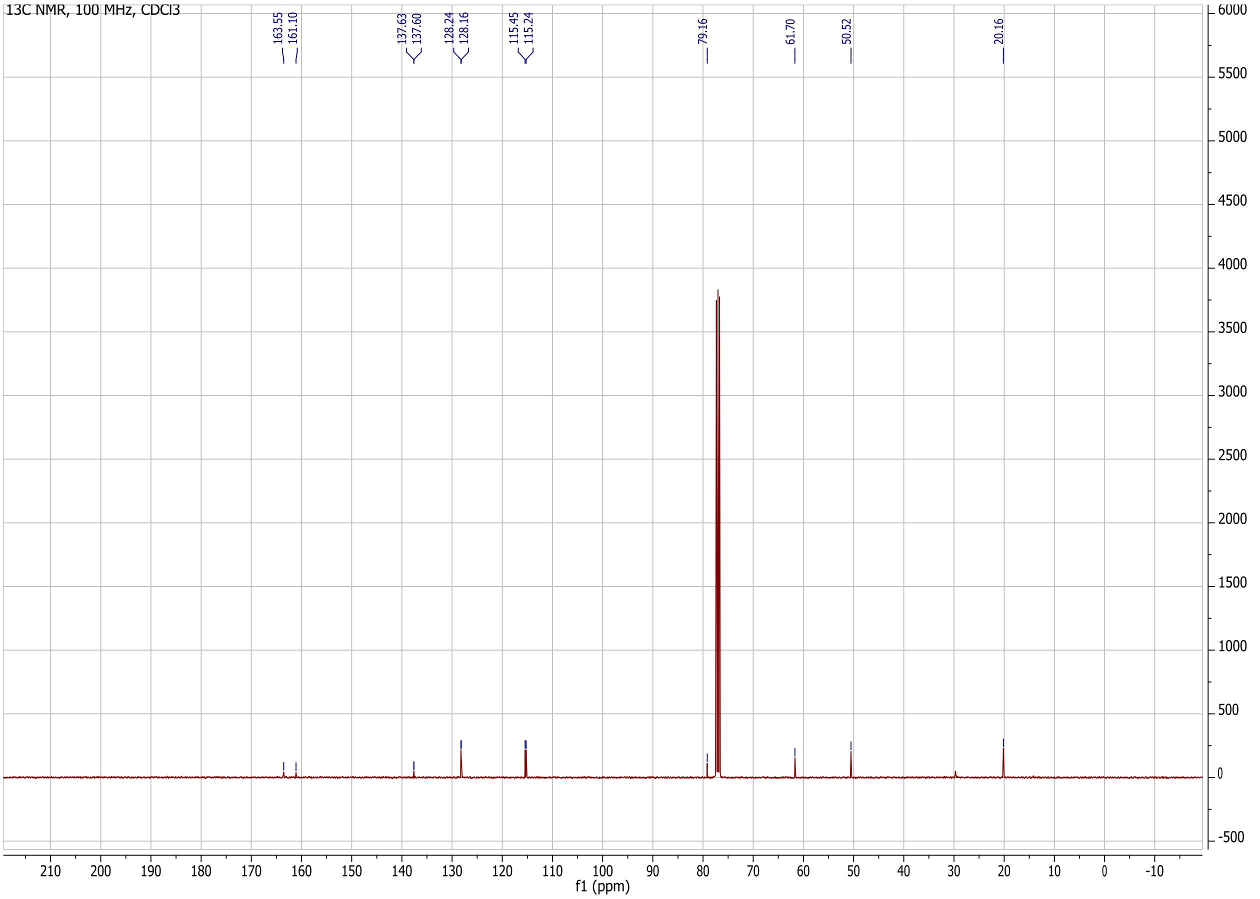

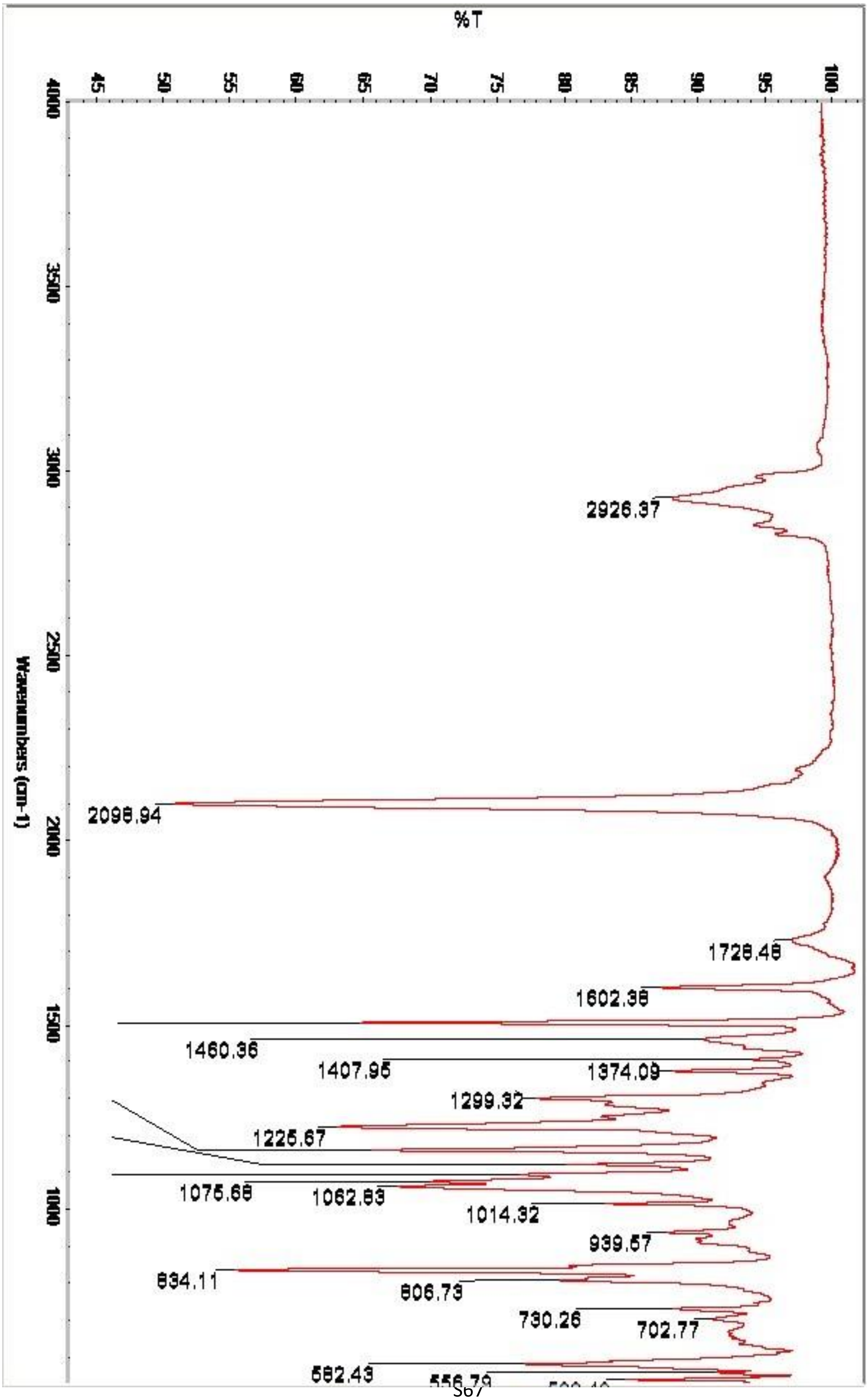
%T
Wavenumbers (cm-1)
2926.37
2098.94
1726.46
1602.38
1460.36
1407.95
1374.09
1299.32
1225.67
1075.68
1062.83
1014.32
939.57
834.11
806.73
730.26
702.77
582.43

Data for 10-methoxy-10,11-dihydro-4H-benzo[*g*][1,2,3]triazolo[5,1-*c*][1,4]oxazocine (**7**)

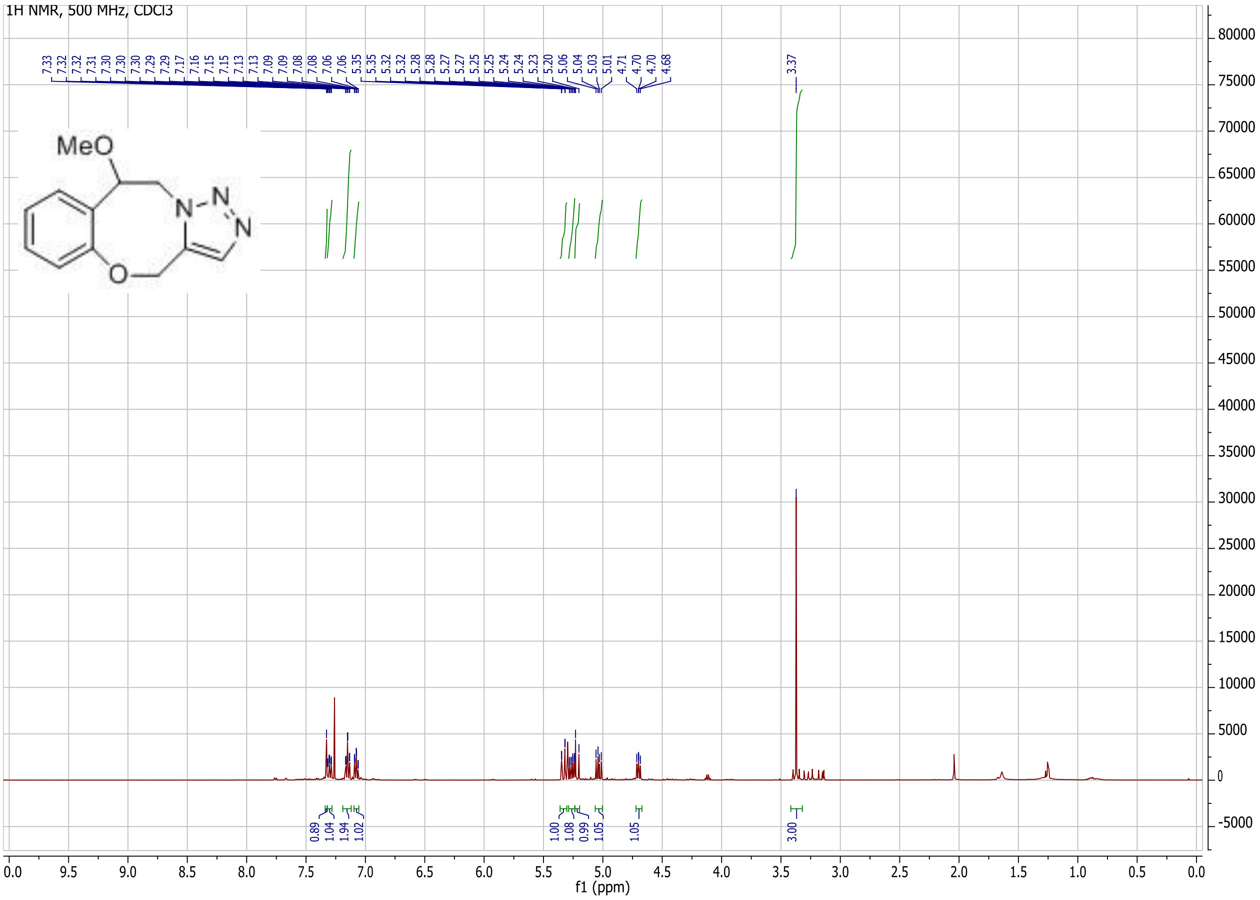

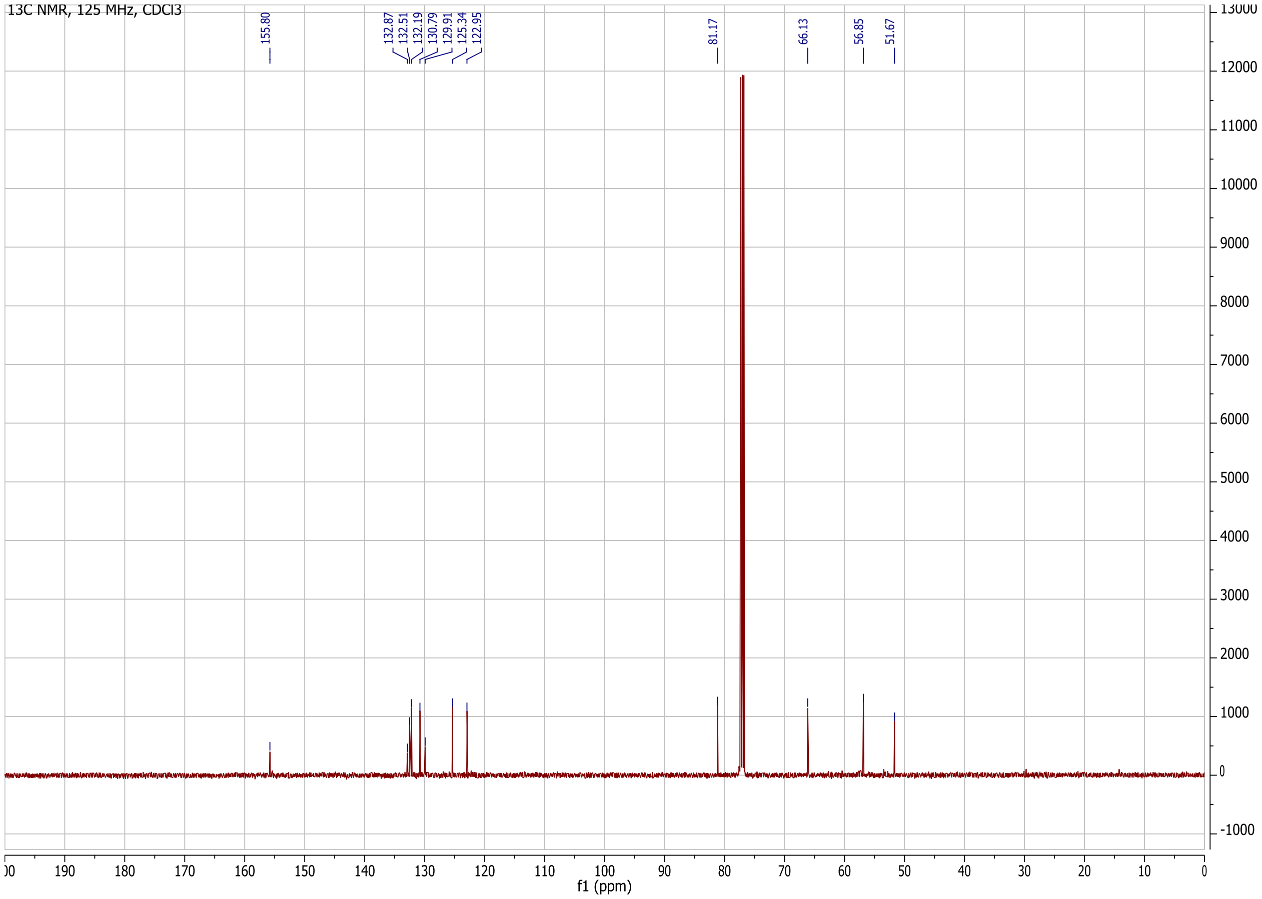

8 ^{1}H NMR, ^{13}C NMR and IR spectra for azido-functionalised compounds 4a, 5 and 6

Data for (1,2-diazidoethyl)benzene (**4a**)

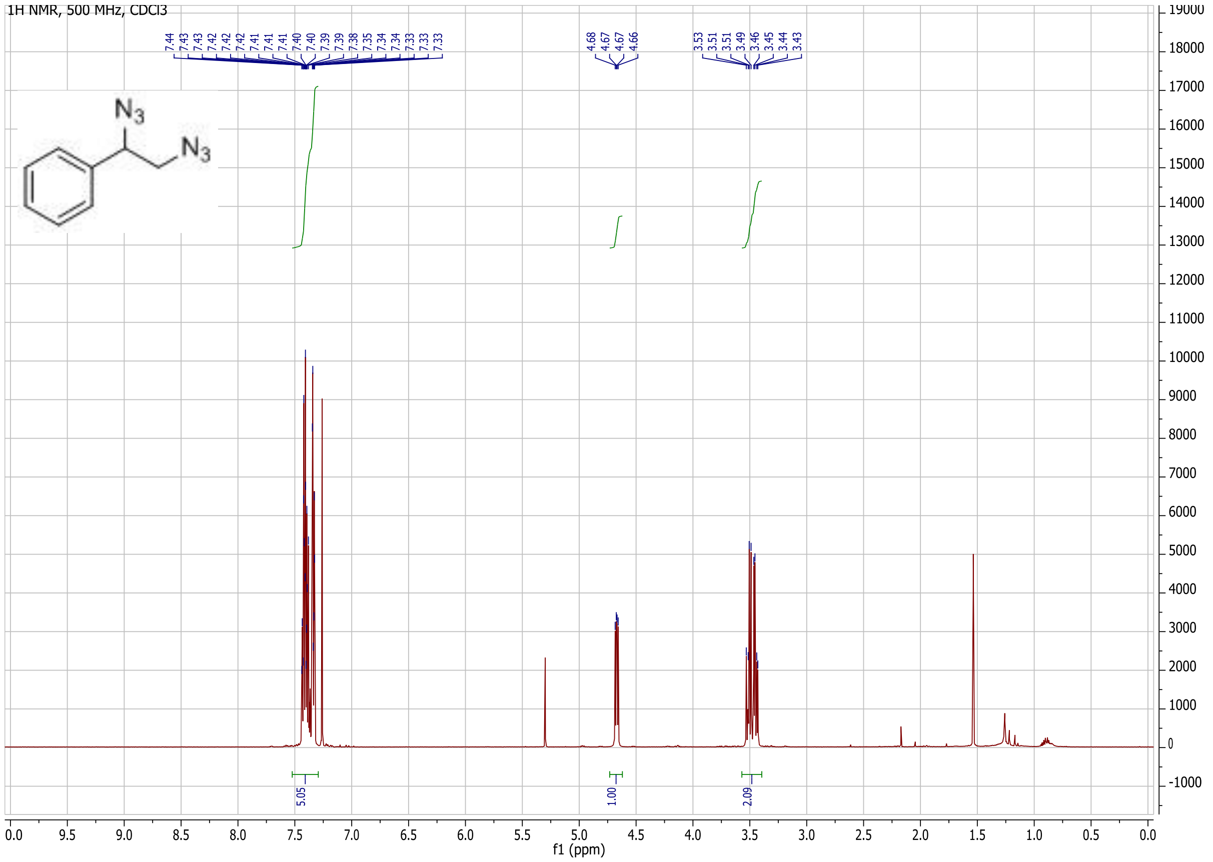

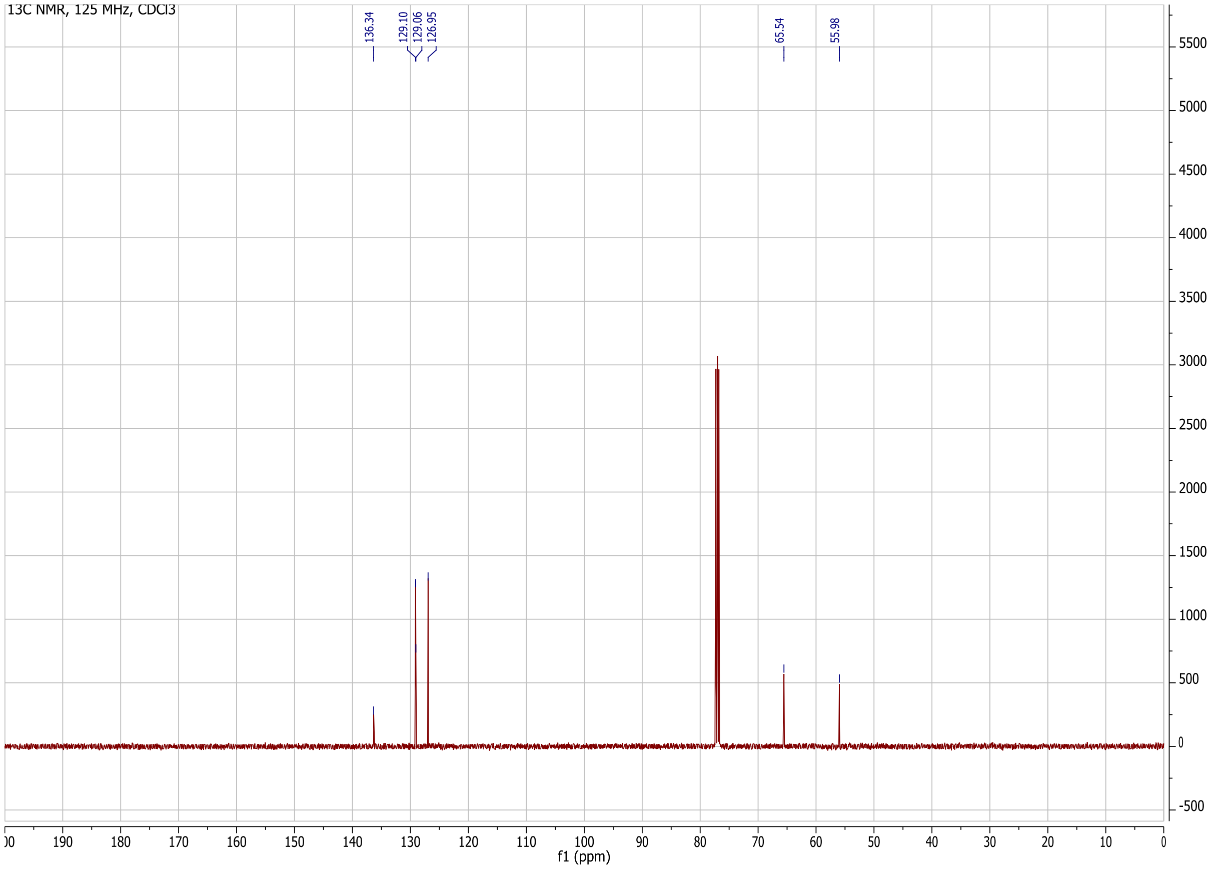

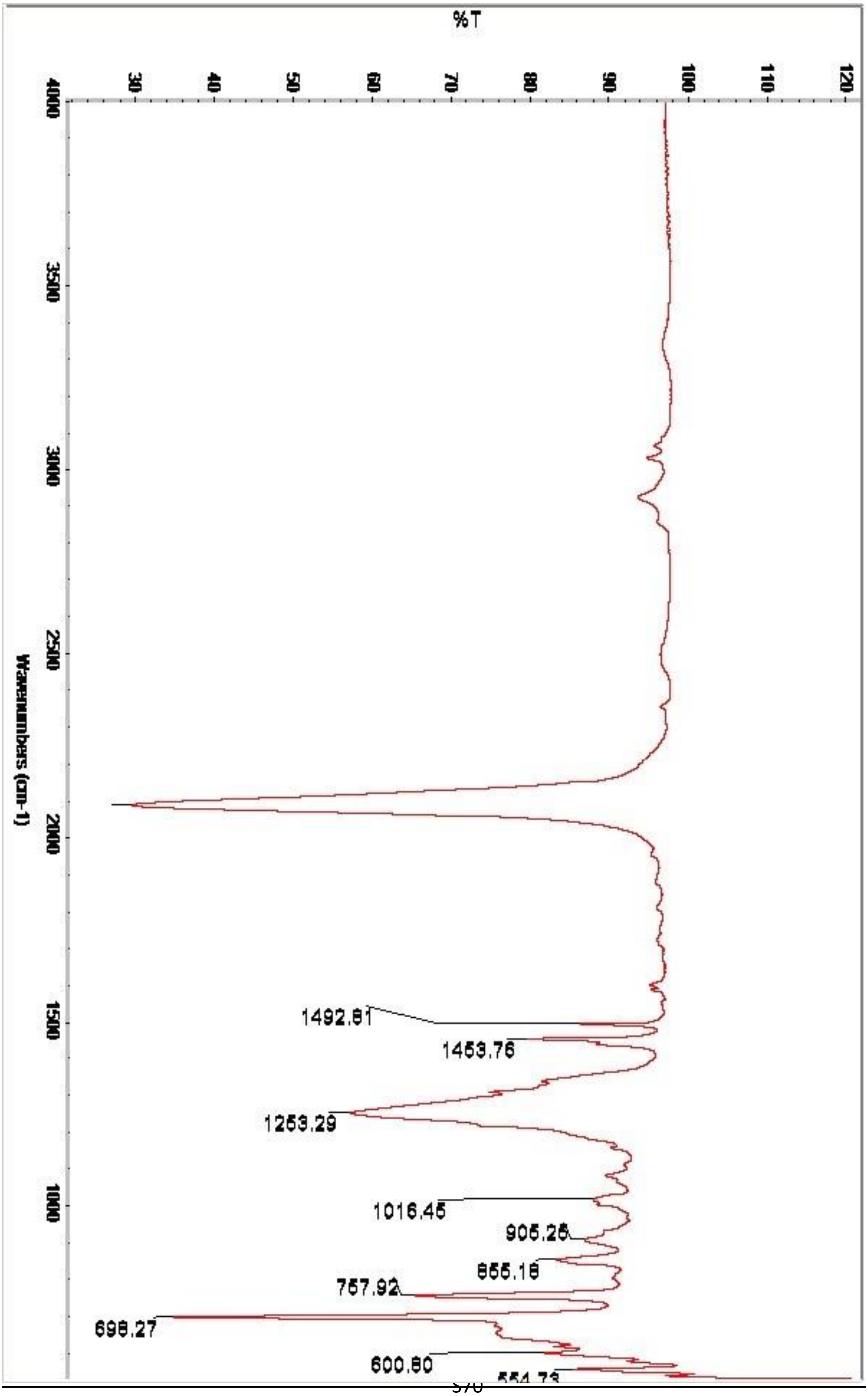
%T
Wavenumbers (cm-1)
1492.81
1453.76
1253.29
1016.45
905.26
855.18
757.92
696.27
600.80
554.73

Data for 2-azido-1-bromo-1-phenylethane (**5**)

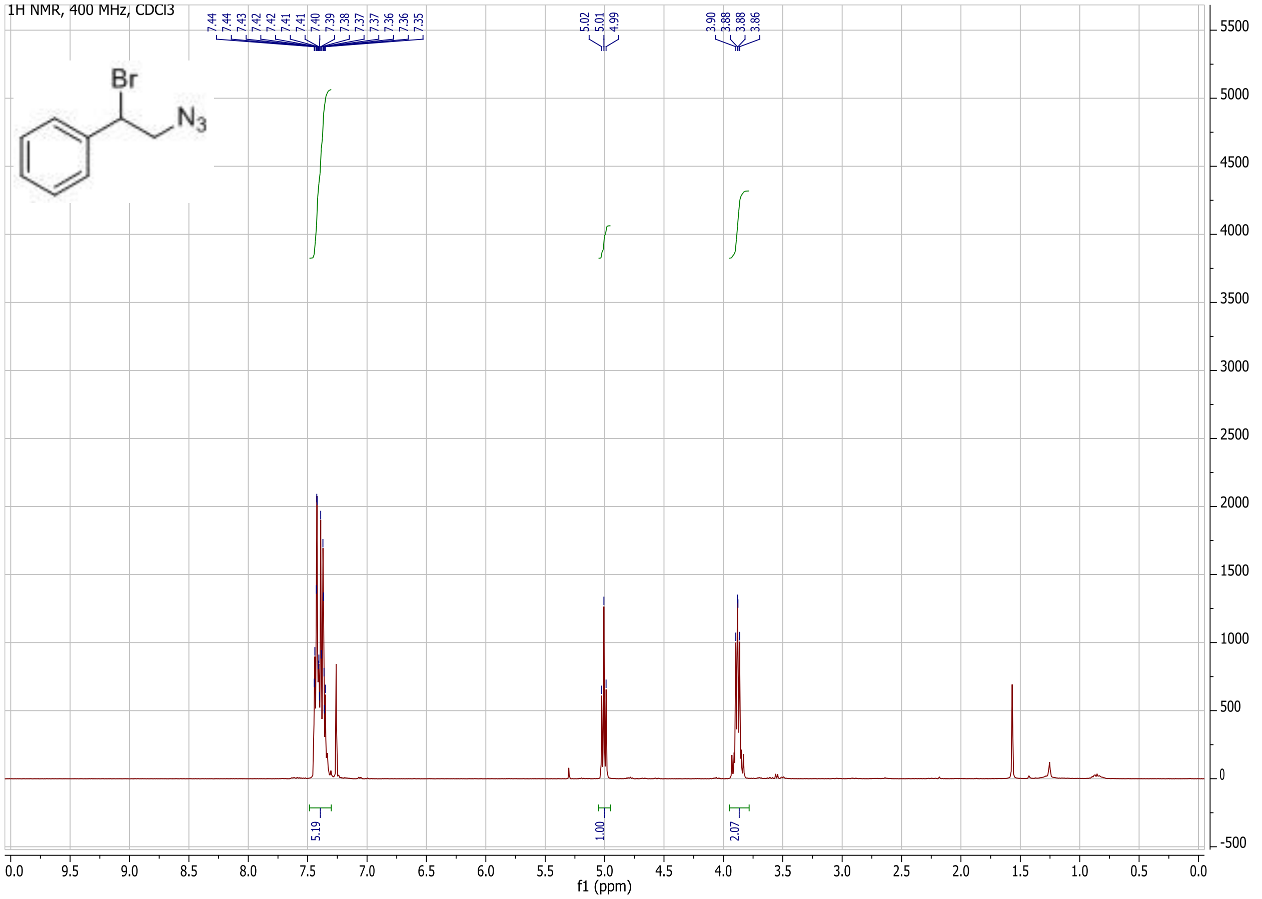

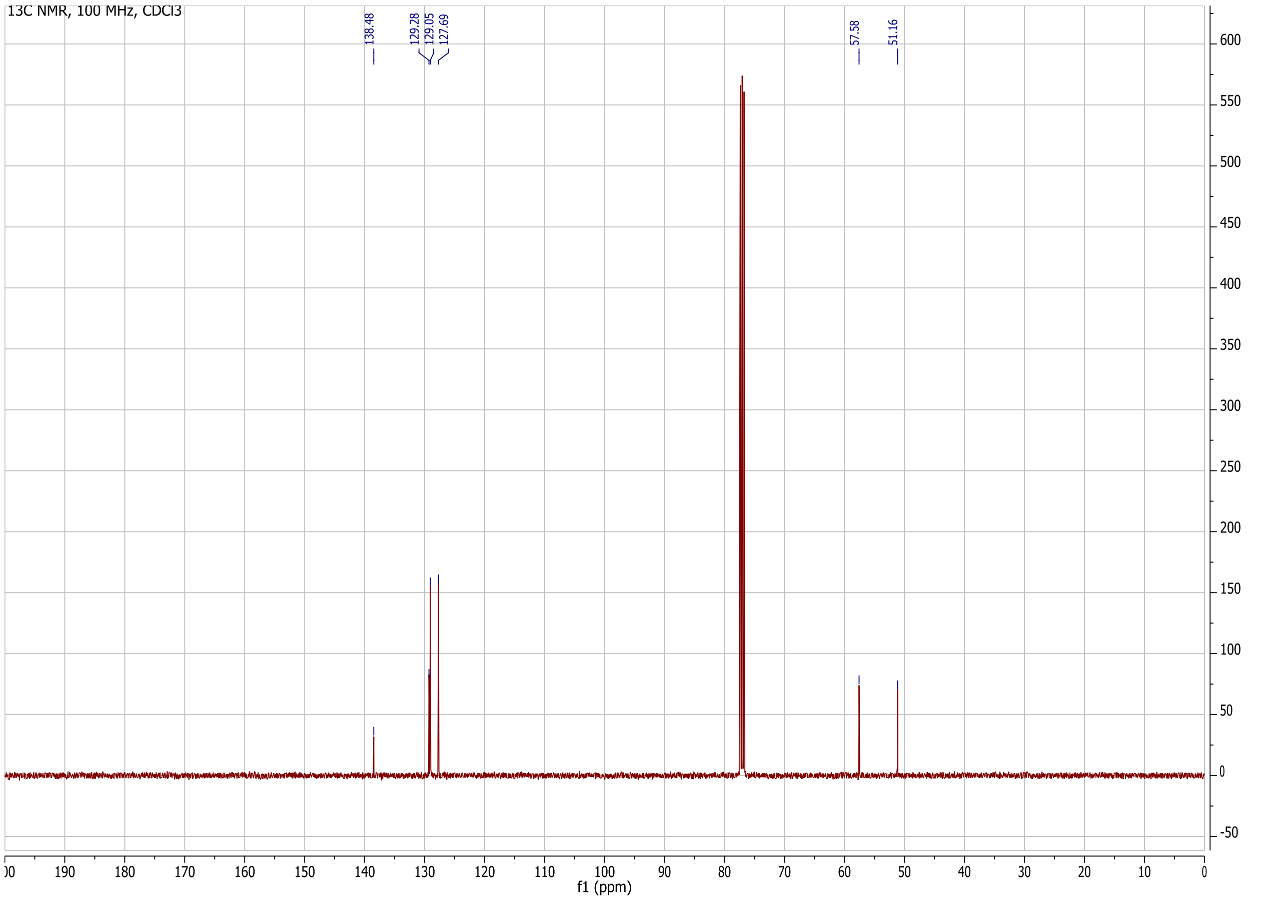

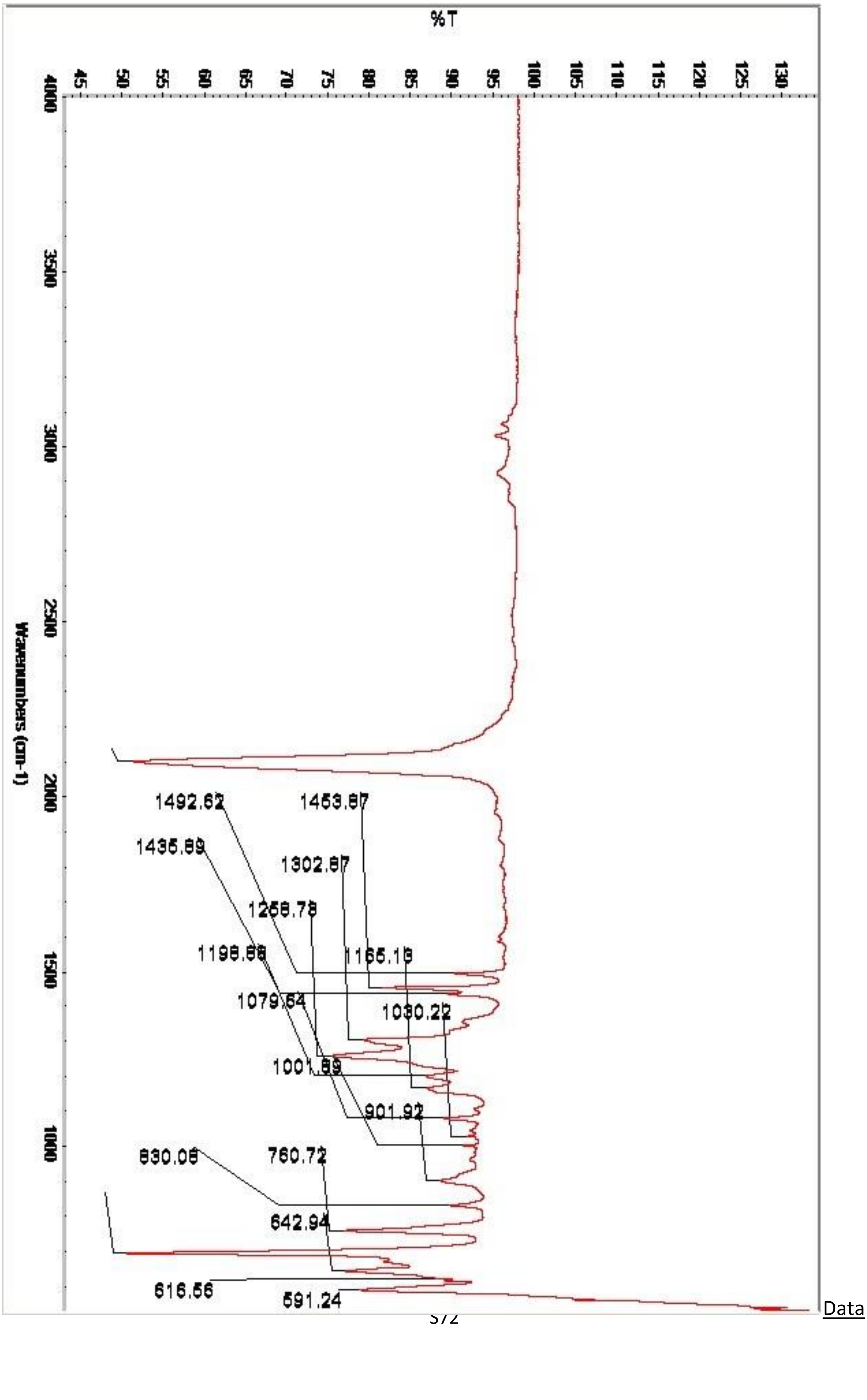
%T
Wavenumbers (cm-1)
1492.62
1453.87
1436.89
1302.87
1258.78
1196.86
1165.13
1079.64
1030.22
1001.89
901.92
830.06
760.72
642.94
616.56
591.24

Data

for (2-azido-1-(2,2,2-trifluoroethoxy)ethyl)benzene (**6**)

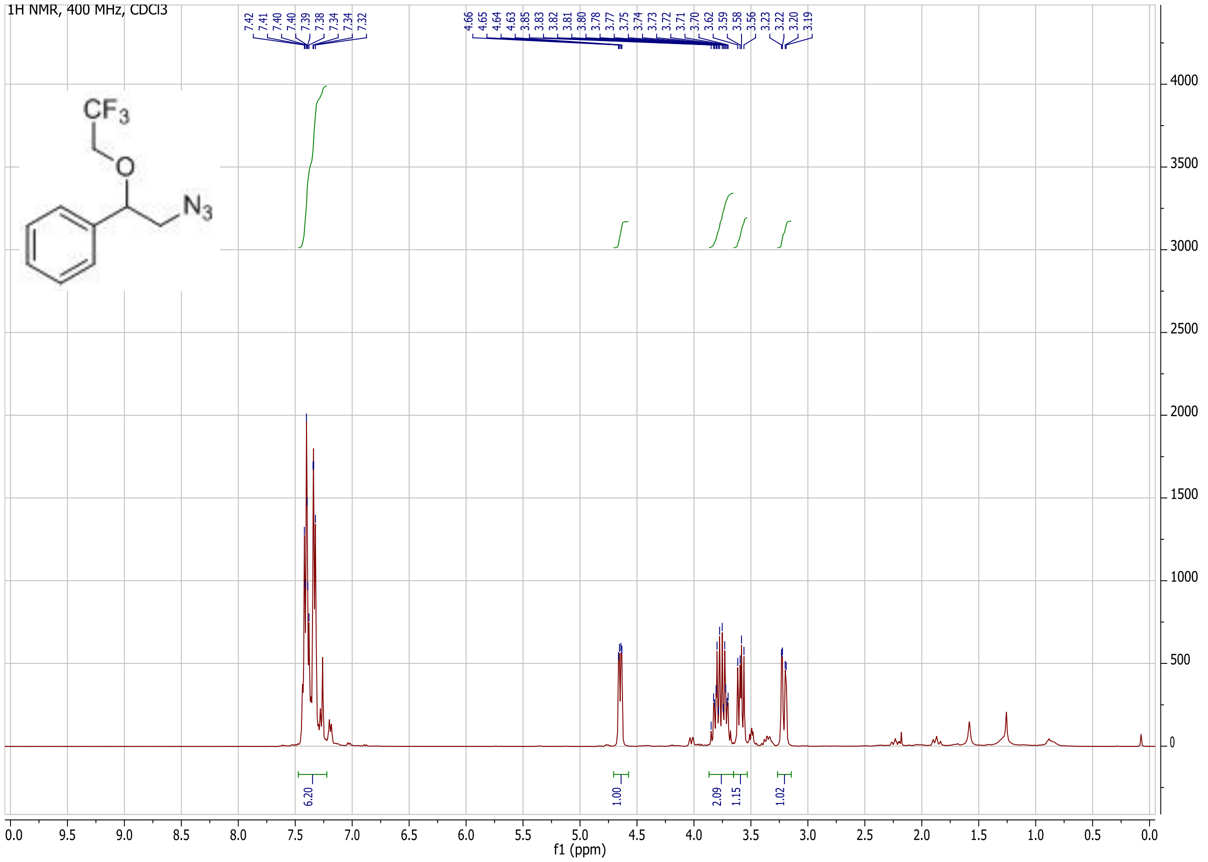

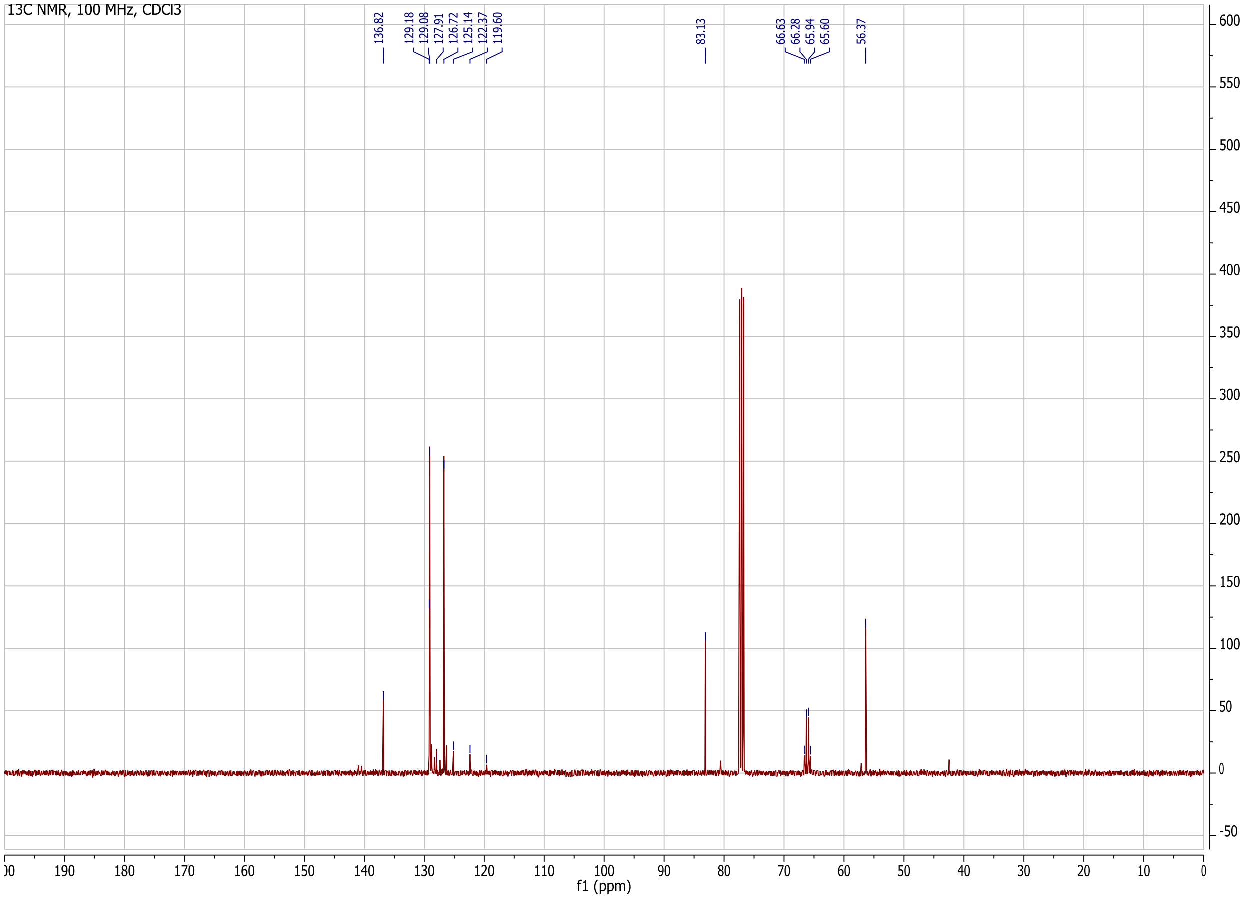

9 ^{1}H NMR, ^{13}C NMR and IR spectra for diazido-functionalised compounds 4a-j

Data for (1,2-diazidoethyl)-1,1-dibenzene (**4b**)

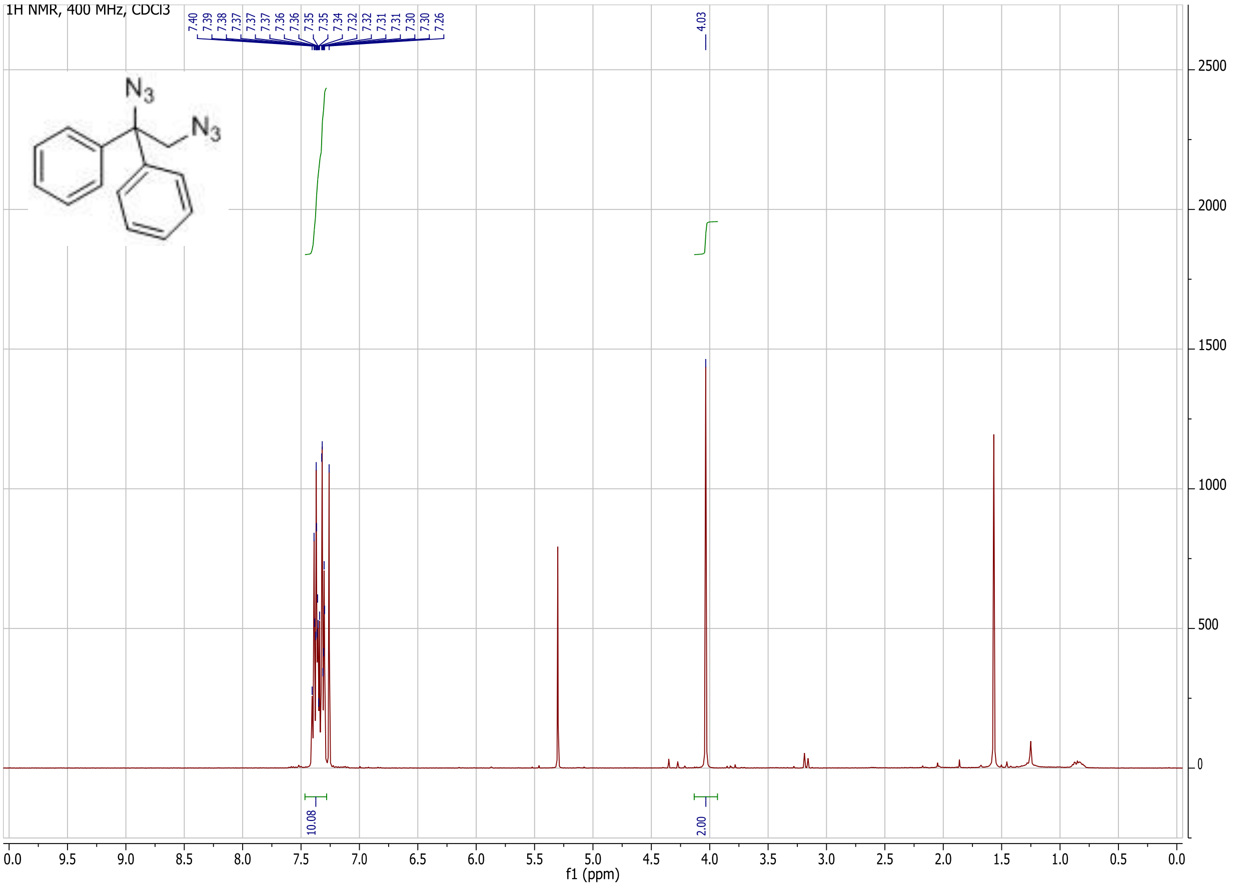

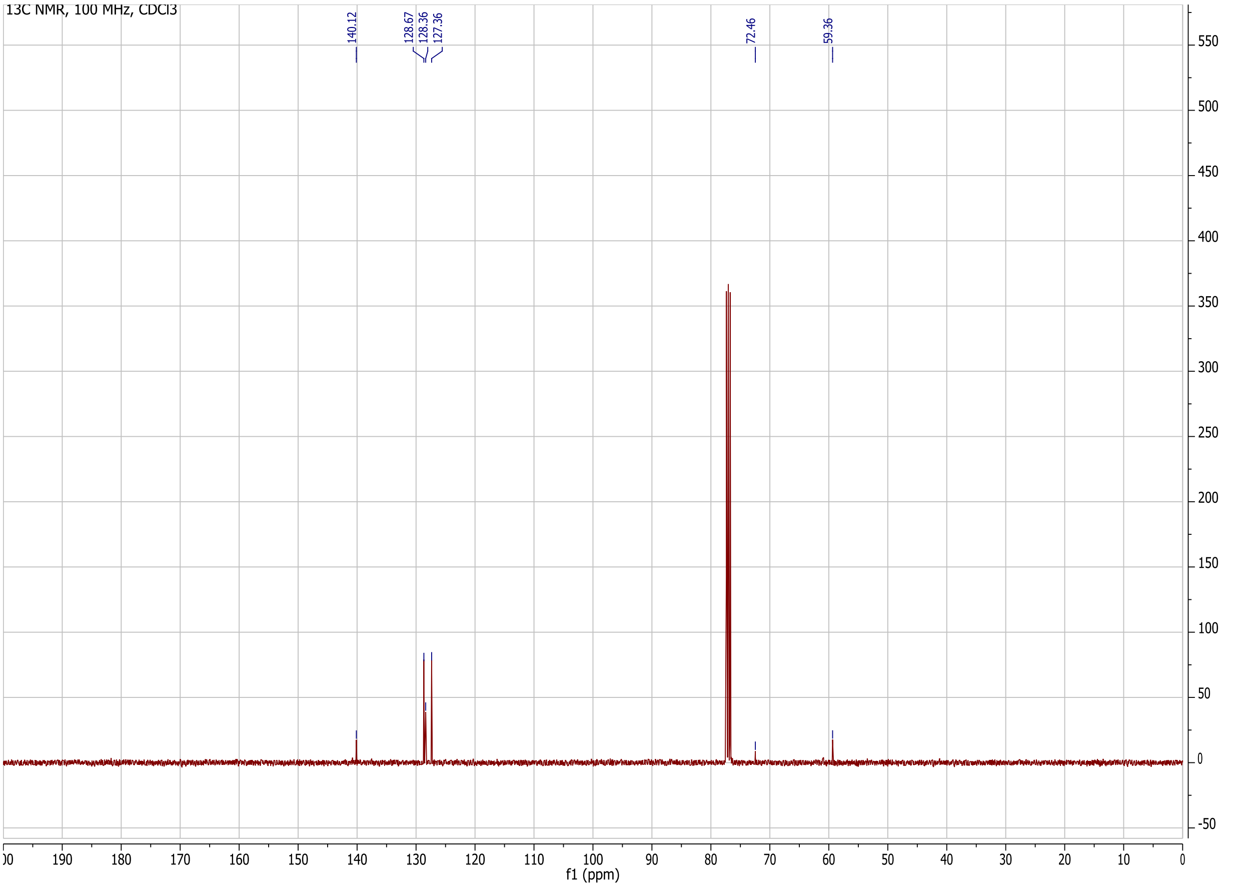

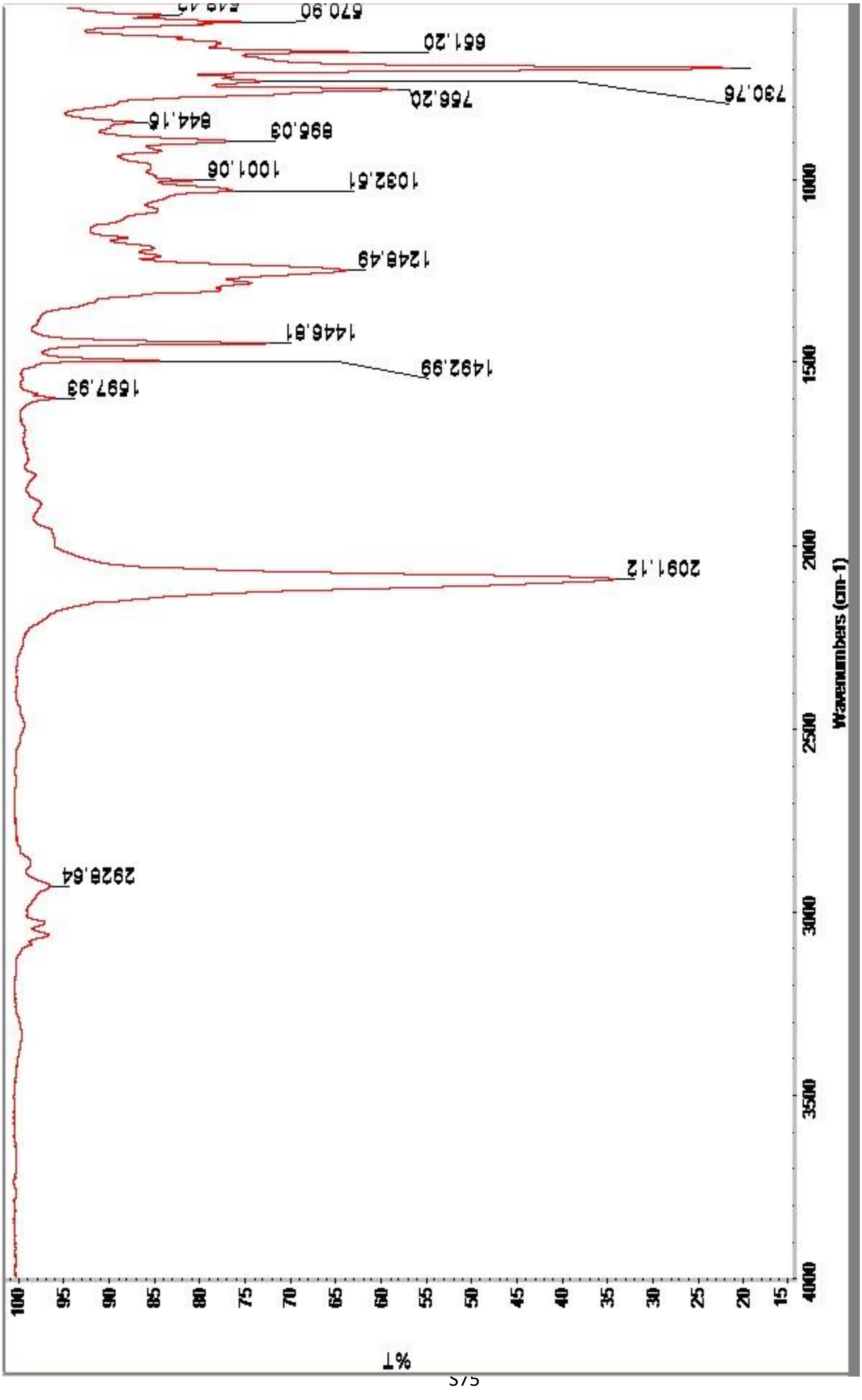

%T
Wavenumbers (cm-1)
2928.64
2091.12
1597.93
1492.99
1446.81
1248.49
1032.51
1001.06
895.03
844.16
756.20
730.76
651.20
670.90

Data for 1,2-diazido-1,2-diphenylethane (**4c**)

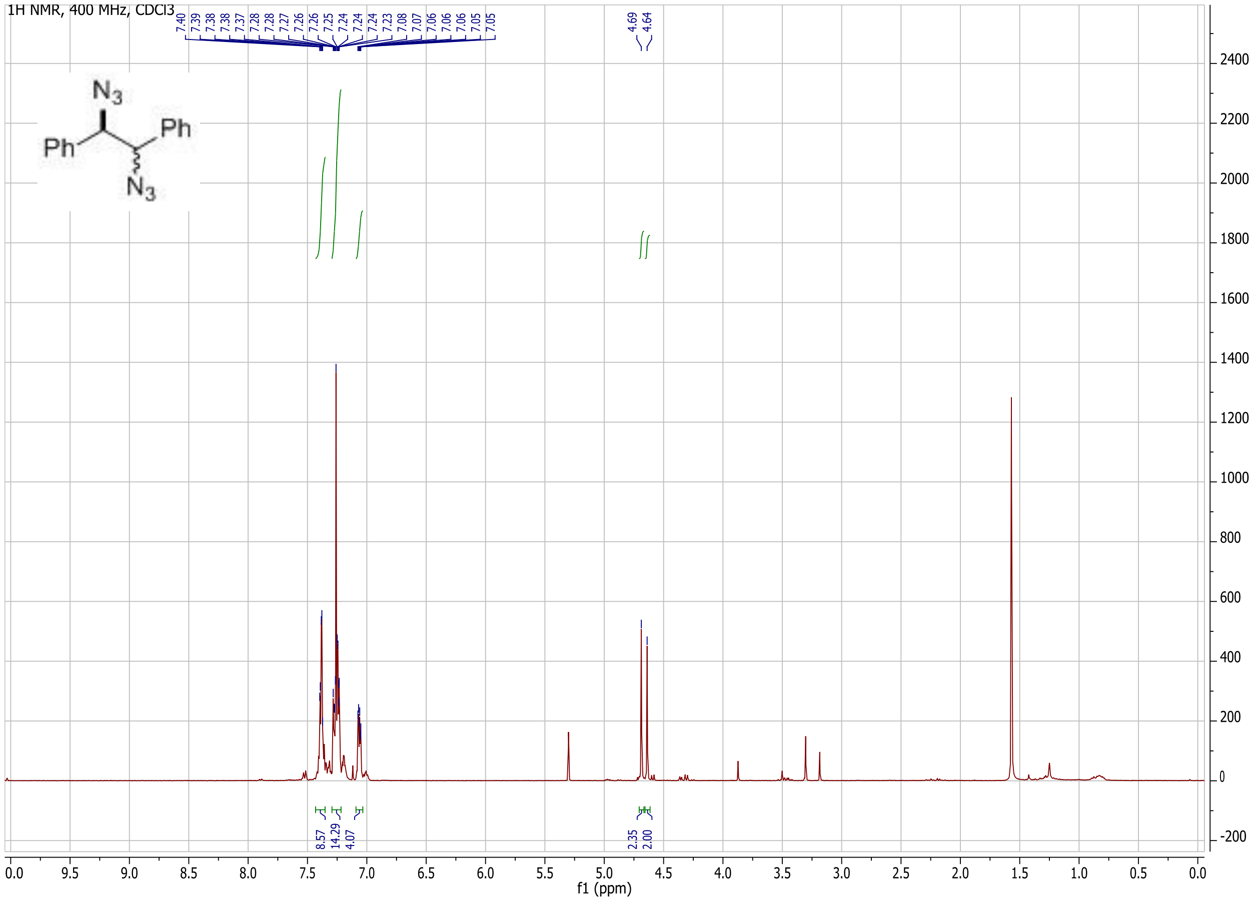

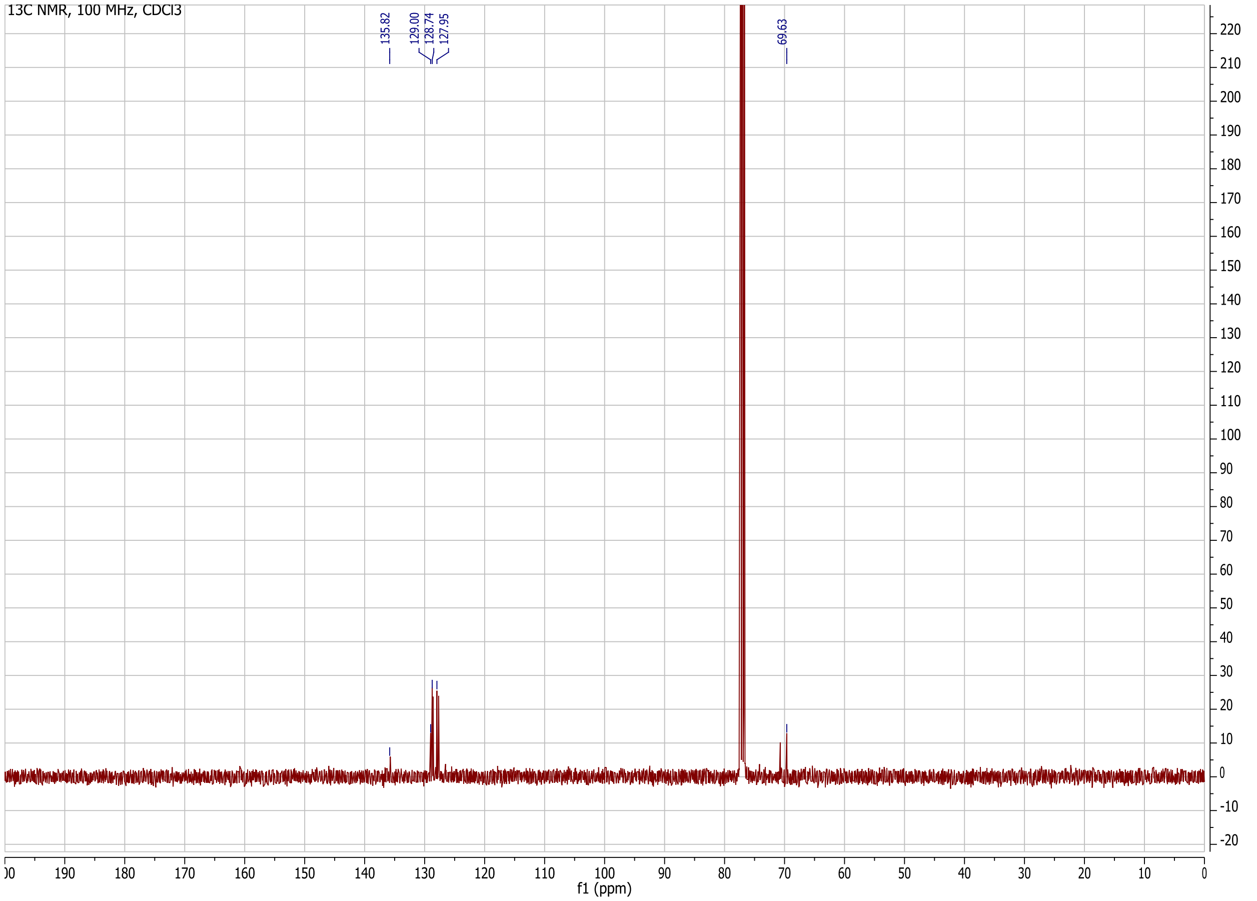

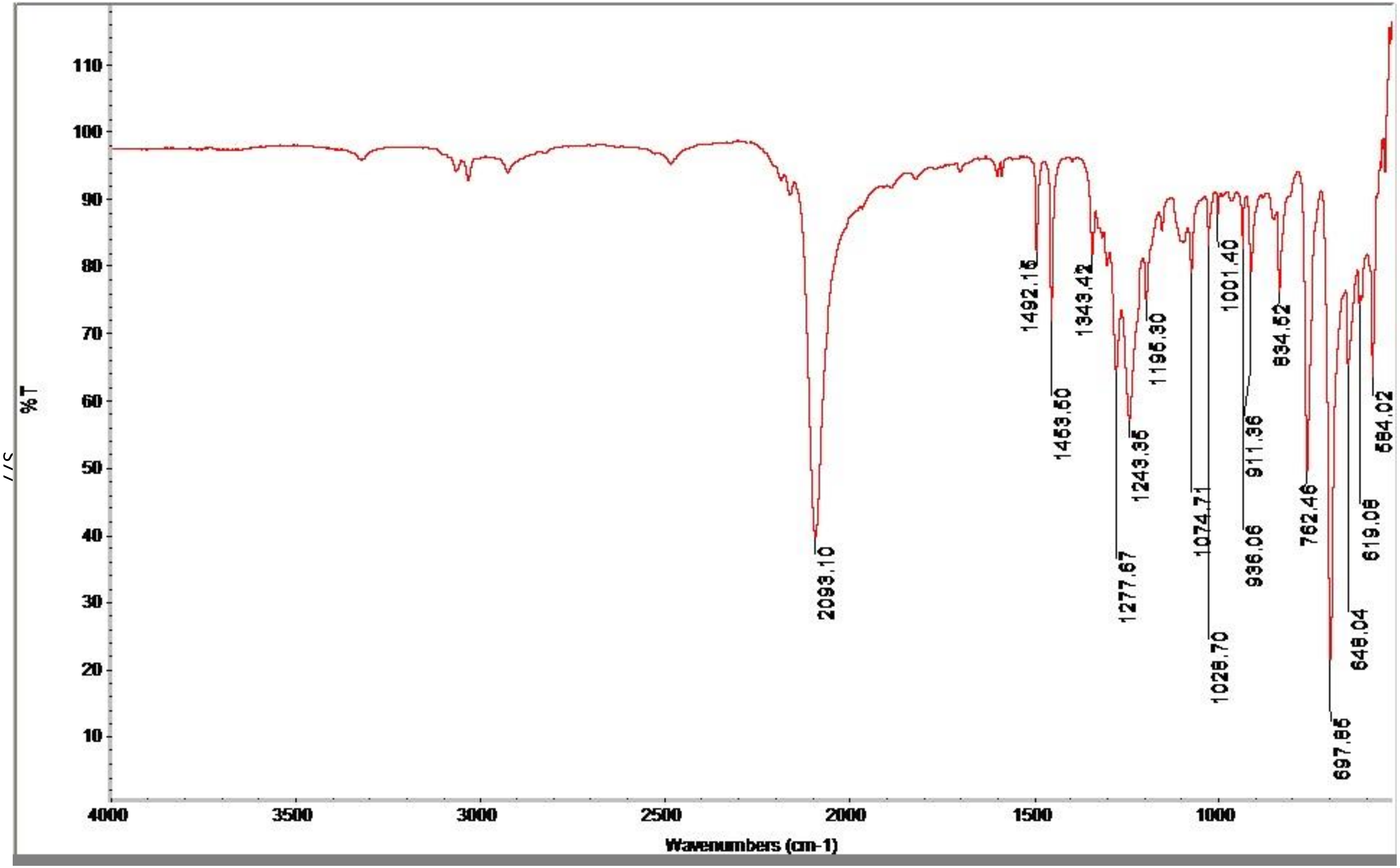

%T
110
100
90
80
70
60
50
40
30
20
10
4000
3500
3000
2500
2000
1500
1000
Wavenumbers (cm-1)
2093,10
1492,15
1453,50
1343,42
1277,67
1243,35
1195,30
1074,71
1028,70
1001,40
936,06
911,36
834,52
762,46
697,85
648,04
619,06
584,02

Data for 1-(1,2-diazidoethyl)-4-cyanobenzene (**4f**)

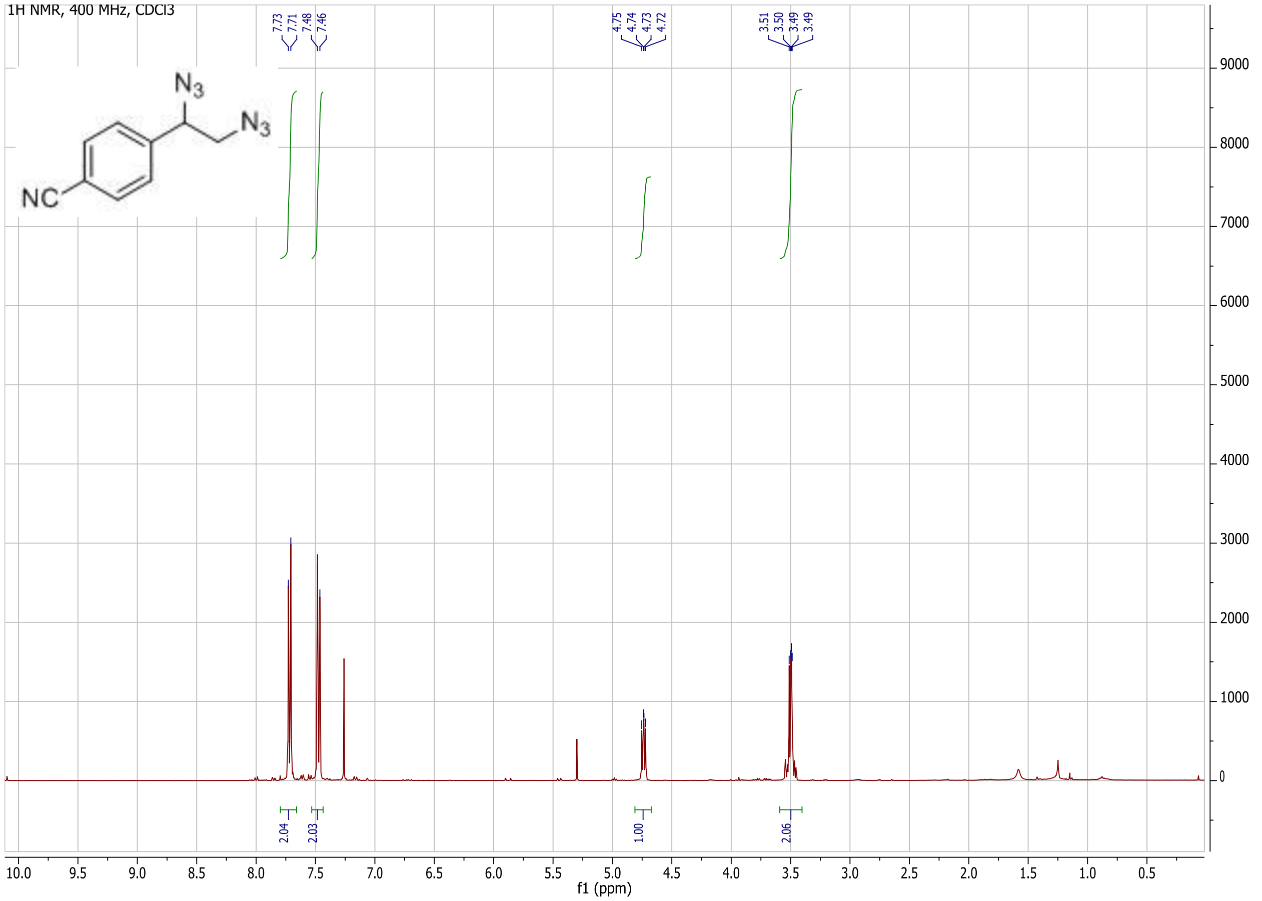

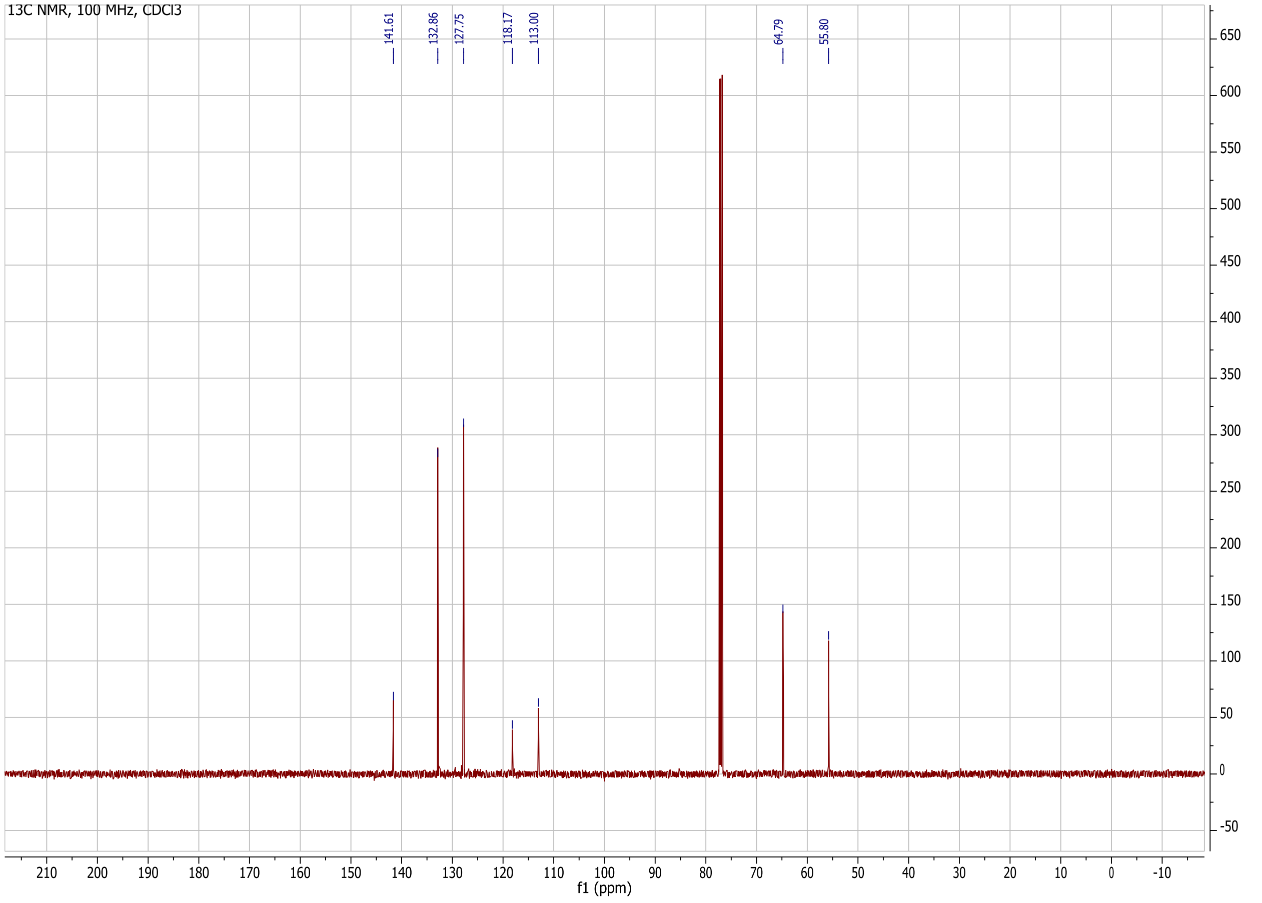

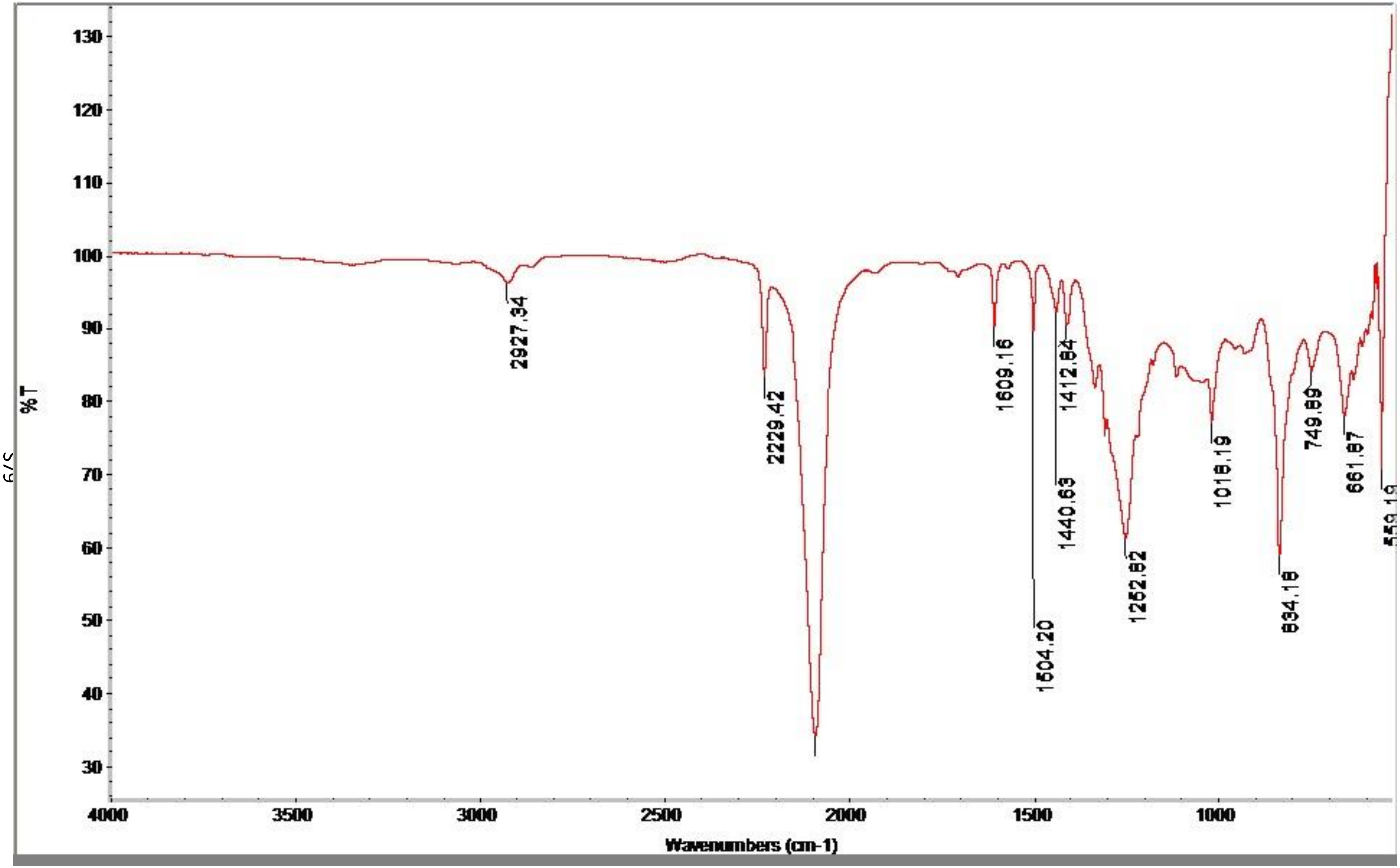
%T
130
120
110
100
90
80
70
60
50
40
30
4000
3500
3000
2500
2000
1500
1000
Wavenumbers (cm-1)
2927,34
2229,42
1609,16
1504,20
1440,63
1412,84
1252,82
1018,19
834,18
749,89
661,87

Data for 1-(1,2-diazidoethyl)-4-trifluorobenzene (**4g**)

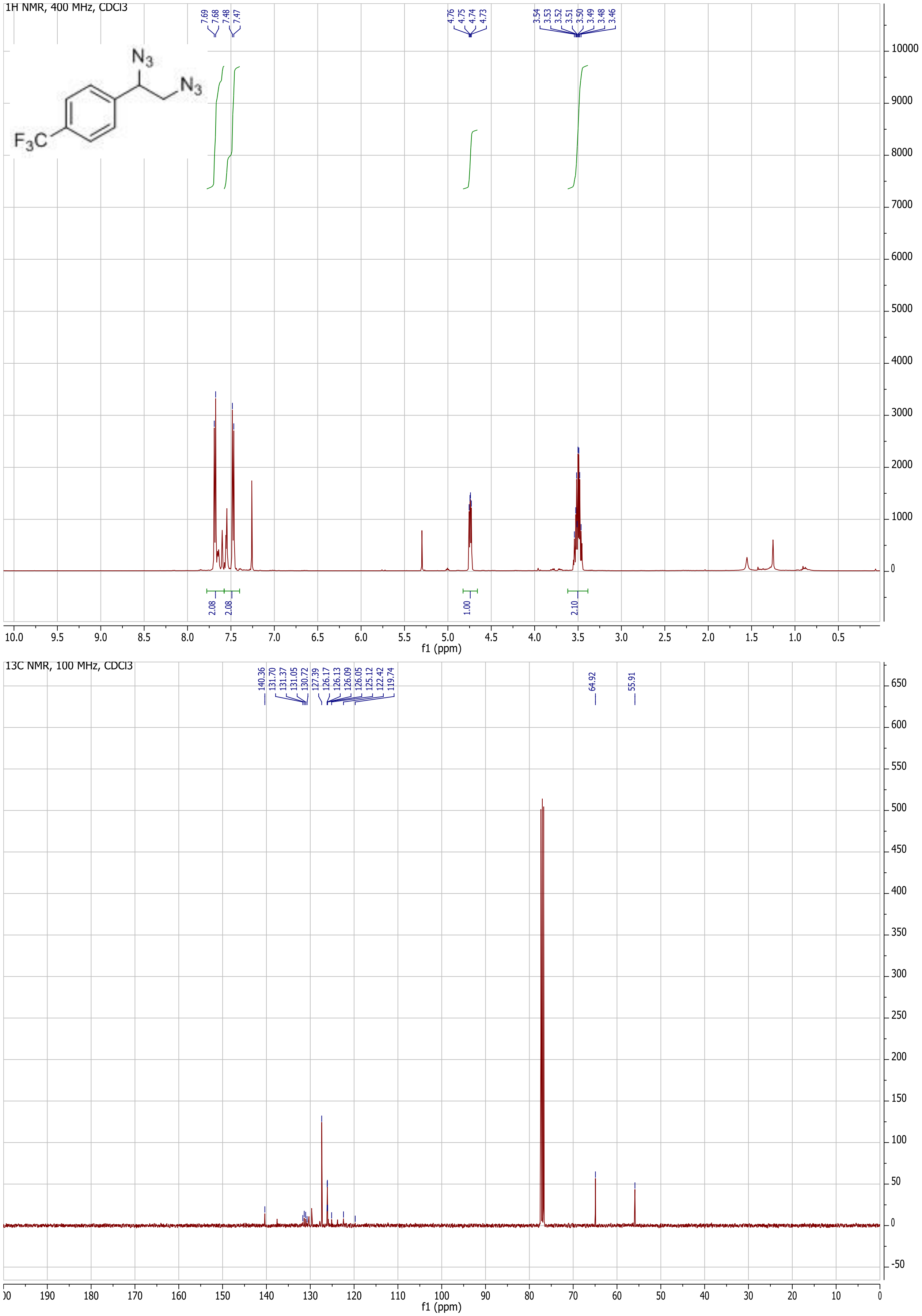

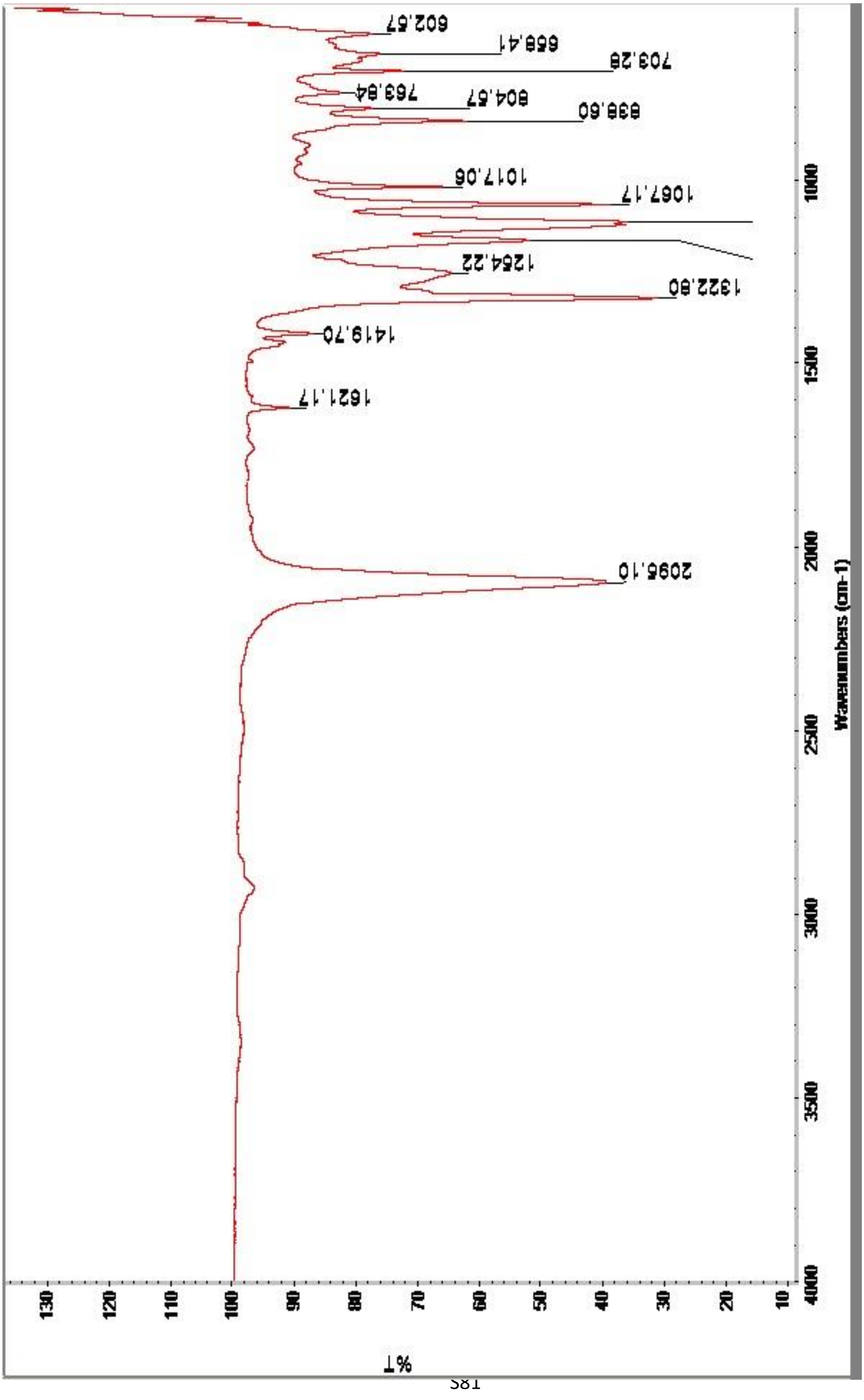
602,57
658,41
703,28
763,84
804,57
838,60
1017,06
1067,17
1254,22
1322,80
1419,70
1621,17
2095,10
Wavenumbers (cm-1)
%T

Data for 1-(1,2-diazidopropan-2-yl)-4-fluorobenzene (**4h**)

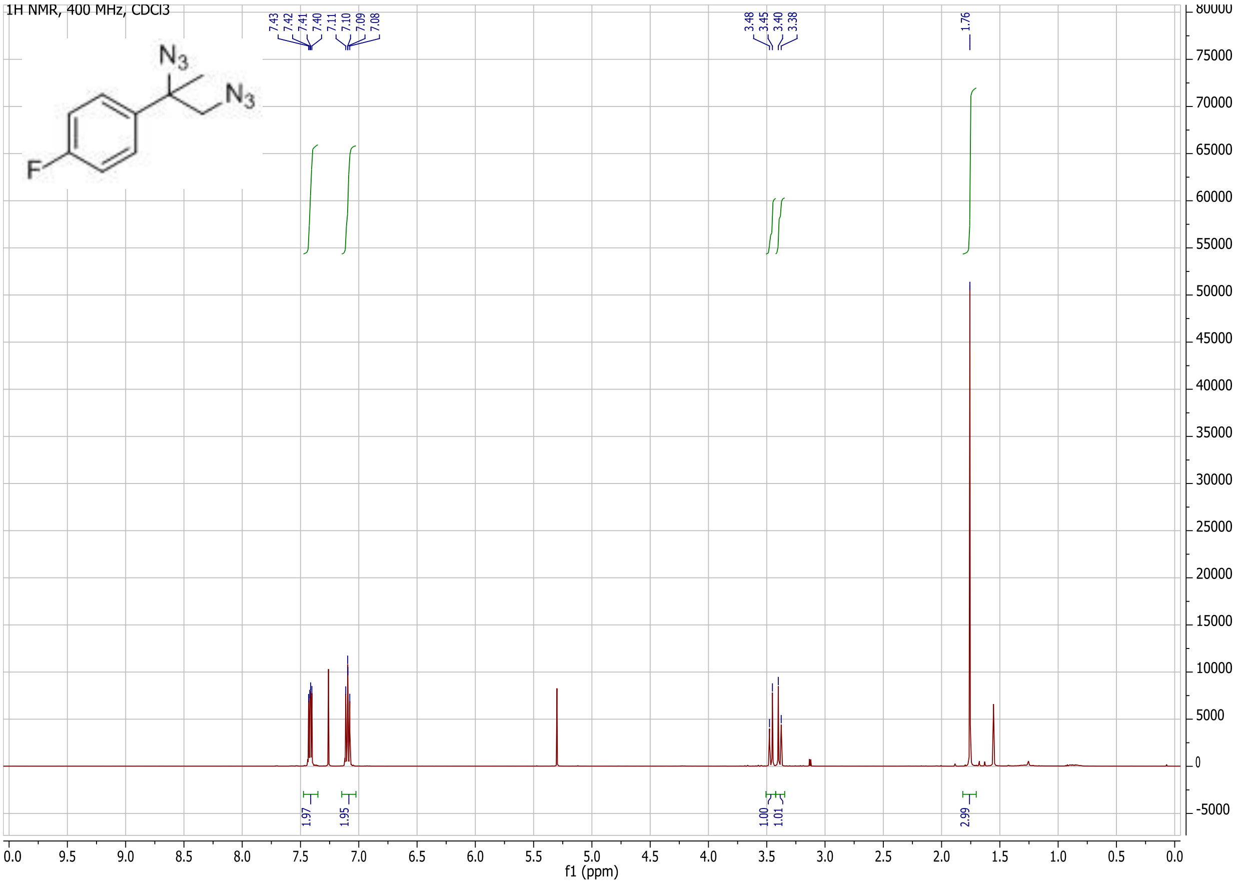

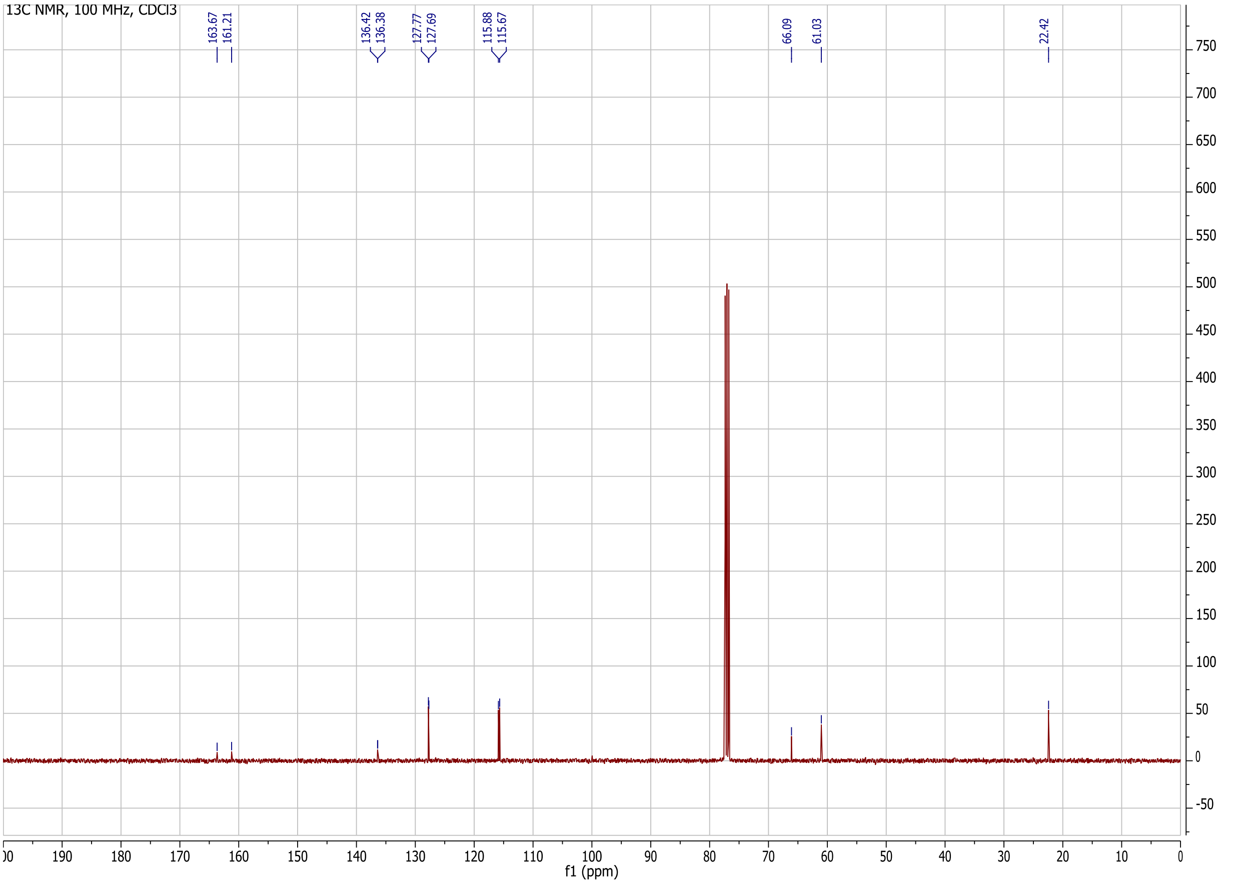

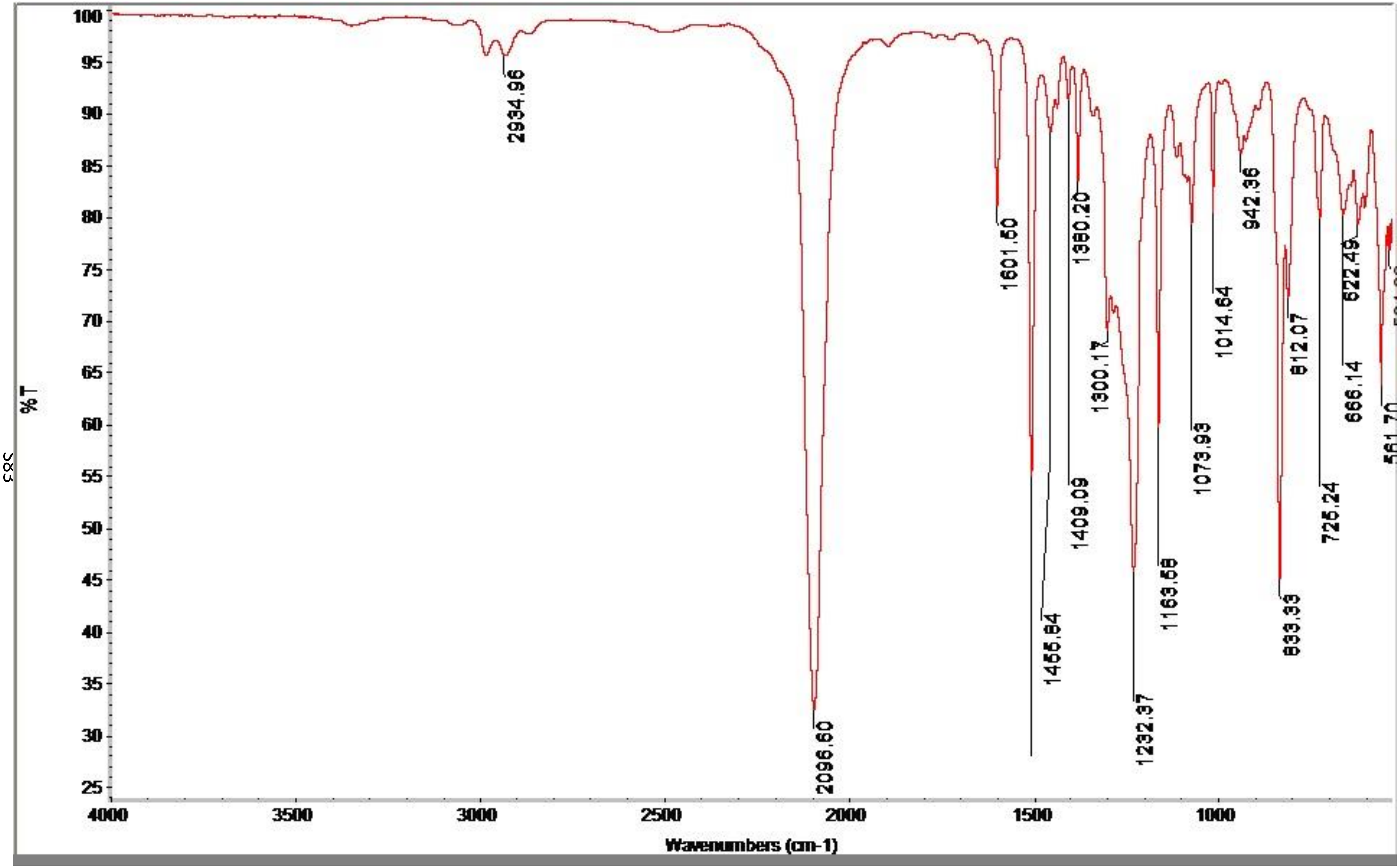
%T
100
95
90
85
80
75
70
65
60
55
50
45
40
35
30
25
4000
3500
3000
2500
2000
1500
1000
Wavenumbers (cm-1)
2934,96
2096,60
1601,50
1455,84
1409,09
1380,20
1300,17
1232,37
1163,58
1073,93
1014,64
942,36
833,33
812,07
725,24
666,14
622,49
561,70

Data for 1,2-diazido-2,3-dihydro-1*H*-indene (**4j**)

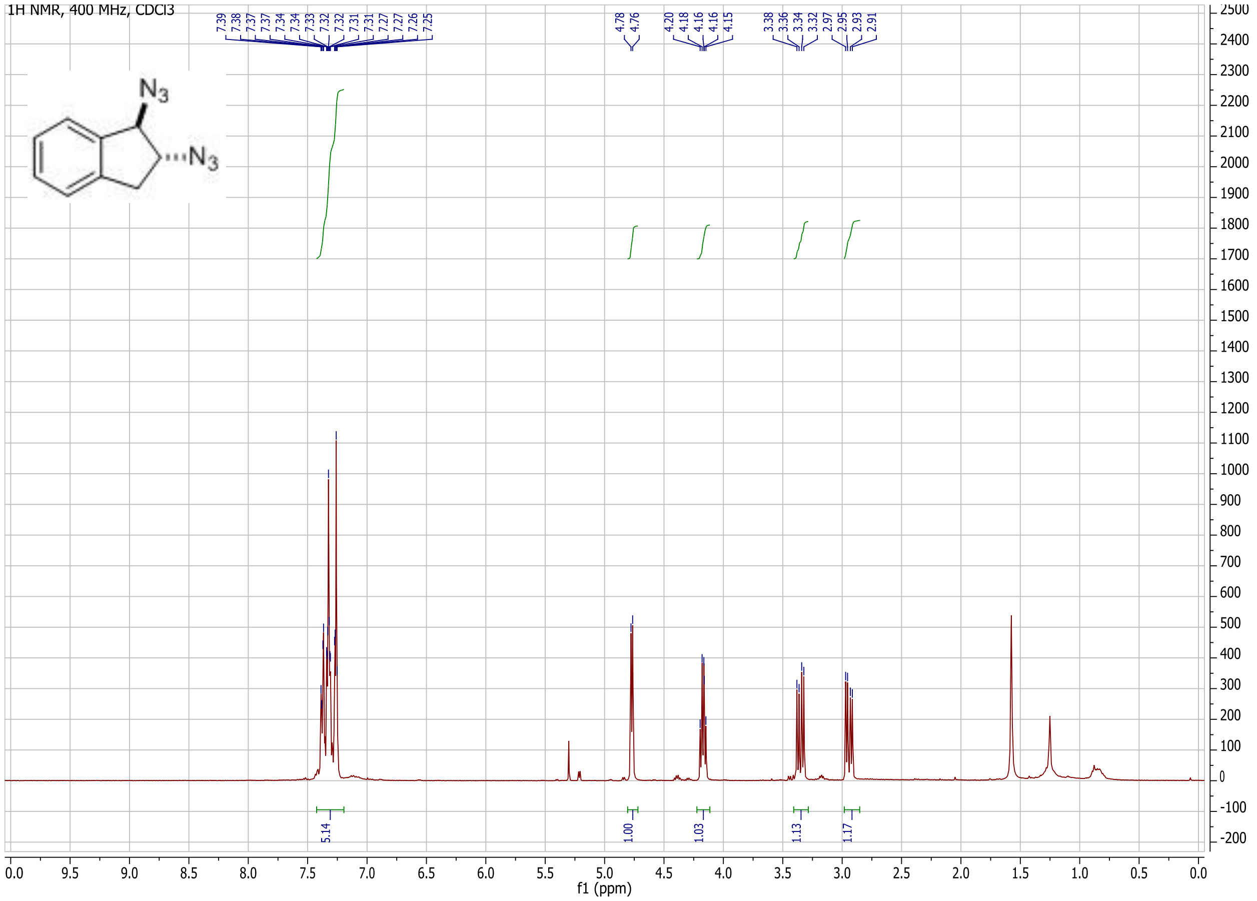

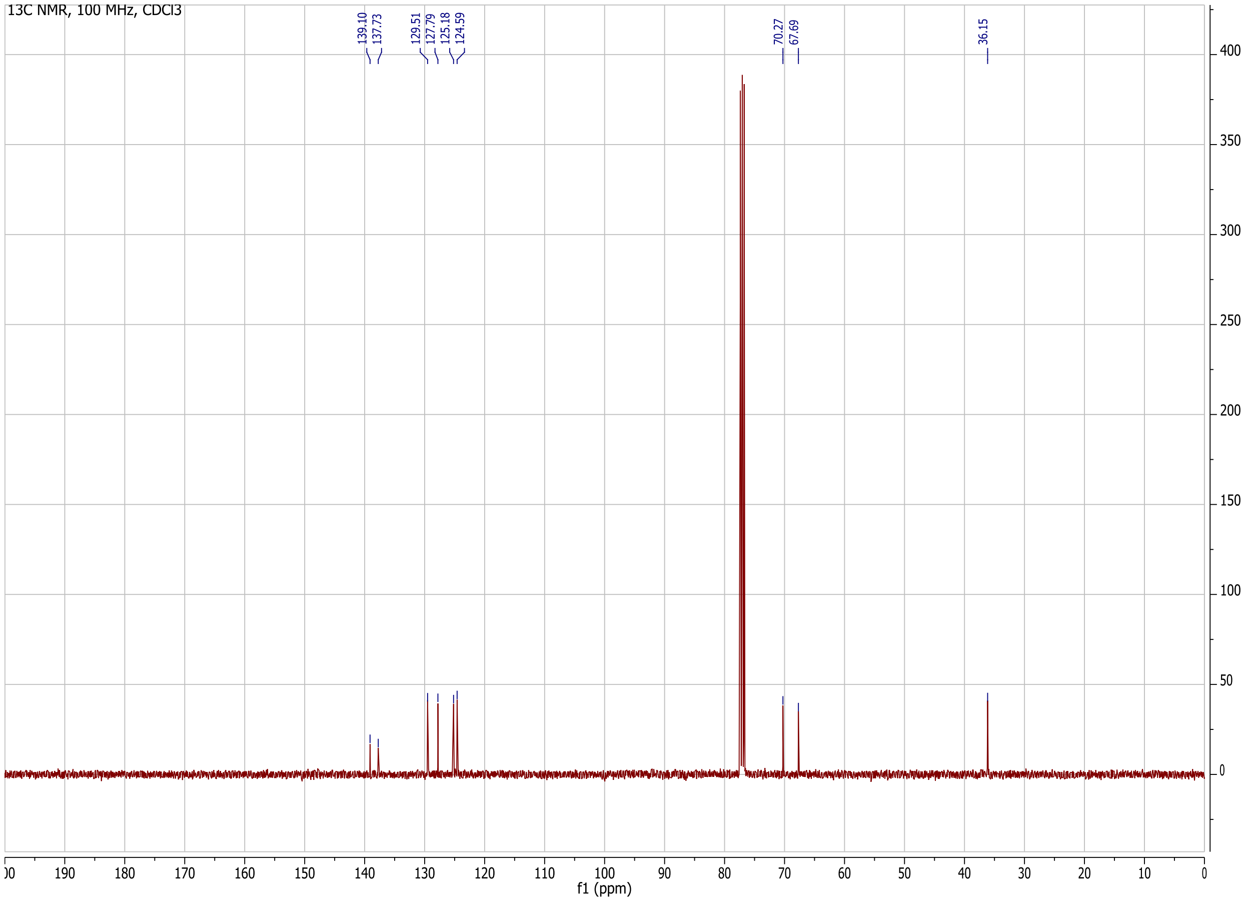

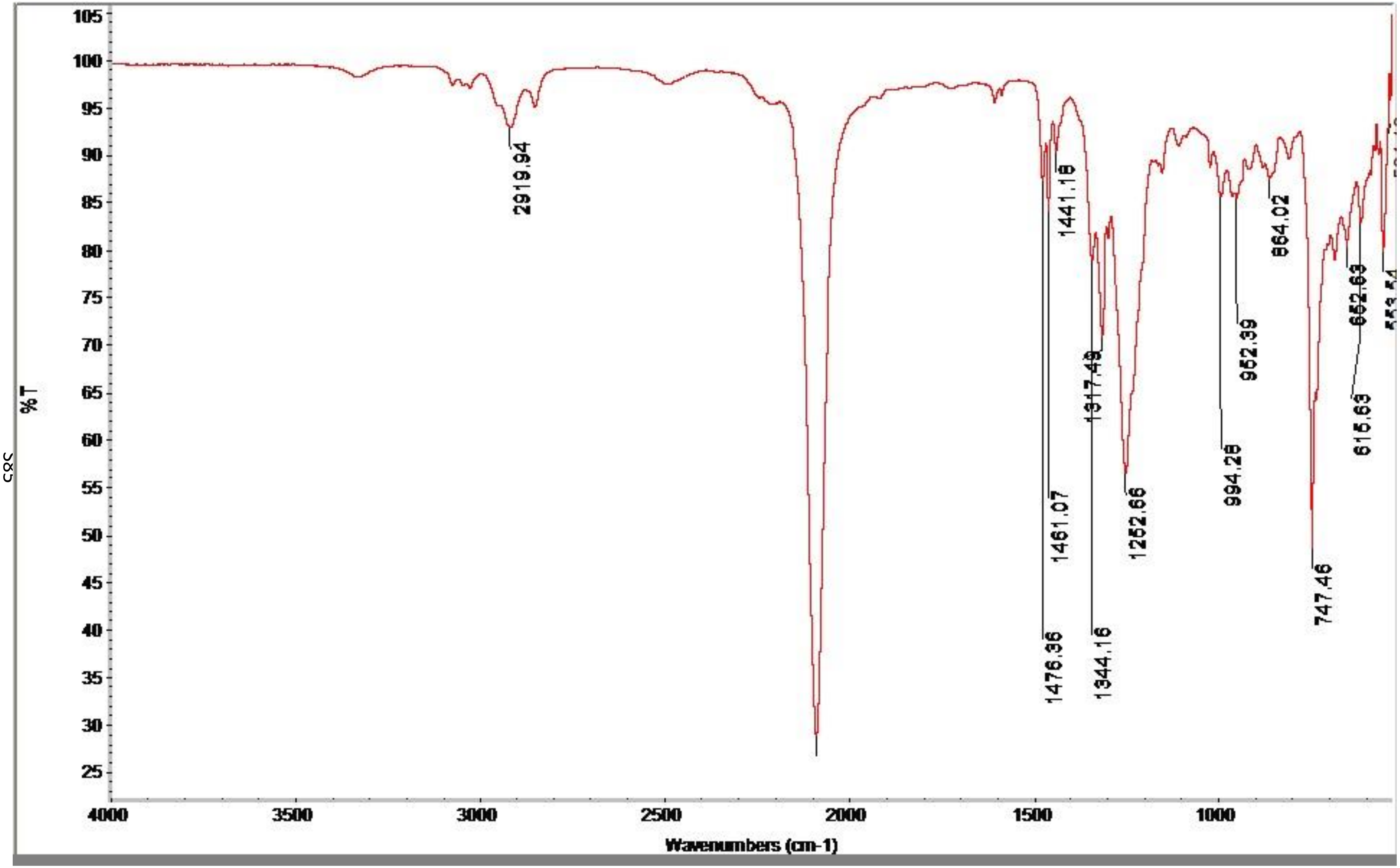

%T
Wavenumbers (cm-1)
2919,94
1476,36
1461,07
1441,18
1344,16
1317,49
1252,66
994,28
952,39
864,02
747,46
652,63
615,63

10 References

[1] Vita, M. V.; Wase, J. *Org. Lett.* **2013**, *15*, 3246-3249
[2] Marrocchi, A.; Minuti, L.; Taticchi, A.; Scheeren, H. W. *Tetrahedron* **2001**, *57*, 4959-4965
[3] a)Pirtsch, M.; Paria, S.; Matsuno, T.; Isobe, H.; Reiser, O. *Chem. Eur. J.* **2012**, *24*, 7336-7340; b) Sauvage, J.-P. *Nouv. J. Chim.* **1985**, *9*, 299-310; c) Chambron, J.-C.; Sauvage, J.-P. *New J. Chem.* **1990**, *14*, 883-890.
[4] Maity, S.; Naveen, T.; Sharma, U.; Maiti, D. *Org. Lett.* **2013**, *15*, 3384-3387
[5] Pérez-Serrano, L.; Blanco-Urgoiti, J.; Casarrubios, L.; Domínguez, G.; Pérez-Castells, J. *J. Org. Chem.* **2000**, *65*, 3513-3519
[6] Bräse, S.; Gil, C.; Knepper, K.; Zimmermann, V. *Angew. Chem. Int. Ed.* **2005**, *44*, 5188 – 5240.
[7] Zhdankin, V. V.; Kuehl, C. J.; Krasutsky, A. P.; Formaneck, M. S.; Bolz, J. T. *Tetrahedron Lett.*, **1994**, *35*, 9677 – 9680.
[8] Tingoli, M.; Tiecco, M.; Testaferri, L.; Temperini, A. *J. Chem. Soc., Chem. Commun.* **1994**, 1883-1884
[9] Subhash, P. C.; Subbaras, Y. T. *Tet. Lett.* **1999**, *40*, 5073-5074
[10]Kamble, D.; Karabal, P. U.; Chouthainale, P. V.; Sudalai, A. *Tetrahedron. Lett.* **2012**, *53*, 4195-4198
[11]Nagorski, R. W.; Brown, R. S. *J. Am. Chem. Soc.* **1992**, *114*, 7773-7779.
[12]Naka, H.; Kanase, N.; Ueno, M.; Kondo, Y. *Chem. Eur. J.* **2008**, *14*, 5267-5274
[13]Zong, X; Zheng, Q.-Z.; Jiao, N. *Org. Bomol. Chem.* **2014**, *12*, 1198-1202
[14]Palmer, M. J.; Kenny, J. A.; Walsgrave, T.; Kawamoto, A. M.; Wills, M. *J. Chem. Soc., Perk. Trans. 1* **2002**, 416-427
[15] Zhao, B.; Yuan, W.; Du, H; Shi, Y. *Org. Lett.* **2007**, *7*, 4943-4945
